# Effect of Preemptive Intervention on Developmental Outcomes Among Infants Showing Early Signs of Autism: A Randomized Clinical Trial of Outcomes to Diagnosis

**DOI:** 10.1001/jamapediatrics.2021.3298

**Published:** 2021-09-20

**Authors:** Andrew J. O. Whitehouse, Kandice J. Varcin, Sarah Pillar, Wesley Billingham, Gail A. Alvares, Josephine Barbaro, Catherine A. Bent, Daniel Blenkley, Maryam Boutrus, Abby Chee, Lacey Chetcuti, Alena Clark, Emma Davidson, Stefanie Dimov, Cheryl Dissanayake, Jane Doyle, Megan Grant, Cherie C. Green, Megan Harrap, Teresa Iacono, Lisa Matys, Murray Maybery, Daniel F. Pope, Michelle Renton, Catherine Rowbottam, Nancy Sadka, Leonie Segal, Vicky Slonims, Jodie Smith, Carol Taylor, Scott Wakeling, Ming Wai Wan, John Wray, Matthew N. Cooper, Jonathan Green, Kristelle Hudry

**Affiliations:** 1 CliniKids, Telethon Kids Institute, Nedlands, Western Australia, Australia; 2 University of Western Australia, Crawley, Western Australia, Australia; 3 Cooperative Research Centre for Living with Autism, Long Pocket, Indooroopilly, Queensland, Australia; 4 School of Allied Health Sciences, Griffith University, Gold Coast, Queensland, Australia; 5 Olga Tennison Autism Research Centre, School of Psychology and Public Health, La Trobe University, Bundoora, Victoria, Australia; 6 Department of Psychology and Counselling, School of Psychology and Public Health, La Trobe University, Bundoora, Victoria, Australia; 7 Perinatal Mental Health and Parenting Research Unit, Division of Psychology and Mental Health, School of Health Sciences, University of Manchester, Manchester, United Kingdom; 8 School of Psychological Science, University of Western Australia, Crawley, Western Australia, Australia; 9 Child and Adolescent Health Service, Child Development Service, West Perth, Western Australia, Australia; 10 Living with Disability Research Centre, College of Science, Health, and Engineering, Victoria, Australia; 11 Australian Centre for Precision Health, Allied Health and Human Movement, University of South Australia, South Australian Health and Medical Research Institute, North Terrace, Adelaide, South Australia, Australia; 12 Children’s Neurosciences, Evelina London Children’s Hospital/Institute of Psychiatry, Psychology and Neuroscience, Kings College London, London, United Kingdom; 13 Division of Neuroscience and Experimental Psychology, School of Biological Sciences, University of Manchester, Manchester, United Kingdom; 14 Manchester Academic Health Science Centre, Manchester University NHS Foundation Trust, Greater Manchester Mental Health NHS Trust, Manchester, United Kingdom

## Abstract

**Importance:**

Intervention for individuals with autism spectrum disorder (ASD) typically commences after diagnosis. No trial of an intervention administered to infants before diagnosis has shown an effect on diagnostic outcomes to date.

**Objective:**

To determine the efficacy of a preemptive intervention for ASD beginning during the prodromal period.

**Design, Setting, and Participants:**

This 2-site, single rater–blinded randomized clinical trial of a preemptive intervention vs usual care was conducted at 2 Australian research centers (Perth, Melbourne). Community sampling was used to recruit 104 infants aged 9 to 14 months showing early behaviors associated with later ASD, as measured by the Social Attention and Communication Surveillance–Revised. Recruitment occurred from June 9, 2016, to March 30, 2018. Final follow-up data were collected on April 15, 2020.

**Interventions:**

Infants were randomized on a 1:1 ratio to receive either a preemptive intervention plus usual care or usual care only over a 5-month period. The preemptive intervention group received a 10-session social communication intervention, iBASIS–Video Interaction to Promote Positive Parenting (iBASIS-VIPP). Usual care comprised services delivered by community clinicians.

**Main Outcomes and Measures:**

Infants were assessed at baseline (approximate age, 12 months), treatment end point (approximate age, 18 months), age 2 years, and age 3 years. Primary outcome was the combined blinded measure of ASD behavior severity (the Autism Observation Scale for Infants and the Autism Diagnostic Observation Schedule, second edition) across the 4 assessment points. Secondary outcomes were an independent blinded clinical ASD diagnosis at age 3 years and measures of child development. Analyses were preregistered and comprised 1-tailed tests with an α level of .05.

**Results:**

Of 171 infants assessed for eligibility, 104 were randomized; 50 infants (mean [SD] chronological age, 12.40 [1.93] months; 38 boys [76.0%]) received the iBASIS-VIPP preemptive intervention plus usual care (1 infant was excluded after randomization), and 53 infants (mean [SD] age, 12.38 [2.02] months; 32 boys [60.4%]) received usual care only. A total of 89 participants (45 in the iBASIS-VIPP group and 44 in the usual care group) were reassessed at age 3 years. The iBASIS-VIPP intervention led to a reduction in ASD symptom severity (area between curves, −5.53; 95% CI, −∞ to −0.28; *P* = .04). Reduced odds of ASD classification at age 3 years was found in the iBASIS-VIPP group (3 of 45 participants [6.7%]) vs the usual care group (9 of 44 participants [20.5%]; odds ratio, 0.18; 95% CI, 0-0.68; *P* = .02). Number needed to treat to reduce ASD classification was 7.2 participants. Improvements in caregiver responsiveness and language outcomes were also observed in the iBASIS-VIPP group.

**Conclusions and Relevance:**

Receipt of a preemptive intervention for ASD from age 9 months among a sample of infants showing early signs of ASD led to reduced ASD symptom severity across early childhood and reduced the odds of an ASD diagnosis at age 3 years.

**Trial Registration:**

http://anzctr.org.au identifier: ACTRN12616000819426.

## Key Points

**Question:**

Does preemptive intervention compared with usual care reduce the severity of autism symptoms and the likelihood of an autism spectrum disorder (ASD) diagnosis in infants showing early signs of ASD?

**Findings:**

In this randomized clinical trial of 103 infants showing early behavioral signs of ASD, preemptive intervention led to a statistically significant reduction in the severity of ASD behaviors across early childhood. Infants who received the preemptive intervention had lower odds of meeting diagnostic criteria for ASD (7%) than those who received usual care (21%) at age 3 years, with a number needed to treat of 7 participants.

**Meaning:**

This study found that a preemptive intervention reduced ASD diagnostic behaviors when used at the time atypical development first emerges during infancy.

## Teaser

This randomized clinical trial examines whether preemptive intervention compared with usual care reduces the severity of autism symptoms and the odds of an autism spectrum disorder diagnosis among infants showing early signs of autism.

## Introduction

Autism spectrum disorder (ASD) is a neurodevelopmental disorder characterized by impairments in social interaction and communication as well as a repetitive and/or restricted range of behaviors and interests.^1^ Autism spectrum disorder is emergent in early development but is not typically diagnosed until age 3 years,^2^ and current clinical guidelines^3,4^ highlight diagnosis as a catalyst in the clinical pathway to commence therapeutic intervention. However, interventions beginning during the first 2 years of life, when the first signs of atypical development are observed and the brain is rapidly developing, may lead to an even greater impact on developmental outcomes in later childhood.^5,6^

Previous randomized clinical trials of preemptive interventions have not demonstrated intervention effects on ASD symptom emergence.^7,8,9,10,11,12,13^ However, recent advances in developmental science have provided key insights into potential intervention mechanisms,^14,15,16^ particularly regarding the ways in which adapted caregiver interaction styles can modify the effect of existing infant vulnerabilities in social attention^17,18^ on later development.^19,20,21^ The iBASIS–Video Interaction to Promote Positive Parenting (iBASIS-VIPP) intervention targets these developmental processes using video feedback techniques to increase caregiver awareness of their infant’s individual social communication and guide specific caregiver responses to build infant social engagement and interaction. An initial pilot study found that the iBASIS-VIPP intervention was acceptable to parents and infants.^22^ A randomized clinical trial of 54 infants with an increased familial likelihood of ASD (based on having a sibling with ASD) found that receipt of the iBASIS-VIPP intervention from age 9 months led to a significant reduction in the severity of emerging ASD symptoms over the prodromal period when measured up to age 3 years.^23,24^ However, this initial randomized clinical trial was underpowered to measure treatment effects on categorical ASD diagnosis, so the clinical significance of this finding remains uncertain.

The Australian Infant Communication and Engagement Study^25^ provided the first well-powered test of the iBASIS-VIPP intervention among infants showing early behavioral signs of ASD. At the intervention end point (age 18 months), there was no difference between infants receiving iBASIS-VIPP vs usual care on researcher-administered measures of infant behavior. The current study is an examination of the longitudinal outcomes of the Australian cohort to 24 months after baseline (age 3 years), the time at which categorical ASD diagnosis can be examined. On the basis of theory and clear results from the previous selectively sampled clinical trial,^23,24^ along with the absence of reported harms in that clinical trial and in previous pilot work,^22^ our prespecified directional hypothesis was that use of the iBASIS-VIPP intervention during infancy would reduce ASD symptom severity and the odds of ASD diagnosis and improve a range of developmental outcomes.

## Methods

### Study Design

The study was a 2-site (based in Perth and Melbourne, Australia), single rater–blinded randomized clinical trial of an intervention conducted over a 5-month period with medium-term developmental follow-up. Participants were recruited from June 9, 2016, to March 30, 2018. Assessments were conducted at baseline, 6 months after baseline (treatment end point), 12 months after baseline, and 24 months after baseline. The final 24-month postbaseline assessment was conducted on April 15, 2020. The clinical trial was approved by the human research ethics committees at Princess Margaret Hospital (Perth) and La Trobe University (Melbourne), and each family provided written informed consent. This study followed the Consolidated Standards of Reporting Trials (CONSORT) reporting guideline for randomized clinical trials. The trial protocol is available in Supplement 1, and further methodological details are provided in eMethods 3 in Supplement 2.

### Participants

Families were referred by community clinicians and invited to participate if (1) the infant was between age 9 months and less than 15 months (corrected for prematurity) at eligibility screening, (2) the infant displayed at least 3 of 5 specified behaviors indicating a high likelihood of ASD as defined by the Social Attention and Communication Surveillance–Revised (SACS-R) 12-month checklist,^26,27^ and (3) the primary caregiver spoke sufficient English to participate in the intervention. Families were excluded if (1) the infant had a diagnosed comorbidity known to affect neurological and developmental abilities and/or (2) the family did not intend to remain residents in the local area for the clinical trial duration.

The SACS-R is administered by clinicians to identify infants and children showing early behavioral signs of ASD.^26,28,29^ The checklist on the 12-month version of the SACS-R includes 5 specified behaviors that are evaluated to determine whether the infant has a higher likelihood of ASD: spontaneous eye contact, protodeclarative pointing, social gestures, imitation, and response to name. A pattern of atypical behavior on at least 3 of these items suggests an increased likelihood of an ASD diagnosis in later childhood. In previous community validation studies,^26,30^ when administered repeatedly at ages 12, 18, and 24 months, the SACS-R had excellent psychometric properties for identifying ASD (positive predictive value, 82%-83%; negative predictive value, 98%-99%; sensitivity, 77%-82%; specificity, 99.0%-99.5%). The current study administered the SACS-R at a single assessment point only (between age 9 months and <15 months) as a means of identifying infants eligible for clinical trial entry.

### Randomization and Blinding

Eligible participants were randomized on a 1:1 ratio via a computer algorithm run by a clinical trial coordinator (K.V.), who communicated directly with the clinical team. Infants were randomized to receive either the iBASIS-VIPP intervention plus usual community care or usual community care only. Randomization was performed by minimization stratified by site (Perth or Melbourne), infant sex (male or female), number of behaviors indicating a higher likelihood of ASD on the SACS-R (endorsement of 3, 4, or 5 behaviors), and age range at recruitment (9-11 months or 12-14 months), with randomization determined by a biased coin with a probability of 0.7. The research staff conducting the assessments (S.P., M.B., L.C., S.D., and M.H.) were independent of the clinical teams administering the iBASIS-VIPP intervention (J.D., M.R., C.R., M.G., and S.W.); they were housed in separate buildings and unaware of the nature of the iBASIS-VIPP intervention (including hypothesized treatment mechanisms), the randomization methods, and the group allocations. Because the intervention was parent-mediated, families could not be blinded to group allocation.

### Intervention

The iBASIS-VIPP is a version of the Video Interaction for Promoting Positive Parenting program,^31^ which was modified for the ASD prodrome.^32^ The intervention involved 10 sessions delivered in family homes by a trained therapist (J.D., M.R., C.R., M.G., or S.W.) over a 5-month period. Caregiver-infant interactions were videotaped during each session, which provided the basis for video feedback discussion. Core aspects of the iBASIS-VIPP intervention included (1) a focus on the social-communicative aspects of each parent-infant dyad, (2) viewing of videotaped interaction excerpts that provided positive examples of infant behaviors and responsive caregiver interactions, and (3) therapist framing of observations, assistance with caregiver self-reflection, and focus on change in the caregiver’s communicative responses to the infant (the intervention manual is available in eMethods 1 in Supplement 2). Caregivers were asked to undertake daily home practice using targeted skills when interacting with their infant. Any adverse events associated with the intervention were recorded by the therapist at the end of each session based on clinical observation and solicited parent reporting. The principal investigator (A.J.O.W.) determined whether the event was causally related to the intervention (ie, an adverse effect).

Usual community care comprised services recommended by health professionals within the local community, including a range of allied health services, comprehensive autism interventions, or no services. During the 5-month treatment phase, parents in both groups completed a weekly diary in which they recorded all contact with health professionals external to the study. At the 12-month and 24-month postbaseline assessments, parents were asked to record any community care their infants had received between study assessments. The assessments took place in a research setting at the Telethon Kids Institute (Perth) and La Trobe University (Melbourne).

### Primary Outcomes

The primary outcome was ASD symptom severity over time, which was assessed by 2 direct observation measures that were conceptually analogous and appropriate for different developmental stages; this approach to outcome assessment has been successfully used in previous clinical trials of ASD.^24,32^ At the baseline and treatment end point assessments, the Autism Observation Scale for Infants (AOSI)^33^ was used to measure early behavioral signs associated with ASD. The 19-item version of the AOSI was administered, which includes 16 scoring items (range, 0-38 points, with higher scores indicating higher ASD risk behaviors); a total score of 9 points or higher at age 12 months indicates clinical levels of developmental difference.^33^ The Autism Diagnostic Observation Schedule, second edition (ADOS-2),^34^ was used at the 12-month and 24-month postbaseline assessments to measure ASD behaviors. The ADOS-2 toddler module was administered at the 12-month postbaseline assessment, with the total score (range, 0-28 points) as the outcome variable. At the 24-month postbaseline assessment, 1 of 2 ADOS-2 modules was administered depending on whether children had minimal language (module 1) or phrase-level language (module 2). The ADOS-2 calibrated severity score (range, 1-10 points), which was developed to facilitate comparison across different developmentally staged ADOS-2 modules,^35^ was the outcome variable. Higher ADOS-2 total and calibrated severity scores represent greater severity of ASD symptoms. The interrater reliability of coding in the study was found to be very good for both AOSI scores (intraclass *r* = 0.83-0.88 for 20 videos) and ADOS-2 scores (intraclass *r* = 0.88-0.91 for 29 videos). Further information on interrater reliability is available in eMethods 3 in Supplement 2. Assessors who conducted and coded the AOSI and ADOS-2 assessments (S.P., M.B., L.C., C.C.G., J.S., and K.H.) were blinded to group allocation.

### Secondary Outcomes

#### Clinical ASD Diagnosis

Two independent clinicians (A.C. and L.M.) who were experienced in ASD diagnosis and blinded to group allocation reviewed all clinical information collected on infants attending the 24-month postbaseline assessments (age 3 years). Following a prespecified protocol (eMethods 4 in Supplement 2), the clinicians assessed participant status on each of the 7 diagnostic criteria specified for ASD in the *Diagnostic and Statistical Manual of Mental Disorders* (Fifth Edition) (*DSM-5*)^1^; these criteria were A1 (deficits in social-emotional reciprocity), A2 (deficits in nonverbal communicative behaviors used for social interaction), A3 (deficits in developing, maintaining, and understanding relationships), B1 (stereotyped or repetitive motor movements, use of objects, or speech), B2 (insistence on sameness, inflexible adherence to routines, or ritualized behavior), B3 (highly restricted fixated interests that are abnormal in intensity or focus), and B4 (hyperreactivity or hyporeactivity sensory input or unusual sensory interests). The clinicians used these criteria to reach a consensus diagnostic outcome in the following categories: (1) ASD, indicating that a diagnosis of ASD consistent with *DSM-5* criteria could be made with high confidence; (2) possible ASD, indicating that autistic traits were present but not sufficient to provide a diagnosis of ASD with high confidence; (3) other developmental concerns, indicating that developmental concerns were present but not indicative of ASD; and (4) no developmental concerns, indicating that development was within normal limits. Following the approach used by Green et al,^24^ these categories were analyzed as 3 groups: clinical ASD (representing definite ASD), atypical development (representing possible ASD or other developmental concerns), and typical development (representing no developmental concerns).

### Parent-Child Interaction

The Manchester Assessment of Caregiver-Infant Interaction (MACI)^36^ is a global rating measure of a 6-minute parent/caregiver and infant play session, video coded based on subscales ranging from 1 to 7 points (with higher scores indicating greater quality of parent-child interactions). The prespecified subscales of interest were caregiver sensitive responsiveness, caregiver nondirectiveness, infant attentiveness, and infant positive affect. The interrater reliability of coding in the study was good to high (intraclass *r* = 0.70-0.93 for 29 videos). Further information on interrater reliability is provided in eMethods 3 in Supplement 2. All MACI recording and coding was conducted by assessors (D.B., A.C., D.F.P., and M.W.W.) who were blinded to group allocation.

### Developmental and Parent Outcomes

Assessors (S.P., M.B., L.C., S.D., and M.H.) blinded to group allocation administered the Mullen Scales of Early Learning,^37^ a standardized assessment of developmental abilities. The predefined subscales of interest were receptive language (score range, 0-48 points), expressive language (score range, 0-50 points), visual reception (score range, 0-50 points), and fine motor (score range, 0-49 points); for each subscale, higher scores indicated greater developmental ability. Because of floor effects at baseline,^25^ raw scores were used. The Vineland Adaptive Behavior Scales, second edition (VABS-II)^38^ provided a nonblinded caregiver-reported measure of functional skills that are relevant for everyday living. The communication (score range, 20-160 points) and socialization (score range, 20-160 points) subscales of interest were prespecified using age-normed standard scores, with a mean (SD) of 100 (15) points; higher scores on these subscales indicated greater functional skills. The MacArthur Communicative Development Inventories^39^ provided a nonblinded caregiver-reported measure of early vocabulary. Prespecified outcomes of interest were expressive vocabulary count (score range, 0-678 points), receptive vocabulary count (score range, 0-678 points), and total gestures count (score range, 0-63 points), with higher scores indicating increased skills. The Parenting Sense of Competence (PSOC) scale^40^ was used to measure the caregiver’s own sense of parenting efficacy across 3 subscales: satisfaction (score range, 6-36 points), efficacy (score range, 5-30 points), and interest (score range, 3-18 points), with higher scores indicating greater parental sense of competence.

### Statistical Analysis

The intention-to-treat analysis followed a statistical analysis plan (Supplement 1) that was prespecified in outline before the completion of treatment end point assessments, with final detail completed after the initial analysis of data from the treatment end point but before the unblinding of the 12-month and 24-month data.^41^ All analyses were conducted by clinical trial statisticians (W.B. and M.N.C.). Considering the positive effects and absence of harm found in the initial clinical trial of the iBASIS-VIPP intervention,^25^ analyses were prespecified within a 1-sided superiority framework (using 95% CIs and significance tests).

Using methods from previous research,^24,32^ treatment effect estimates for continuous variables were combined across the 4 assessments using seemingly unrelated regressions,^42^ which were estimated by maximum likelihood using the lavaan package in R software, version 4.03 (R Foundation for Statistical Computing). This approach allows participants with missing values for outcome variables and/or covariates to be included in the model. A Cohen *d* effect size was calculated for each measure using the within-group SD at each assessment. Each analysis was covaried for the relevant baseline score in addition to the specified site, participant age at assessment, number of high-likelihood SACS-R items endorsed, and group allocation. The multiple point estimates were then combined into an area between curves (ABC), reflecting the cumulative between-group difference over time, and a Wald test was used to calculate the individual effect estimates and their parameter covariance. Confidence intervals for the effect size areas were obtained via a bootstrapping with replacement procedure with 1000 resamples. Based on previous research,^23^ the study was powered to detect a 0.52 SD difference (α = .05) in AOSI total score change at treatment end point (using an independent-samples *t* test). At study commencement, no within-subject correlation data were available to perform power analyses of the longitudinal ABC analysis. Assuming a correlation of less than 1, this analysis had greater power than a single time point analysis to detect the same sized effect.^43^

Differences between treatment groups in the attainment of each of the 7 individual *DSM-5* diagnostic criteria were first analyzed using a Fisher exact test followed by a logistic regression analysis, which allowed control for the important covariates (infant age at the 24-month postbaseline assessment [age 3 years], baseline AOSI scores, and sex) incorporated into the analyses of the other primary and secondary outcomes. Autism spectrum disorder diagnostic status was examined as a 3-level variable (ASD, atypical development, and typical development) using a Fisher exact test, and the binary outcome variable (ASD vs no ASD) was investigated using logistic regression analysis and covariates. Only participants who attended the 24-month postbaseline assessment at age 3 years were included in these analyses.

## Results

**Figure 1.**
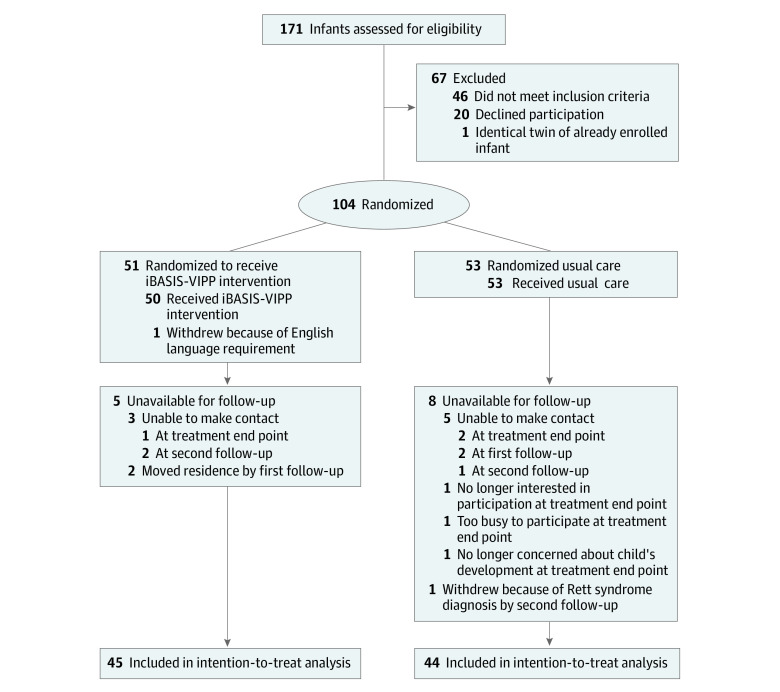
CONSORT Flow Diagram Participants were aged 9 to 15 months during randomization (baseline), 15 to 21 months at the treatment end point (6 months after baseline), 21 to 27 months at the first follow-up assessment (12 months after baseline), and 33 to 39 months at the second follow-up assessment (24 months after baseline). iBASIS-VIPP indicates iBASIS–Video Interaction to Promote Positive Parenting.

A total of 171 infants and their families were assessed for eligibility. Of those, 104 families (66 from Perth and 38 from Melbourne) were enrolled and randomized; 50 infants received the iBASIS-VIPP intervention plus usual care (1 infant was excluded after randomization because the family did not meet the English language requirement^22^), and 53 infants received usual care only (Figure 1). In total, 5 participants in the iBASIS-VIPP group and 8 participants in the usual care group were unavailable for follow-up. After the treatment end point assessment, 1 participant in the usual care group received a genetic diagnosis (Rett syndrome) that met study exclusion criteria; this participant did not participate in further study assessments. The total sample at the final assessment comprised 89 participants (45 in the iBASIS-VIPP group and 44 in the usual care group) who were included in the intention-to-treat analysis.

### Participant Characteristics and Intervention Dosage/Adherence

Infants in the iBASIS-VIPP and usual care groups had similar characteristics at baseline (mean [SD] chronological age, 12.40 [1.93] months vs 12.38 [2.02] months, respectively; 38 of 50 boys [76.0%] vs 32 of 53 boys [60.4%]) (Table 1). In total, 59 of 103 infants (57.3%) had an AOSI score of 9 points or higher at baseline.^25^ Chronological ages at assessment points were similar across the iBASIS and usual care groups (eg, at treatment end point: mean [SD] age, 18.54 [2.12] months vs 18.60 [2.12] months; at 24 months after baseline: mean [SD] age, 36.64 [1.96] months vs 36.54 [2.14] months, respectively) (Table 2; eTable 4 and eTable 5 in Supplement 2).

**Table 1.** Participant Characteristics at Baseline

Characteristic	No./total No. (%)	
	Usual care group (n = 53)	iBASIS-VIPP group (n = 50)
Families		
Annual household income ≥$50 000	44/50 (88.0)	40/42 (95.2)
Mother completed university degree	29/53 (54.7)	33/50 (66.0)
Infant living with both biological parents	52/53 (98.1)	49/50 (98.0)
Infants		
Sex		
Female	21/53 (39.6)	12/50 (24.0)
Male	32/53 (60.4)	38/50 (76.0)
Older sibling with ASD	10/53 (18.9)	10/50 (20.0)
Chronological age, mean (SD), mo	12.38 (2.02)	12.40 (1.93)
Adjusted age, mean (SD), mo	12.31 (2.00)	12.12 (1.98)

Abbreviations: ASD, autism spectrum disorder; iBASIS-VIPP, iBASIS–Video Interaction to Promote Positive Parenting.

**Table 2.** Assessment Data by Intervention Group

Measure	Usual care group (n = 53)				iBASIS-VIPP group (n = 50)			
	Baseline	Treatment end point	12 mo After baseline	24 mo After baseline	Baseline	Treatment end point	12 mo After baseline	24 mo After baseline
Chronological age of participants, mo^a^								
Participants available, No.	53	48	46	44	50	49	46	45
Mean (SD)	12.38 (2.02)	18.60 (2.12)	24.66 (2.17)	36.54 (2.14)	12.40 (1.93)	18.54 (2.12)	24.84 (2.17)	36.64 (1.96)
AOSI score^b^								
Participants available, No.	53	46	NA	NA	51	48	NA	NA
Mean (SD)	9.26 (4.52)	9.52 (5.05)	NA	NA	9.75 (3.86)	9.12 (4.33)	NA	NA
ADOS-2 score								
Participants available, No.	NA	NA	45	44	NA	NA	47	45
Mean (SD)	NA	NA	11.02 (6.36)^b^	5.68 (2.77)^c^	NA	NA	9.40 (5.99)^c^	5.24 (2.28)^d^
MACI subscale score^e^								
Caregiver nondirectiveness								
Participants available, No.	53	47	42	40	51	49	45	43
Mean (SD)	4.09 (1.51)	4.68 (1.42)	4.60 (1.50)	4.55 (1.43)	4.22 (1.64)	4.84 (1.20)	4.89 (1.47)	4.91 (1.38)
Caregiver sensitive responding								
Participants available, No.	53	47	42	40	51	49	45	43
Mean (SD)	4.28 (1.43)	4.81 (1.06)	4.45 (1.23)	4.62 (1.17)	4.25 (1.49)	5.04 (0.91)	4.76 (1.28)	4.79 (1.46)
Infant attentiveness								
Participants available, No.	53	47	42	40	53	47	42	40
Mean (SD)	4.04 (1.36)	4.70 (1.06)	4.19 (1.25)	5.15 (1.10)	3.84 (1.21)	4.43 (1.15)	4.60 (1.14)	5.02 (1.16)
Infant positive affect								
Participants available, No.	53	47	42	40	53	47	42	40
Mean (SD)	3.51 (1.72)	4.40 (1.33)	3.21 (1.91)	4.28 (1.96)	3.31 (1.5)	3.69 (1.54)	3.18 (2.01)	4.02 (1.96)
MSEL subscale raw score^f^								
Expressive language								
Participants available, No.	53	48	45	43	51	49	47	45
Mean (SD)	9.55 (2.52)	14.96 (3.56)	19.60 (5.39)	29.42 (7.27)	9.88 (2.33)	15.35 (3.40)	21.11 (5.60)	30.96 (7.46)
Receptive language								
Participants available, No.	53	48	45	43	51	49	47	45
Mean (SD)	11.00 (2.88)	15.38 (4.49)	21.96 (5.70)	29.63 (7.55)	10.82 (2.85)	16.73 (5.34)	22.30 (6.20)	31.31 (6.27)
Visual reception								
Participants available, No.	52	47	45	43	50	49	47	45
Mean (SD)	15.27 (2.78)	20.32 (3.36)	24.67 (5.53)	34.35 (7.88)	15.48 (3.10)	20.96 (3.05)	26.15 (4.76)	35.78 (6.78)
Fine motor								
Participants available, No.	53	48	45	43	51	49	47	45
Mean (SD)	14.25 (2.83)	18.94 (2.63)	22.44 (4.22)	29.53 (4.59)	14.63 (3.06)	19.73 (2.21)	23.74 (3.08)	30.58 (3.99)
VABS-II subscale standard score^g^								
Communication								
Participants available, No.	44	42	35	40	50	46	42	41
Mean (SD)	80.05 (14.11)	87.36 (16.12)	92.29 (16.16)	93.30 (19.61)	77.10 (15.82)	90.35 (15.07)	93.67 (14.95)	94.73 (13.88)
Socialization								
Participants available, No.	44	39	36	38	48	46	42	43
Mean (SD)	91.20 (11.96)	92.87 (12.27)	92.81 (15.48)	94.61 (18.56)	85.60 (11.58)	93.15 (12.24)	92.67 (13.74)	95.93 (16.32)
MCDI subscale score^h^								
Total expressive vocabulary								
Participants available, No.	38	41	37	38	40	45	43	41
Mean (SD)	1.29 (2.15)	17.44 (28.01)	96.84 (101.03)	414.53 (202.40)	1.73 (2.53)	27.82 (43.51)	129.65 (135.58)	442.71 (189.03)
Total receptive vocabulary								
Participants available, No.	38	41	37	38	40	45	43	41
Mean (SD)	26.24 (30.74)	95.51 (53.73)	244.05 (140.24)	502.45 (163.48)	33.85 (34.28)	127.62 (84.06)	279.86 (164.50)	521.39 (162.45)
Total gestures								
Participants available, No.	40	43	37	39	47	46	39	41
Mean (SD)	10.93 (6.00)	27.60 (9.03)	38.49 (15.08)	49.56 (13.85)	11.06 (5.79)	30.93 (10.88)	41.64 (12.18)	51.80 (9.12)
PSOC subscale score^i^								
Efficacy								
Participants available, No.	40	40	40	40	50	47	44	42
Mean (SD)	21.48 (4.13)	22.23 (3.91)	22.17 (4.18)	22.23 (3.91)	20.40 (4.01)	21.43 (3.77)	21.50 (4.15)	21.57 (4.34)
Interest								
Participants available, No.	44	42	35	40	50	47	44	42
Mean (SD)	14.89 (2.43)	15.31 (2.57)	15.17 (2.54)	14.78 (2.42)	15.28 (2.54)	15.15 (2.46)	15.66 (2.11)	14.73 (3.14)
Satisfaction								
Participants available, No.	44	42	35	40	50	47	44	42
Mean (SD)	23.18 (5.54)	22.27 (6.73)	22.60 (5.55)	22.38 (5.68)	23.52 (4.89)	23.66 (4.33)	22.89 (4.69)	21.64 (5.37)

Abbreviations: ADOS-2, Autism Diagnostic Observation Schedule, second edition; AOSI, Autism Observation Scale for Infants; iBASIS-VIPP, iBASIS–Video Interaction to Promote Positive Parenting; MACI, Manchester Assessment of Caregiver-Infant Interaction; MSEL, Mullen Scales of Early Learning; MCDI, MacArthur Communicative Development Inventories; NA, not applicable; PSOC, Parenting Sense of Competence scale; VABS-II, Vineland Adaptive Behavior Scales, second edition.

^a^ Corrected for prematurity at eligibility screening.

^b^ AOSI scores range from 0-38 points, with higher scores indicating higher ASD risk behaviors; a total score of ≥9 points at age 12 months indicates clinical levels of developmental difference.

^c^ ADOS-2 toddler module total score (range, 0-28 points, with higher scores indicating greater severity of ASD symptoms).

^d^ ADOS-2 calibrated severity score (range, 1-10 points, with higher scores indicating greater severity of ASD symptoms).

^e^ All MACI subscale scores range from 1-7 points, with higher scores indicating greater quality of parent-child interactions.

^f^ MSEL subscale raw score ranges: expressive language, 0-50 points, with higher scores indicating greater expressive language skills; receptive language, 0-48 points, with higher scores indicating receptive language skills; visual reception, 0-50 points, with higher scores indicating greater visual reception skills; and fine motor, 0-49 points, with higher scores indicating greater fine motor skills.

^g^ VABS-II subscale standard score ranges: communication, 20-160 points, with higher scores indicating greater functional communication skills; and socialization, 20-160 points, with higher scores indicating greater functional socialization skills.

^h^ MCDI subscale score ranges: total expressive vocabulary, 0-678 points, with higher scores indicating greater expressive vocabulary; total receptive vocabulary, 0-678 points, with higher scores indicating greater receptive vocabulary; and total gestures, 0-63 points, with higher scores indicating greater total gestures.

^i^ PSOC subscale score ranges: efficacy, 5-30 points, with higher scores indicating greater parental sense of competence; interest, 3-18 points, with higher scores indicating greater parental sense of competence; and satisfaction, 6-36 points, with higher scores indicating greater parental sense of competence.

Therapist fidelity to the manual was evaluated based on 40 videotaped sessions that were randomly selected to balance time point and therapist, and fidelity was found to be high.^25^ Further information on the fidelity monitoring process is provided in eMethods 2 and eMethods 3 in Supplement 2. Participant adherence to the iBASIS-VIPP intervention was high,^25^ and no adverse effects from the intervention were identified. The usual care group received a variety of interventions during the treatment period, ranging from a 1-time parent information seminar to intensive ASD intervention. Community therapy was received by a greater proportion of infants in the usual care group than the iBASIS-VIPP group during the treatment period (27 of 46 infants [58.7%] vs 17 of 49 infants [34.7%], respectively),^25^ between the treatment end point and the 12-month postbaseline assessment (29 of 42 infants [69.0%] vs 21 of 46 infants [45.7%]), and between the 12-month and 24-month postbaseline assessments (26 of 43 infants [60.4%] vs 19 of 45 infants [42.2%]) (eTable 1 in Supplement 2).

### ASD Symptom Severity (Primary Outcome)

**Figure 2.**
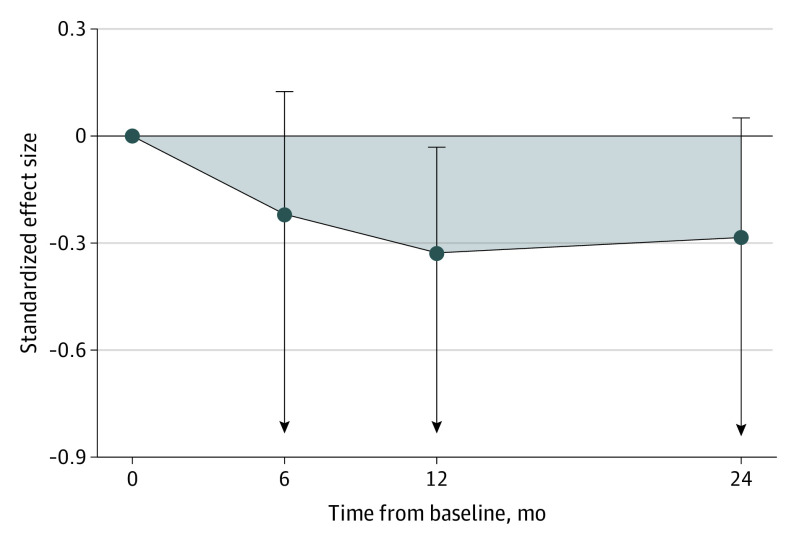
Treatment Effect Over Time for the Primary Outcome of Autism Symptom Severity Effect size estimate with 1-tailed 95% CIs (represented by whiskers). Autism symptom severity was measured by the Autism Observation Scale for Infants and the Autism Diagnostic Observation Schedule–Second Edition. An area between curves (shaded area) below the null indicates a greater reduction in autism symptoms in the iBASIS-VIPP group vs the usual care group. The mean age of participants at assessment points was 12 months (baseline), 18 months (6 months after baseline), 24 months (12 months after baseline), and 36 months (24 months after baseline). iBASIS-VIPP indicates iBASIS–Video Interaction to Promote Positive Parenting.

There was a growing treatment effect (reduced ASD symptom severity) favoring the iBASIS-VIPP group from treatment end point to the 12-month postbaseline assessment, which was largely maintained at the 24-month postbaseline assessment (Figure 2). The combined treatment effect estimate across time points was statistically significant (ABC, −5.53; 95% CI, −∞ to −0.28; *P* = .04).

### ASD Diagnostic Criteria

Independent clinicians who were blinded to group allocation classified 12 participants in the clinical ASD group, 64 participants in the atypical development group, and 13 participants in the typical development group. The ASD diagnostic criteria profiles for these groups are provided in eTable 2 in Supplement 2.

Between-group comparisons using a Fisher exact test found that the iBASIS-VIPP group had lower odds of meeting 1 *DSM-5* criterion than the usual care group (B4 [unusual sensory interests]: odds ratio [OR], 0.21; 95% CI, 0-0.94; *P* = .04) among the 7 criteria for ASD (Table 3). A logistic regression analysis incorporating covariates identified reduced odds of meeting *DSM-5* criteria A1 among the iBASIS-VIPP group (deficits in social-emotional reciprocity: OR, 0.35; 95% CI, 0-0.82; *P* = .02), B1 (stereotyped or repetitive movements: OR, 0.29; 95% CI, 0-0.73; *P* = .02), and B4 (unusual sensory interests: OR, 0.13; 95% CI, 0-0.53; *P* = .02) (Table 3).

**Table 3.** Comparison Between Treatment Groups on Each *DSM-5* Criterion for Autism Spectrum Disorder and Clinical Assessment of Overall Diagnostic Status

Variable	No. (%)		Fisher exact test		Binary logistic regression analysis^a^	
	iBASIS-VIPP group (n = 45)	Usual care group (n = 44)	Odds ratio (95% CI)	*P* value	Odds ratio (95% CI)	*P* value
*DSM-5* criterion						
A1: deficits in social-emotional reciprocity	9 (20.0)	16 (36.4)	0.44 (0-1.08)	.07	0.35 (0-0.82)	.02
A2: deficits in nonverbal communicative behaviors used for social interaction	13 (28.9)	17 (38.6)	0.65 (0-1.49)	.23	0.47 (0-1.08)	.07
A3: deficits in developing, maintaining, and understanding relationships	13 (28.9)	16 (36.4)	0.71 (0-1.65)	.30	0.60 (0-1.31)	.14
B1: stereotyped or repetitive motor movements, use of objects, or speech	7 (15.6)	14 (31.8)	0.40 (0-1.04)	.06	0.29 (0-0.73)	.02
B2: insistence on sameness, inflexible adherence to routines, or ritualized behavior	2 (4.4)	2 (4.5)	0.98 (0-9.40)	.49	1.03 (0-6.21)	.51
B3: highly restricted fixated interests that are abnormal in intensity or focus	3 (6.7)	2 (4.5)	1.49 (0-12.57)	.67	1.16 (0-6.50)	.56
B4: hyperreactivity or hyporeactivity sensory input or unusual sensory interests	2 (4.4)	8 (18.2)	0.21 (0-0.94)	.04	0.13 (0-0.53)	.02
Diagnosis						
ASD	3 (6.7)	9 (20.5)	NA	.07	0.18 (0-0.68)^b^	.02
Atypical development	37 (82.2)	27 (61.4)	NA	NA	NA	NA
Typical development	5 (11.1)	8 (18.2)	NA	NA	NA	NA

Abbreviations: ASD, autism spectrum disorder; *DSM-5*, *Diagnostic and Statistical Manual of Mental Disorders* (Fifth Edition); iBASIS-VIPP, iBASIS–Video Interaction to Promote Positive Parenting; NA, not applicable.

^a^ The binary logistic regression analysis incorporated the following covariates: infant age at the 24-month postbaseline assessment, baseline score on the Autism Observation Scale for Infants, and infant sex.

^b^ The binary logistic regression analysis comparing ASD vs no ASD incorporated the following covariates: infant age at the 24-month postbaseline assessment, baseline score on the Autism Observation Scale for Infants, and infant sex.

No difference between groups was found in the 3-level diagnostic classification (ASD, atypical development, and typical development) using the Fisher exact test (Table 3). However, logistic regression analysis of the binary clinical diagnosis outcome (ASD vs no ASD) incorporating covariates identified reduced odds of ASD classification in the iBASIS-VIPP group (3 of 45 participants [6.7%]) compared with the usual care group (9 of 44 participants [20.5%]; OR, 0.18; 95% CI, 0-0.68; *P* = .02) (Table 3; eFigure 1 in Supplement 2). The number needed to treat to reduce an ASD classification was 7.2 participants. The characteristics of children who met the criteria for ASD at age 3 years are available in eTable 3 in Supplement 2.

### Developmental and Parental Outcomes

 With regard to parent-child interaction, the initial effect of the iBASIS-VIPP intervention on increasing scores on the MACI caregiver sensitive responsiveness subscale began to attenuate at the 24-month postbaseline assessment. The combined treatment effect was statistically significant (ABC, 5.02; 95% CI, 0.02 to ∞). There was no treatment effect on the MACI subscales of caregiver nondirectiveness (ABC, 3.59; 95% CI, −1.80 to ∞), infant attentiveness (ABC, 2.09; 95% CI, −3.35 to ∞), and infant positive affect (ABC, −2.86; 95% CI, −8.30 to ∞). Table 2 presents longitudinal data for secondary outcomes, and eFigure 2 in Supplement 2 shows ABC results.

A consistent pattern of point estimates in favor of the iBASIS-VIPP group on Mullen Scales of Early Learning subscales was observed, but the combined effect estimates were nonsignificant for the receptive language (ABC, 4.00; 95% CI, −1.01 to ∞), expressive language (ABC, 1.55; 95% CI, −3.31 to ∞), visual reception (ABC, 3.20; 95% CI, −1.94 to ∞), and fine motor (ABC, 3.75; 95% CI, −0.89 to ∞) subscales. A similar pattern favoring the iBASIS-VIPP group (but with CIs crossing the null) was observed for VABS-II measures of functional communication (ABC, 6.21; 95% CI, −0.09 to ∞) and functional socialization (ABC, 6.26; 95% CI, −0.20 to ∞) skills. The iBASIS-VIPP group had greater improvement on the nonblinded caregiver-reported MacArthur Communicative Development Inventories subscales measuring expressive vocabulary (ABC, 8.21; 95% CI, 2.15 to ∞), receptive vocabulary (ABC, 8.10; 95% CI, 1.60 to ∞), and gestures (ABC, 6.56; 95% CI, 1.17 to ∞). There was no effect of treatment group on the efficacy (ABC, −1.62; 95% CI, −6.83 to ∞), interest (ABC, 0.18; 95% CI, −5.17 to ∞), and satisfaction (ABC, 0.53; 95% CI, −4.81 to ∞) subscales of the PSOC.

## Discussion

In this randomized clinical trial, a preemptive intervention for infants showing early behavioral signs of ASD led to a significant reduction in the severity of ASD behaviors when summed over the 2 years between baseline and the study end point at age 3 years. These effects were small in extent, and their clinical significance is uncertain. However, intervention effects were observed across longitudinal points and on related behavioral outcomes, such as parent-reported language development (as measured by the MacArthur Communicative Development Inventories). Although modest in size, this consistent pattern of intervention effects observed across developmental domains likely contributed to the best-estimate clinical judgments of diagnosis and the reduced odds of the iBASIS-VIPP group meeting *DSM-5* diagnostic criteria for ASD at age 3 years.

To our knowledge, this randomized clinical trial is the first to demonstrate that a preemptive intervention for infants showing early signs of ASD led to a small but enduring reduction in ASD symptom severity and reduced odds of ASD diagnosis in early childhood. Recent theoretical accounts^14,15,16^ of the developmental emergence of ASD make a distinction between early life perturbations in the functioning of brain systems and neurocognitive mechanisms that can moderate the consequences of these perturbations for later phenotypic outcomes. Early disruptions can be amplified over time by their interaction with neurocognitive mechanisms to channel developmental trajectories into certain behavioral phenotypes, such as ASD. Of importance to intervention development, it is hypothesized that neurocognitive mechanisms, such as social attention and engagement, can also act as resilience factors by creating more adaptive learning experiences for the child, with potential downstream effects on behavioral development.^14,15^ The observed increase in caregiver sensitive responding during infant interactions (as measured by the MACI sensitive responding subscale), coupled with the decrease in ASD symptom severity, is consistent with results from the previous clinical trial of iBASIS-VIPP^24^ and findings from a clinical trial of an intervention among older children with ASD,^32^ providing additional clinical research evidence to support these theoretical accounts of ASD.

### Strengths and Limitations

This study has several strengths. The findings from the clinical trial are strengthened by a moderate sample size that generated adequate statistical power, high participant retention across 4 assessment points spanning 2 years, vigilant blinding of the assessors who conducted assessments and coded videos, and a prespecified analysis plan. It is also important to highlight certain aspects of the statistical analysis plan. Given the favorable findings from the previous study of the iBASIS-VIPP intervention^24^ and the directional hypotheses, the statistical analysis plan prespecified 1-tailed tests with an α level of .05 to reduce the possibility of type II errors.^44^ Furthermore, because we prespecified individual outcome measures for conceptually different domains (rather than multiple measures of the same domain), the analysis plan did not incorporate corrections for multiple comparisons. We note that the between-group comparison for the primary outcome would have been lower than conventional statistical significance thresholds for 2-tailed testing, although the diagnosis classification outcome would have remained significant (Table 3). However, we also note that the treatment effects observed across a broad range of child outcome measures consistently favored the iBASIS-VIPP group in direction and extent and were consistent with the findings of the previous clinical trial of this intervention.^24^ This observation provides confidence in the robust pattern of effects and suggests that the use of prespecified 1-tailed tests did not risk type I error in our reporting.

This study also has limitations. Blinded diagnostic judgments were conducted at the predefined point of age 3 years. Although ASD diagnostic classification at age 3 years is known to be relatively stable across childhood,^45^ it is possible that a small proportion of children may change diagnostic categories if reassessed at later times. Follow-up of these children in later childhood, when the behaviors for ASD and other neurodevelopmental conditions may be more apparent and distinguishable,^46^ will be important to determining the longer-term clinical significance of the intervention effects observed in the current study.

## Conclusions

In this randomized clinical trial, the combination of a significant treatment effect with maintenance up to 18 months after intervention provides initial evidence of efficacy for a new clinical model that uses a specific developmentally focused intervention among infants at higher likelihood of developing ASD. The relatively low therapeutic intensity and the absence of adverse effects are important for its wider adoption by the service system. A cost-effectiveness analysis of the entire treatment pathway (incorporating screening and service delivery) and modeling of longer-term childhood and adulthood outcomes is an important next step to determine the feasibility and value of this clinical model.

## Notes

Supplement 1.
Trial Protocol and Statistical Analysis Plan

Supplement 2. **eMethods 1.** iBASIS-VIPP Intervention Protocol **eMethods 2.** iBASIS-VIPP Intervention Fidelity Rating Scale **eMethods 3.** Additional Detail on Methods **eMethods 4.** Protocol for Clinical Best Estimate Judgment of Case Diagnosis **eTable 1.** Usual Community Care Received by Participants in the iBASIS-VIPP and Usual Care Groups That Was Not Associated With the Clinical Trial **eTable 2.** Consensus Diagnostic Groups by *DSM-5* Diagnostic Criteria **eTable 3.** Outcome Data for Children Meeting *DSM-5* Criteria for Autism Spectrum Disorder **eTable 4.** Primary and Secondary Outcome Data Across Assessment Points by Site **eTable 5.** Primary and Secondary Outcome Data Across Assessment Points by Sibling Status **eFigure 1.** Forest Plot of Comparison Between Treatment Groups by Autism Spectrum Disorder Diagnostic Criteria **eFigure 2.** Area Between Curves for Secondary Outcomes
eReferences

Supplement 3.
Data Sharing Statement

## References

[1] American Psychiatric Association. Diagnostic and Statistical Manual of Mental Disorders. 5th ed. American Psychiatric Association; 2013.

[2] van ‘t Hof M, Tisseur C, van Berckelear-Onnes I, . Age at autism spectrum disorder diagnosis: a systematic review and meta-analysis from 2012 to 2019. Autism. 2021;25(4):862-873. doi:10.1177/1362361320971107 33213190

[3] Hyman SL, Levy SE, Myers SM; Council on Children With Disabilities, Section on Developmental and Behavioral Pediatrics. Identification, evaluation, and management of children with autism spectrum disorder. Pediatrics. 2020;145(1):e20193447. doi:10.1542/peds.2019-3447 31843864

[4] Sandbank M, Bottema-Beutel K, Woynaroski T. Intervention recommendations for children with autism in light of a changing evidence base. JAMA Pediatr. 2021;175(4):341-342. doi:10.1001/jamapediatrics.2020.4730 33165523

[5] Whitehouse AJO. Elizabeth Usher Memorial Lecture: rethinking the clinical pathway for autism spectrum disorder and challenging the status quo. Int J Speech Lang Pathol. 2017;19(3):208-217. doi:10.1080/17549507.2016.1276963 28084105

[6] Green J. Intervention during the prodromal stages of autism spectrum disorders. In: Chawarska K, Volkmar FR, eds. Autism Spectrum Disorder in the First Years of Life: Research, Assessment, and Treatment. Guilford Press; 2020:247-275.

[7] Carter AS, Messinger DS, Stone WL, Celimli S, Nahmias AS, Yoder P. A randomized controlled trial of Hanen’s ‘More Than Words’ in toddlers with early autism symptoms. J Child Psychol Psychiatry. 2011;52(7):741-752. doi:10.1111/j.1469-7610.2011.02395.x 21418212 PMC4783130

[8] Rogers SJ, Estes A, Lord C, . Effects of a brief Early Start Denver Model (ESDM)–based parent intervention on toddlers at risk for autism spectrum disorders: a randomized controlled trial. J Am Acad Child Adolesc Psychiatry. 2012;51(10):1052-1065. doi:10.1016/j.jaac.2012.08.003 23021480 PMC3487718

[9] Kasari C, Siller M, Huynh LN, . Randomized controlled trial of parental responsiveness intervention for toddlers at high risk for autism. Infant Behav Dev. 2014;37(4):711-721. doi:10.1016/j.infbeh.2014.08.007 25260191 PMC4355997

[10] Baranek GT, Watson LR, Turner-Brown L, . Preliminary efficacy of adapted responsive teaching for infants at risk of autism spectrum disorder in a community sample. Autism Res Treat. 2015;2015:386951. doi:10.1155/2015/386951 25648749 PMC4306223

[11] Jones EJH, Dawson G, Kelly J, Estes A, Webb SJ. Parent-delivered early intervention in infants at risk for ASD: effects on electrophysiological and habituation measures of social attention. Autism Res. 2017;10(5):961-972. doi:10.1002/aur.1754 28244271 PMC5993545

[12] Watson LR, Crais ER, Baranek GT, . Parent-mediated intervention for one-year-olds screened as at-risk for autism spectrum disorder: a randomized controlled trial. J Autism Dev Disord. 2017;47(11):3520-3540. doi:10.1007/s10803-017-3268-0 28861651

[13] Rogers SJ, Estes A, Vismara L, . Enhancing low-intensity coaching in parent implemented Early Start Denver Model intervention for early autism: a randomized comparison treatment trial. J Autism Dev Disord. 2019;49(2):632-646. doi:10.1007/s10803-018-3740-5 30203308

[14] Klin A, Micheletti M, Klaiman C, Shultz S, Constantino JN, Jones W. Affording autism an early brain development re-definition. Dev Psychopathol. 2020;32(4):1175-1189. doi:10.1017/S0954579420000802 32938507 PMC7880583

[15] Johnson MH, Charman T, Pickles A, Jones EJH. Annual research review: anterior modifiers in the emergence of neurodevelopmental disorders (AMEND)—a systems neuroscience approach to common developmental disorders. J Child Psychol Psychiatry. 2021;62(5):610-630. doi:10.1111/jcpp.13372 PMC8609429 33432656

[16] Constantino JN, Charman T, Jones EJH. Clinical and translational implications of an emerging developmental substructure for autism. Annu Rev Clin Psychol. 2021;17:365-389. doi:10.1146/annurev-clinpsy-081219-110503 33577349 PMC9014692

[17] Jones W, Klin A. Attention to eyes is present but in decline in 2-6–month-old infants later diagnosed with autism. Nature. 2013;504(7480):427-431. doi:10.1038/nature12715 24196715 PMC4035120

[18] Elsabbagh M, Mercure E, Hudry K, ; BASIS Team. Infant neural sensitivity to dynamic eye gaze is associated with later emerging autism. Curr Biol. 2012;22(4):338-342. doi:10.1016/j.cub.2011.12.056 22285033 PMC3314921

[19] Wan MW, Green J, Elsabbagh M, Johnson M, Charman T, Plummer F; BASIS Team. Quality of interaction between at-risk infants and caregiver at 12-15 months is associated with 3-year autism outcome. J Child Psychol Psychiatry. 2013;54(7):763-771. doi:10.1111/jcpp.12032 23227853

[20] Tamis-LeMonda CS, Bornstein MH, Baumwell L. Maternal responsiveness and children’s achievement of language milestones. Child Dev. 2001;72(3):748-767. doi:10.1111/1467-8624.00313 11405580

[21] Siller M, Sigman M. The behaviors of parents of children with autism predict the subsequent development of their children’s communication. J Autism Dev Disord. 2002;32(2):77-89. doi:10.1023/A:1014884404276 12058846

[22] Green J, Wan MW, Guiraud J, ; BASIS Team. Intervention for infants at risk of developing autism: a case series. J Autism Dev Disord. 2013;43(11):2502-2514. doi:10.1007/s10803-013-1797-8 23532347

[23] Green J, Charman T, Pickles A, ; BASIS Team. Parent-mediated intervention versus no intervention for infants at high risk of autism: a parallel, single-blind, randomised trial. Lancet Psychiatry. 2015;2(2):133-140. doi:10.1016/S2215-0366(14)00091-1 26359749 PMC4722333

[24] Green J, Pickles A, Pasco G, ; British Autism Study of Infant Siblings (BASIS) Team. Randomised trial of a parent-mediated intervention for infants at high risk for autism: longitudinal outcomes to age 3 years. J Child Psychol Psychiatry. 2017;58(12):1330-1340. doi:10.1111/jcpp.12728 28393350 PMC5724485

[25] Whitehouse AJO, Varcin KJ, Alvares GA, . Pre-emptive intervention versus treatment as usual for infants showing early behavioural risk signs of autism spectrum disorder: a single-blind, randomised controlled trial. Lancet Child Adolesc Health. 2019;3(9):605-615. doi:10.1016/S2352-4642(19)30184-1 31324597

[26] Mozolic-Staunton B, Donelly M, Yoxall J, Barbaro J. Early detection for better outcomes: universal developmental surveillance for autism across health and early childhood education settings. Res Autism Spectr Disord. 2020;71:101496. doi:10.1016/j.rasd.2019.101496

[27] Barbaro J, Dissanayake C. Early markers of autism spectrum disorders in infants and toddlers prospectively identified in the Social Attention and Communication Study. Autism. 2013;17(1):64-86. doi:10.1177/1362361312442597 22735682

[28] Barbaro J, Ridgway L, Dissanayake C. Developmental surveillance of infants and toddlers by maternal and child health nurses in an Australian community-based setting: promoting the early identification of autism spectrum disorders. J Pediatr Nurs. 2011;26(4):334-347. doi:10.1016/j.pedn.2010.04.007 21726784

[29] Barbaro J, Dissanayake C. Prospective identification of autism spectrum disorders in infancy and toddlerhood using developmental surveillance: the Social Attention and Communication Study. J Dev Behav Pediatr. 2010;31(5):376-385. doi:10.1097/DBP.0b013e3181df7f3c 20495475

[30] Barbaro J, Dissanayake C, Sadka N, . Universal developmental surveillance for autism in infants, toddlers and preschoolers: the Social Attention and Communication Study–Revised (SACS-R) and SACS-Preschool. Paper presented at: annual meeting of the International Society for Autism Research; May 12, 2018; Rotterdam, Netherlands.

[31] Juffer F, Bakerman-Kranenburg MJ, van Ijzendoorm MH, eds. Promoting Positive Parenting: An Attachment-Based Intervention. Taylor & Francis Group/Lawrence Erlbaum Associates; 2008.

[32] Pickles A, Le Couteur A, Leadbitter K, . Parent-mediated social communication therapy for young children with autism (PACT): long-term follow-up of a randomised controlled trial. Lancet. 2016;388(10059):2501-2509. doi:10.1016/S0140-6736(16)31229-6 27793431 PMC5121131

[33] Bryson SE, Zwaigenbaum L, McDermott C, Rombough V, Brian J. The Autism Observation Scale for Infants: scale development and reliability data. J Autism Dev Disord. 2008;38(4):731-738. doi:10.1007/s10803-007-0440-y 17874180

[34] Lord C, Rutter M, DiLavore PC, Risi S, Gotham K, Bishop S. Autism Diagnostic Observation Schedule: ADOS. 2nd ed. Western Psychological Services; 2012.

[35] Gotham K, Pickles A, Lord C. Standardizing ADOS scores for a measure of severity in autism spectrum disorders. J Autism Dev Disord. 2009;39(5):693-705. doi:10.1007/s10803-008-0674-3 19082876 PMC2922918

[36] Wan MW, Brooks A, Green J, Abel K, Elmadih A. Psychometrics and validation of a brief rating measure of parent-infant interaction: Manchester Assessment of Caregiver-Infant Interaction. Int J Behav Dev. 2017;41(4):542–549. doi:10.1177/0165025416631835

[37] Mullen EM. Mullen Scales of Early Learning. American Guidance Service; 1995.

[38] Sparrow SS, Cicchettim DV, Balla DA. *Vineland Adaptive Behavior Scales, Second Edition (Vineland-II)*. APA PsycTests; 2005.

[39] Fenson L, Dale PS, Reznick JS, et al. Dale PS, Reznick S, Bates E. MacArthur Communicative Development Inventories: User’s Guide and Technical Manual. 1st ed. Singular Publishing Group; 1993.

[40] Gilmore L, Cuskelly M. Factor structure of the Parenting Sense of Competence scale using a normative sample. Child Care Health Dev. 2009;35(1):48-55. doi:10.1111/j.1365-2214.2008.00867.x 18991983

[41] Whitehouse A. Quick files. Center for Open Science; 2021. Accessed August 17, 2021. https://osf.io/qt9gy/quickfiles

[42] Zellner A. An efficient method of estimating seemingly unrelated regressions and tests for aggregation bias. J Am Stat Assoc. 1962;57(298):348-368. doi:10.1080/01621459.1962.10480664

[43] Lu N, Han Y, Chen T, . Power analysis for cross-sectional and longitudinal study designs. Shanghai Arch Psychiatry. 2013;25(4):259-262. 24991165 10.3969/j.issn.1002-0829.2013.04.009 PMC4054560

[44] Mudge JF, Baker LF, Edge CB, Houlahan JE. Setting an optimal α that minimizes errors in null hypothesis significance tests. PLoS One. 2012;7(2):e32734. doi:10.1371/journal.pone.0032734 22389720 PMC3289673

[45] Brian J, Bryson SE, Smith IM, . Stability and change in autism spectrum disorder diagnosis from age 3 to middle childhood in a high-risk sibling cohort. Autism. 2016;20(7):888-892. doi:10.1177/1362361315614979 26685198

[46] Ozonoff S, Young GS, Brian J, . Diagnosis of autism spectrum disorder after age 5 in children evaluated longitudinally since infancy. J Am Acad Child Adolesc Psychiatry. 2018;57(11):849-857. doi:10.1016/j.jaac.2018.06.022 30392626 PMC6235445

