# Supplementary material for: Effect of Preemptive Intervention on Developmental Outcomes Among Infants Showing Early Signs of Autism: A Randomized Clinical Trial of Outcomes to Diagnosis
Source: JAMA Pediatr. 2021 Sep 20;175(11):e213298. doi: 10.1001/jamapediatrics.2021.3298 (PMC8453361; doi:10.1001/jamapediatrics.2021.3298)
Supplement: Supplement 1. — Trial Protocol and Statistical Analysis Plan [file jamapediatr-e213298-s001.pdf]

Effect of pre-emptive intervention on developmental outcomes for infants showing early signs of autism: A randomized clinical trial of outcomes to diagnosis

(Trial Protocol)

Andrew J.O. Whitehouse (Ph.D.),[1,2,3]* Kandice, J. Varcin (Ph.D).,[1,3] Sarah Pillar (B.SpPathHons),[1] Wesley Billingham (B. Sc.)[1], Gail A. Alvares (Ph.D.),[1] Josephine Barbaro (Ph.D.),[4,5] Catherine A. Bent (Ph.D.),[6] Daniel Blenkley (M.Ed.),[7] Maryam Boutrus (Ph.D.),[1,4,8] Abby Chee (MCP),[1] Lacey Chetcuti (Ph.D.),[4,6] Alena Clark (B.Sc.),[1] Emma Davidson (B.Sc.),[9] Stefanie Dimov (MPschSci),[6] Cheryl Dissanayake (Ph.D.),[4,5] Jane Doyle (MCP),[9] Megan Grant (DCP),[6] Cherie C. Green (Ph.D),[6] Megan Harrap (B.Sc.),[1] Teresa Iacono (Ph.D.),[10] Lisa Matys (B.Sc.),[1] Murray Maybery (Ph.D.),[8] Daniel F. Pope (M.Ed.),[7] Michelle Renton (B.Sc.),[1,9] Catherine Rowbottam (B.Sc.),[9] Nancy Sadka (D.Ed.),[5] Leonie Segal (Ph.D.),[11] Vicky Slonims (Ph.D.),[12] Jodie Smith (Ph.D),[6] Carol Taylor (Ph.D.),[7] Scott Wakeling (MCP),[6] Ming Wai Wan (Ph.D.),[7] John Wray (FRACP),[9] Matthew N. Cooper (Ph.D.),[1] Jonathan Green (FRCPsych),[13,14] and Kristelle Hudry (Ph.D.)[4,6]

This supplement contains the following items:

- Original trial protocol, final trial protocol, summary of changes.

[1]CliniKids, Telethon Kids Institute, 15 Hospital Ave, Nedlands, Western Australia 6009, Australia.
[2]The University of Western Australia, 35 Stirling Hwy, Crawley 6009.
[3]School of Allied Health Sciences, Griffith University, Gold Coast, Queensland 4222, Australia.
[4]Cooperative Research Centre for Living with Autism (Autism CRC), Long Pocket, Indooroopilly, Queensland 4068, Australia.
[5]Olga Tennison Autism Research Centre, School of Psychology and Public Health, La Trobe University, Bundoora, Victoria 3086 Australia.
[6]Department of Psychology and Counselling, School of Psychology and Public Health, La Trobe University, Bundoora, Victoria 3086 Australia.
[7]Perinatal Mental Health and Parenting Research Unit (PRIME-RU), Division of Psychology and Mental Health, School of Health Sciences, University of Manchester, Manchester, UK
[8]School of Psychological Science, University of Western Australia, 35 Stirling Hwy, Crawley, Western Australia 6009, Australia.
[9]Child and Adolescent Health Service, Child Development Service, 4-16 Rheola Street, West Perth 6005, Western Australia, Australia.
[10]Living with Disability Research Centre, College of Science, Health and Engineering, PO Box 199 Bendigo 3552, Victoria, Australia.
[12]Australian Centre for Precision Health, Allied Health and Human Movement, University of South Australia, SAHMRI, North Terrace, Adelaide, SA 5000, Australia.
[12]Children's Neurosciences, Evelina London Children's Hospital/IOPPN Kings College London UK.
[13]Division of Neuroscience & Experimental Psychology, School of Biological Sciences, University of Manchester.
[14]Manchester Academic Health Science Centre, Manchester University NHS Foundation Trust, Greater Manchester Mental Health NHS Trust, Manchester, United Kingdom.

*Corresponding author: Prof Andrew Whitehouse, Telethon Kids Institute, University of Western Australia, 100 Roberts Road, Subiaco, Western Australia 6008, Email: Andrew.Whitehouse@telethonkids.org.au, Phone: +61 8 6319 1830.

Original trial protocol

1. TRIAL DETAILS

1.1 Trial details

**Protocol/Clinical Trial Title:**	The Australian Infant Communication and Engagement Study		
**Protocol Number (Version and Date):**	Version 1, 9th June 2016		
**Amendment (Number and Date):**			
**Trial Start Date:**	1st April 2016	**Trial Finish Date:**	31st December 2019
**Coordinating Principal Investigator Name:**	Professor Andrew Whitehouse		
**Coordinating Principal Investigator Contact Details:**	Telethon Kids Institute<br>100 Roberts Rd<br>Subiaco 6008,<br>Western Australia<br>Phone: +61 8 9489 7770<br>Email: Andrew.Whitehouse@telethonkids.org.au		
**Sponsor Name (if applicable):**	Telethon Kids Institute		

1.2 Trial summary

Autism Spectrum Disorder (ASD) is a lifelong developmental disorder affecting more than 1% of people. Social and communication therapies during early childhood are critical for promoting favourable longer-term outcomes in ASD. However, until very recently we have not had interventions tailored towards infants (≤18 months of age) who are at increased risk for ASD. iBASIS-VIPP is a parent-mediated intervention in which therapists use video-feedback to help parents adapt to their infants' interactive styles and promote optimal social and communicative development. We have also shown that the iBASIS-VIPP protocol has preliminary efficacy for improving the developmental outcomes among infants who are at high risk of developing ASD because an older sibling had the condition. The next stage of this research programme is to test the iBASIS-VIPP intervention with infants presenting to a clinical setting with ASD risk-behaviours, such as social and communication delays.

The study design is a two-site (Perth, Melbourne), two-arm ('Treatment as Usual', 'iBASIS'), single-blind (rater) randomized controlled trial (RCT). We will recruit 132 infants (n = 66 at each site) who are between 9 and 16-months of age and showing ASD-risk behaviours (social and/or communication difficulties). Consenting families will be randomized into receiving either the 'iBASIS-VIPP Therapy' (n = 66) or 'Treatment as Usual'. Families in the 'iBASIS-VIPP Therapy' group will receive 10 home-based sessions with a Speech Pathologist or Psychologist

over five months, and undertake 30-minutes daily home practice. Families in the 'Treatment as Usual' group will receive a parent information seminar, which is the current 'best practice' protocol for these infants. Infants in both groups will be re-assessed at follow-up points, time-locked to baseline assessments: (1) 6 months post-baseline (i.e., immediately post 'treatment' period), (2) 12- months post-baseline, and (3) 24-months post-baseline. The primary outcome will be autistic symptom severity immediately post treatment. Secondary outcomes will be a range of social and communicative behaviours relevant to ASD at each of the three follow-up points.

1.3 Abbreviations

Abbreviation	Definition
ADOS-2	Autism Diagnostic Observation Schedule – Second Editon
AOSI	Autism Observation Scale for Infants
ASD	Autism Spectrum Disorder
CDS	Child Development Service, WA Health
CRF	Case report form
DEECD	Department of Education and Early Child Development
GCP	Good Clinical Practice
HREC	Human Research Ethics Committee
iBASIS-VIPP	iBASIS – Video Interaction for promoting Positive Parenting
LTU	La Trobe University
MSEL	Mullen Scales of Early Learning
MACI	Manchester Assessment of Caregiver-Infant Interaction
MCH	Maternal and Child Health, Victoria
OTARC	Olga Tennison Autism Research Centre
PCI	Parent-Child Interaction
PI	Principal Investigator
PICF	Patient Information and Consent Form
PMH	Princess Margaret Hospital, Perth.
RCT	Randomized-controlled trial
SAC	Safety Advisory Committee
SACS-R	Social Attention and Communication Scales-Revised
SAE	Serious adverse effect
TGA	Therapeutic Goods Administration
VABS-II	Vineland Adaptive Behavior Scales – Second edition
WG	MacArthur-Bates Communicative Development Inventory – Words and Gestures form
WS	MacArthur-Bates Communicative Development Inventory – Words and Sentences form

2. RATIONALE AND BACKGROUND

2.1 Summary of previous research

Autism Spectrum Disorder (ASD) is the collective term for developmental disabilities characterized by impairments in social interaction, verbal and nonverbal communication and by repetitive patterns of behaviour.[1] Symptom severity varies but, at a minimum, ASD compromises long-term cognitive and social functioning,[2] with wide-ranging effects on the quality of daily living for the affected individuals[3,4] and their families.[5] Developmental interventions during early childhood are critical for promoting favourable longer-term outcomes in ASD.[6] However, a diagnosis of ASD is currently rarely made before two years of age,[7] which means that many of the best opportunities for therapies to exploit brain plasticity very early in development are not realised.[8]

Until very recently, we have not had the means to identify infants (≤ 18 months old) at high risk of ASD with adequate levels of sensitivity and specificity, nor an intervention tailored to ASD risk behaviours that can be applied to infants so young. There is universal agreement about the urgent need to clinically appraise promising methods for early ASD identification and intervention,[9] in order to enhance the long-term outcomes for the 1 in 88 people (>1%) with ASD.[10]

ASD is generally considered a life-long condition. However, there is accumulating evidence that early and intensive intervention can reduce the severity of the social, communication and adaptive disabilities associated with ASD. Theoretical articles have focused particularly on the first two years of life as a potentially critical period for intervention, during which neural plasticity is heightened and therapies may have maximal long-term benefits.[8] Two of the major logistical challenges in achieving this important research goal have been highlighted by the 2013 update of the Interagency Autism Coordinating Council's Strategic Plan.[9] These are a lack of:

(1) Valid methods for identifying infants at very high-risk for a later diagnosis of ASD; and
(2) Interventions tailored to very-high risk infants that have efficacy for reducing disability.

Our collective research team has taken an internationally leading role in addressing this major public health challenge, and Figure 1 outlines our strategic approach.

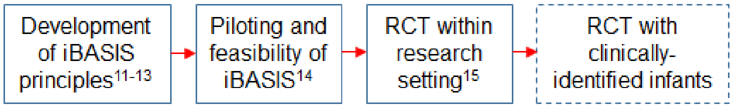

Supplementary Figure 1. Our over-arching research programme. This project (dotted line) in relation to completed research (solid, line) and future research (dashed).

Through a series of collaborative research projects, our CI team has: (1) developed a theoretical foundation for iBASIS-VIPP therapy,[11-13] (2) piloted the feasibility and acceptability of iBASIS-VIPP via a case series,[14] and (3) demonstrated preliminary efficacy of iBASIS-VIPP via an RCT with infant siblings of children with ASD ('familial high-risk' infants)[15]. The proposed study is the culmination of this long-term research programme: a full-scale RCT to determine the efficacy of iBASIS-VIPP with 'community high risk' infants.

Theoretical foundations and evidence for parent-mediated interventions

Genetic variation is known to play a major role in the aetiology of ASD.[16-17] However, there is emerging evidence that any risk susceptibility in brain and behavioural functioning caused by genetic factors may be exacerbated by poor-quality interactions within the social environment.[18] The 'interactive specialisation' theory of developmental neuroscience proposes that the quality of an infant's early social interactions has a major influence on the developing brain structures that underpin social behaviour.[19] Parent-infant interactions are critical in creating the optimal social environment that facilitates the development of neural pathways within the social brain system.[8] Parental interaction styles that are less directive and more sensitive to child cues are known to assist in the development of early social skills, and are associated with more favourable long-term communicative and social outcomes for children with typical[19] or atypical[20] development.

Infants are typically born with biases to orient towards, attend to, and learn from social stimuli.[21] By contrast, there is good evidence that infants later diagnosed with ASD have reduced or impaired function in one or more of the underlying biasing mechanisms early in life.[22-23] Our team has found evidence that this disruption in social orienting among infants later diagnosed with ASD can lead to differences in parent-infant interaction styles. Parents of high-risk infants often exhibit less sensitivity to their infant's behavioural cues and increased directiveness (e.g. behavioural prompting).[11-13] While these poor interactional cycles are not the primary cause of the child's ASD, they may maintain or amplify a pre-existing biological vulnerability to ASD in the infant.

Three small studies have provided evidence that optimising parent-infant interactions within the first year of life can enhance developmental outcomes for infants at high risk of ASD.[24-26] However, each of these small-scale studies is at 'proof of concept' stage only, has included sample sizes of fewer than 10 infants, and was not a randomised test. Currently the most advanced line of research for infants at high-risk of ASD is an intervention developed by CIs Green and Slonims, iBASIS-VIPP, which has demonstrated proof-of concept[14] and preliminary efficacy in a small-scale RCT (pilot data).[15]

2.2 The 'iBASIS-VIPP' intervention

iBASIS-VIPP is a modification of the Video Interaction for promoting Positive Parenting program,[27] which uses video-feedback to help parents adapt to their infants' communication styles and promote optimal social and communicative development. In a series of home-based sessions, the therapist films parent-infant interactions and uses footage excerpts to improve parental understanding of the infant's communicative signals. Within an initial case-series of 8 infant siblings of children with ASD (aged 8-10 months) and their parents, CIs Green, Slonims and AI Wan demonstrated the feasibility of conducting iBASIS-VIPP and its acceptability by families.[14] In January 2015, CIs Green and Slonims and AIs Wan and Taylor published a pilot efficacy RCT of iBASIS-VIPP intervention with a sample of 54 'familial high risk' infants.[15] These pilot data are described below.

Pilot data[15]*:* A two-arm, assessor-blinded RCT was conducted with 'familial high risk' infants (aged 7-10 months) randomised to receive iBASIS-VIPP (n = 28) or no intervention (n = 26). Families in the iBASIS-VIPP arm received 10 therapy sessions over 5-months. Outcome assessments were conducted immediately post-treatment (M age: 14-months). Figure 2 shows proximal intervention effects on the parent-infant interaction measure (MACI) and distal effects on language ability (MSEL), social behaviour (VABS-II) and ASD risk behaviours (AOSI; see Table 1 for descriptions of tests). Though 95% confidence intervals (CI) sometimes include the null, the iBASIS-VIPP treatment group demonstrated significant improvement in parental non-directiveness and trends toward reduced ASD risk behaviours and improved infant social behaviours. There were no significant effects on language.

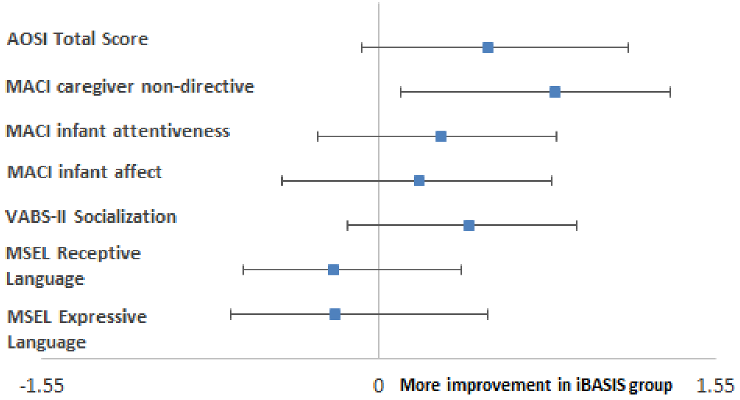

Supplementary Figure 2. Mean effect size (95%CI) of iBASIS-VIPP treatment vs 'no intervention'.[15]

The proposed study: Full-scale RCT of iBASIS-VIPP

Our pilot data provide the first RCT evidence that a parent-mediated intervention may be efficacious in optimising development for infants at high risk for ASD, positioning the iBASIS-VIPP model for evaluation within a full-scale clinical trial. The proposed RCT will be the gold-standard test of the highly promising iBASIS-VIPP therapy, and build on these pilot data by:

1. Recruiting infants through community clinics. The 'familial high-risk' design of the pilot RCT provided an efficient means of recruiting infants for whom there was increased risk for ASD. However, recurrence data indicate that only a minority of infant siblings (~20%) are expected to be on a developmental trajectory toward ASD outcome.[17] This makes it challenging to draw conclusions from the pilot RCT design about the efficacy of iBASIS-VIPP in mitigating clinical levels of ASD symptoms. To achieve this aim, it is essential to test iBASIS-VIPP with infants who are identified through community clinics as displaying early behavioural risk factors for ASD.

2. Recruiting a sufficiently large sample of infants that will provide statistical power to confidently estimate effect sizes of iBASIS-VIPP efficacy. Given the substantial phenotypic and aetiological heterogeneity of ASD,[28] our CI team has argued that failure to account for individual differences in response to therapy may obscure real treatment effects in subpopulations.[28-30] The 'interactive specialisation' theory of developmental neuroscience proposes that iBASIS-VIPP efficacy will be dependent upon the quality of parent interaction behaviour. We have previously found that indices of parental sensitivity and directiveness (MACI) are key predictors of treatment effects in parent-mediated ASD therapies.[12,31] An adequate test of this hypothesis with iBASIS-VIPP (Secondary Aim) requires a large sample size with sufficient statistical power to identify potentially small effects.

The RCT will take place with participant referral through community health services in WA (Child Development Service, CDS) and Victoria (Department of Education and Early Child Development, DEECD). We will recruit infants referred to either of these services who are: (1) 9-16-months of age, and (2) showing ASD risk-behaviours (social and/or communication difficulties). Consenting families will be randomized into receiving either the 'iBASIS-VIPP Therapy' (n = 66) or 'Treatment as Usual' (n = 66). Families in the 'iBASIS-VIPP Therapy' group will receive 10 home-based sessions with a Speech Pathologist or Psychologist over five months, and undertake 30-minutes daily home practice. Families in the 'Current best practice' group will receive current treatment protocol offered within the community which may comprise a parent information workshop (2 hours long) regarding early interactions with their infant, the provision of reading materials on infant development, or developmental monitoring.

Significance

The significance of this research is its potential to establish efficacy for a very early intervention that may mitigate long-term ASD-related disability. A reduction of the disability associated with ASD will not only lead to greater participation in society of these individuals, but also a decrease in the long-term health-care costs. ASD affects more than 1% of the population, with estimated annual support costs to Australia of up to $7 billion.[32] A recent report found that the improved long-term outcomes associated with early behavioural intervention for ASD (commencing at 36 months of age) reduces the support costs to society by approximately $1.6m over a lifetime.[33] Our hypothesis is that commencing a targeted intervention in the first 18 months of life would reduce disability even further, leading to greater benefits to the individual, their family and society. This project will be critical to Australian health policy more broadly, given the imminent establishment of the National Disability Insurance Scheme, and its role in funding evidence-based interventions that may reduce future support needs.

3. TRIAL AIMS AND HYPOTHESES

3.1 Aims and hypotheses

This study will test a parent-mediated, behavioural intervention (iBASIS-VIPP) with 9-16-month old infants identified as showing 'risk behaviours' for Autism Spectrum Disorder. In this RCT, we aim to determine whether iBASIS-VIPP applied for a 5-month period can reduce ASD symptom severity immediately post-treatment (6-months post-baseline) and at 12- and 24-months post baseline compared to 'treatment as usual'.

We hypothesize that, compared to 'treatment as usual', iBASIS-VIPP therapy will:

1. Reduce the primary outcome of ASD symptom severity immediately post-treatment (6-months post-baseline) and the secondary outcome of ASD symptom severity 12- and 24-months post-baseline.
2. Improve the tertiary outcomes of infant social attentiveness and communication and language skills immediately post-treatment (15-22 months of age) and at 12- and 24-months post-baseline (21-28 months and 33-40 months of age, respectively).

4. TRIAL DESIGN

4.1 Primary, secondary and tertiary outcomes

Infants will receive three follow-up assessments, time-locked to the baseline assessment:

1. *Follow-up assessment 1*: Immediately following treatment completion (6-months post-baseline), when infants are between 15 and 22 months of age (depending upon age at trial entry);
2. *Follow-up assessment 2*: Six-months following treatment completion (12-months post baseline), when infants are between 21 and 28 months of age (depending upon age at trial entry).
3. *Follow-up assessment 3:* Eighteen-months following treatment completion (24-months post-baseline), when infants are between 33 and 40 months of age (depending upon age at trial entry).

The Primary Outcome measure is the Total Score of the AOSI at Follow-up Assessment 1. The Secondary Outcomes are the Total Scores of the ADOS-2 at Follow-up Assessments 2 and 3. The Tertiary Outcomes are all variables generated by the psychometric assessments at Follow-up Assessments 1, 2 and 3 (MACI, MSEL, VABS-II, MCDI scores).

4.2 Type of trial

This Phase 2 trial is a two-site (Perth, Melbourne), single-blind (rater) RCT, in which participants will be randomly allocated to receive iBASIS-VIPP therapy or 'treatment as usual' over a period of 5 months. There will be three follow-up assessments: immediate post-treatment (6-months post-baseline), and 12- and 24-months post-baseline. These assessments will be conducted by the Research Assistant within two weeks of these timepoints. Figure 2 outlines the trial procedure.

4.3 Number of participants

We seek to recruit 132 infants between the ages of 9 and 16 months of age, who will be randomised to two groups (n = 66 in each group).

4.4 Protection against other sources of bias

Baseline assessment will be undertaken prior to parents being informed of treatment assignment. Following baseline assessment, participants' details will be sent to the randomisation site (Telethon Kids Institute) for assignment to either the Treatment as Usual or iBASIS-VIPP groups. Group allocation of participants will be by minimization method, stratified by child gender, site (Perth, Melbourne), score on the Social Attention and Communication Scale–Revised (SACS-R) 12-month checklist (i.e., 3, 4, or 5)[34] and age band at baseline appointment (9-11 months/12-14 months). Researchers will be housed separately from staff involved in therapy and will attend separate meetings. Research interviews will be constructed so as to avoid inadvertent divulging of information that could infer treatment status. The behavioural measure (AOSI) is rated by videotape blind to case details and treatment status. A random 20% of AOSI assessments and 15% of MACI videos will be double-coded by a blinded expert. The assessment suite and materials used will be quite different in type and location to that used for the treatment intervention avoiding any familiarity effect for children in the treatment arm. Bias due to therapist effects will be minimised by frequent check on continuing therapist fidelity. All

treatment sessions are videotaped. 5% of these sessions will be scrutinised by independent clinicians against fidelity criteria in the treatment.

4.5 Maintenance of blinding records.

Trial randomisation codes will be generated by the trial coordinator based at the Telethon Kids Institute, Dr Kandice Varcin, who will hold a copy of the codes and their association with group. Once a group has been allocated to a participant, its corresponding code will be recorded in the participants' electronic and hardcopy file. The code will be held by Dr Varcin, who is not involved in testing any participants. These codes will be held in password-protected electronic database on a password protected server at the Telethon Kids Institute.

4.6 Method of tracking implantable devices

Not applicable.

4.7 Description of interventions

Infants will be randomised into two groups:

Treatment as Usual

Families in the "treatment as usual' group will receive current treatment protocol offered within the community, which comprises a 'parent information workshop' and reading material. Parents will be invited to attend an information workshop conducted by an experienced Speech Pathologist, where they will be provided with information about how to best promote their infant's early social and communication skills. The workshop will last approximately two hours, and parents will be given reading material to take home after the workshop. Parents will not be prevented from accessing other therapies. Within the 'Treatment as Usual' group, we will quantify all contact with health professionals by asking parents to complete a monthly diary. We will also qualify the nature of this contact by directly contacting the health professional(s) involved.

iBASIS-VIPP intervention

The manualised iBASIS-VIPP intervention will be delivered in family homes by a Research Therapist (Speech Pathologist/Psychologist). One parent will be asked to participate in all of the therapy sessions. They will receive 10 sessions over five months, and will undertake 30-mins daily home practice. During iBASIS-VIPP therapy sessions, parent and infant are videotaped during daily interactions.[14-15] Video feedback provides the opportunity to focus the caregiver's attention on the infant's communicative signals and expressions, thereby stimulating skills for observing and empathising with the child. Core methods include:[27] (1) a focus on the communicative aspects of the particular parent-infant dyad; (2) viewing 'successful' excerpts from videotaped interactions, providing positive examples of sensitive parenting; and (3) involvement of a trained therapist to frame observations to assist self-reflection, and to focus behavioural change. Intervention content focusses on enhancing parental observation, attributing communicative intent to infant behaviours that may be difficult to interpret, and facilitating responses that will build infant interaction. To this foundation, we have added components that have been tested in our previous ASD intervention studies,[35] focusing on promoting early social communication skills, such as joint attention and turn-taking. As with the Treatment as Usual

group, we will also quantify and qualify any contact the families have with other health professionals.

4.8 Accountability procedures

The first three months of the project will be spent providing the Research Therapist with comprehensive training to fidelity in iBASIS-VIPP. Our training protocol will follow a 'cascade model', which has worked extremely well in existing collaborations between Prof Green and collaborators in India. Ms Taylor (therapy supervisor for the pilot RCT)[15] will provide initial training to the Research Therapists and a 'Research Champion. The Research Champion will provide day-to-day guidance to the Research Therapists, including fortnightly supervision sessions. Monthly video/teleconferences will be held between the Research Therapists, the Research Champions and Ms Taylor to monitor progress. CI Taylor will independently rate 5% of trial therapy tapes to test within-trial fidelity.

4.9 Expected duration of trial and participant participation.

The study will involve a recruitment telephone call (conducted by CDS staff), an eligibility screening telephone call (Research Therapist), a baseline assessment (Research Assistant), a treatment phase (Research Therapist) and three follow-up assessments (Research Assistant). At any time throughout the duration of the trial, participants will be able to contact the Trial Coordinator (Kandice Varcin) to discuss any aspect of the trial. The project timetable and milestones are presented in Table 1.

Supplementary Table 1. Project timetable and milestones

Milestone	Month
1. Study commences with ethics approval already obtained	1
2. MACI training in Manchester, UK.	1
3. iBASIS-VIPP Training meeting in Manchester, UK	2
4. Therapist training requirements met	3
5. Participant recruitment commences	4
6. Therapy commences for recruited participants	4
7. 1st follow-up assessments (6-months post-baseline) commence	9
8. 66 patients recruited and randomised	14
9. 2nd follow-up assessments (12-months post baseline) commence	15
10. All therapy sessions completed	25
11. All post-treatment assessment sessions completed	26
12. 3rd follow-up assessments (24-months post-baseline) commence	27
13. Participant recruitment concludes	27
14. 1st follow-up assessments (6-months post-baseline) conclude	30
15. Data analysis and write-up for 1st follow-up session (6-months post-baseline)	30
16. 2nd follow-up assessments (12-months post-baseline) conclude	36
17. Data analysis and write-up for 2nd follow-up session (12-months post-treatment)	36
18. 3rd follow-up assessments (24-months post baseline) conclude	48
19. Data analysis and write-up for 3rd follow-up session (24-months post-baseline)	48

Participant recruitment and eligibility screening

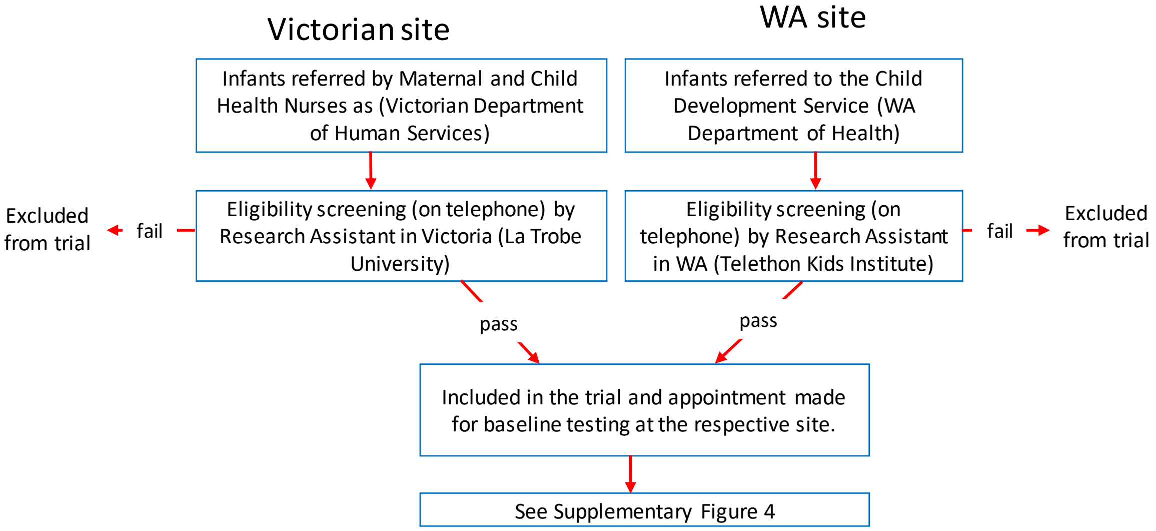

Supplementary Figure 3. Recruitment procedure at the Melbourne and Perth sites

At the WA site, staff at each of the 11 CDS 'intake' sites around the Perth metropolitan area will identify newly referred infants who may be eligible for the current study. The caregiver of any infant meeting the study criteria will be telephoned by a CDS staff member who will inform the families about the trial. The contact details of any consenting families will be sent immediately to the Research Assistant at the Telethon Kids Institute. The Research Assistant will then telephone the caregiver to conduct the eligibility screening, including verbal administration of the SACS-R.[34] For those who express an interest in participating, an appointment will be made to attend the Telethon Kids Institute for a Baseline Assessment.

At the Melbourne site, potential infant participants will be identified through Maternal and Child Health (MCH) nurse, who will perform SACS-R developmental screens on infants at 12-months of age.[34] Any infant who fails the standard screen for developmental delay used by the nurses will be informed about this study. Caregivers interested in having their infant participate in the trial will have their contact details sent immediately to the Research Assistant at La Trobe University. The Research Assistant will then telephone the caregiver by telephone to conduct the eligibility screening including repeat verbal administration of the SACS-R.[34] For those who express an interest in participating, an appointment will be made to attend La Trobe University for a Baseline Assessment.

Once an appointment has been made, the caregivers will be mailed an appointment confirmation letter, along with a Participant Information and Consent Form (PICF). The PICF contains a description of the project, information about random assignment to the trial conditions, procedures and time commitment, confidentiality and privacy, and contact information for the researchers and the PMH and La Trobe University Ethics Committees. It also contains a statement that participants are free to withdraw from the study at any time and that participation is voluntary. These forms have been created in accordance with Good Clinical Practice

guidelines. The parent(s)/caregiver(s) will asked to read the PICF, and encouraged to ask the research team any questions they may have at the Baseline appointment. The PICF includes the contact details of the study team, and Caregivers will be able to contact the study team prior to the Baseline appointment should they want to.

Baseline Assessment

Baseline assessments for will occur within four weeks of eligibility screening, and will be conducted by a Research Assistant based at each site. At the beginning of this session, the Research Assistant will ask the caregiver whether (a) they have read the PICF previously mailed to them, and (b) whether they had any questions. Research Assistants will also provide a document outlining the study stages in more detail, and talk caregivers through this document.

After all questions have been answered to the satisfaction of the caregiver, s/he will be asked to sign the consent forms and will be provided with an information sheet to keep. If not, they will be referred on to appropriate services. Upon consent, participants will be given the baseline assessment, which includes a suite of behavioural tests. See Supplementary Figure 4 for the trial procedure.

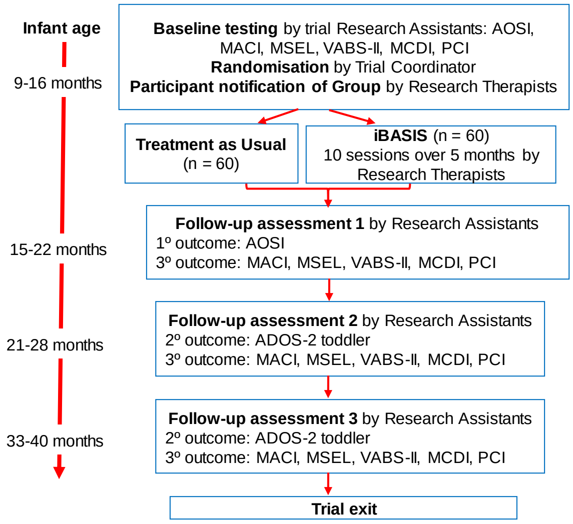

Supplementary Figure 4. Trial procedure

Randomisation

Following the baseline session, the Research Assistant will communicate to the Trial Coordinator (based in Perth) that the session has been completed and consent has been obtained. The Trial Coordinator will then conduct the randomisation and communicate the family's group to the Research Therapist at the relevant site.

Participant notification of group

The Research Therapist at each site will contact the participant to arrange for either the first iBASIS-VIPP therapy session ('iBASIS-VIPP' group), or current best practice ('Treatment as Usual' group).

'Treatment phase'

For participants allocated to the treatment group, iBASIS-VIPP therapy sessions will be conducted by a Research Therapist at each site and commence within two weeks of Baseline assessment. Families will receive 10 sessions at fortnightly intervals over five months, and will undertake 30-mins daily home practice (described in 4.6). Caregivers in the 'treatment as usual' group may be invited to attend a parent information workshop that provides information about early interactions with infants, and/or be provided with reading materials on infant development, or offered any other services within the community that may be considered appropriate for the infant.

Follow-up assessment 1 (6-months post-baseline assessment)

Follow-up assessment 1 will be conducted by the trial Research Assistant at each site and will take place within two weeks of the final day of the 5-month treatment phase. Our preference is for these assessments to take place in a quiet clinic room at either the Telethon Kids Institute or La Trobe University. However, if this is not possible, the assessment will take place at the participants' homes. The assessment will comprise a suite of behavioural assessments as outlined in Supplementary Tables 2 and 3.

Follow-up assessment 2 (12-months post-baseline assessment)

Follow-up assessment 2 will be conducted by the trial Research Assistant at each site and will take place within two weeks of the 12-month anniversary of the infant completing the baseline assessment. Our preference is for these assessments to take place in a quiet clinic room at either the Telethon Kids Institute or La Trobe University. However, if this is not possible, the assessment will take place at the participants' homes. The assessment will comprise a suite of behavioural assessments as outlined in Supplementary Tables 2 and 3.

Follow-up assessment 3 (24-months post-baseline assessment)

Follow-up assessment 3 will be conducted by the trial Research Assistant at each site and will take place within two weeks of the 24 month anniversary of the infant completing the baseline assessment. Our preference is for these assessments to take place in a quiet clinic room at either the Telethon Kids Institute or La Trobe University. However, if this is not possible, the assessment will take place at the participants' homes. The assessment will comprise a suite of behavioural assessments as outlined in Supplementary Tables 2 and 3.

Supplementary Table 2. Schedule of assessments.

	Telephone screening	Baseline	Follow-up assessment 1	Follow-up assessment 2	Follow-up assessment 3
**Basic information**					
Study month commencing	1	2	8	14	26
Infants' age (months)	9-16	9-16	15-22	21-28	33-40
**Eligibility**					
SACS-R	X				
Inclusion/exclusion criteria	X				
Infant medical history	X				
Informed Consent		X			
**Questionnaires**					
Family questionnaire		X	X	X	X
MCDI		X	X	X	X
VABS-II		X	X	X	X
**Face-to-face assessment**					
AOSI		X	X		
ADOS-2			X	X	X

PCI		X	X	X	X
MSEL		X	X	X	X
Clinical Best Estimate Diagnosis					X

Supplementary Table 3. Psychometric tests delivered at the baseline and follow-up assessments by a Research Assistant who is blind to the infant's group status.

**Name**	**Details**
Autism Observation Scale for Infants (AOSI)[36]	**Description:** Semi-structured assessment developed specifically to elicit early behavioural risk factors of ASD in infants aged 6-18 months.<br>**Procedure**: Administered by researchers trained to protocol fidelity, and scored from videotape by researchers with demonstrated coding reliability (>80% agreement) who are blind to group allocation.<br>Scoring: Individual item scores (range 0-2) for 18 behaviours summed to produce AOSI Total Score (range 0-50; higher scores = more ASD symptoms).<br>**Validity:** Higher mean number of behavioural 'markers' at 12 and 18 months in infants with later ASD vs. no ASD[12,37]; acceptable test-retest (≥ .61) and inter-rater reliability (≥ .92)[36] for AOSI Total Score.
Autism Diagnostic Observation Schedule – Second edition (ADOS-2): Toddler Module[37]	**Description:** Semi-structured assessment developed to elicit behaviours relevant to an ASD diagnosis in toddlers 12-30 months (Toddler Module) or aged 31 months and above (Module 1 and 2).<br>**Procedure:** Administered by researchers trained to protocol fidelity, and scored from videotape by researchers with demonstrated coding reliability (>80% agreement) who are blind to group allocation.<br>**Scores:** Continuous symptom measure: Total Algorithm Score (range 0-28; higher = more symptoms). Binary diagnostic classification: meets/does not meet established cut-off for ASD (i.e., non-verbal toddlers ≥ 12; verbal ≥ 10).<br>**Validity:** Continuous measure: Excellent test-retest and inter-rater agreement (Intra-class correlations ≥ .90). Binary classification: Excellent sensitivity & specificity of cut-off scores for identifying ASD vs. other disabilities (≥. 81).[38]
Parent Child Interaction (PCI)	**Description:** Parent-infant free-play interaction sample. Filmed for 10mins, with standard set of age-appropriate toys provided.<br>**Procedure:** Footage coded by blinded trained researchers using Manchester-Assessment of Caregiver-Infant Interaction (MACI) procedures. Subset (10%) of footage scored by Dr Ming-Wae Wan for inter-rater reliability.<br>**Scoring:** Footage coded on 7-point Likert-type scales (higher scores = better quality interaction) for parent behaviour (sensitive responsiveness, non-directiveness), infant behaviour (affect, liveliness, attentiveness to parent), and dyadic interaction (engagement intensity, mutuality).<br>**Validity:** Low scores on MACI infant scales predict ASD diagnosis at 3 years.[12,13]
Mullen Scales of Early Learning (MSEL)[39]	**Description:** Standardised developmental assessment norm-referenced 0 - 68 months.<br>**Procedure:** Trained, blinded assessors administer tasks directly with infants.<br>**Scoring:** Standardised t-scores (M = 50; SD = 10) and age equivalents computed for each sub-scale (higher scores = better developed skills).

	**Validity:** Excellent test-retest and inter-scorer reliability for ages ≤ 24 months (r ≥ .82).[36] Infants at high risk of ASD score consistently poorly.[40-41]
Vineland Adaptive Behavior Scales – 2nd edition (VABS-II)[42]	**Description:** Caregiver-report measure of functional skills, norm-referenced 0 - 90 years. Communication and Socialization domains relevant to current trial. **Procedure:** Parent completes form (i.e., not blind to group allocation) which is scored up by researcher according to manualised procedures. **Scoring:** Domain Standard Scores (M = 100; SD = 15), and subdomain v-scores (M = 15; SD = 3) and age-equivalents computed (higher scores = better skills). **Validity:** Excellent test-retest and inter-rater reliability for ages ≤ 24 months (r ≥ .81)[41] and evidence of low scores in 'familial high risk' infants.[18,30]
MacArthur Communicative Development Inventory (MCDI)[43-44]	**Description and Procedure:** Caregiver-report checklists tallying Receptive and Expressive Vocabulary knowledge in infants aged 8-30 months. Words and Gestures (WG) form used at Baseline and F1 and Words and Sentences (WS) form used at Follow-up 2 and 3. **Scoring:** Endorsed items summed to yield raw Receptive and Expressive vocabulary counts (possible range 0-680; higher score = more language skill). **Validity:** Very strong internal consistency (r ≥ .95)[44]
Clinical Best Estimate Diagnosis	**Description:** Clinicians experienced in ASD diagnosis will review data collected and provide an appraisal of ASD diagnostic outcome, similar to previous trials.[35]

4.10 Criteria for termination of the trial

Discontinuation criteria for the trial reflect guidelines set out by the TGA. The trial will be terminated in the case that support for the trial is withdrawn from the HREC or sponsor. This includes the following possibilities:

- Evidence of significant deviation from the trial protocol that may threaten wellbeing of participants;
- Evidence that allowing the trial to continue carries an unacceptable risk of death, serious injury or illness to participants;
- Evidence that one treatment proves to be so much better or worse that to continue would disadvantage one group of participants;
- Evidence that conduct of the trial is in breach of the law.

In regards to risk to participants, in the unlikely event of an SAE, a review would be conducted involving the SAC and the HREC board in relation to the future continuation of the trial. If the SAE is definitely due to trial participation, it may result in discontinuation of the whole trial (recruitment and treatment), depending on the decision made by the SAC and HREC based on available evidence concerning risk to participants. If the SAE is found to be possibly or probably due to trial participation, it will result in the affected participant/s immediately ceasing involvement in the trial. The SAC will then evaluate how the overall trial should proceed.

4.11 Identification of source data

In this trial, the following documents will be considered source data:

- Hard copies of the following caregiver-report questionnaires:
  - Demographics Information
  - MCDI

    - VABS-II
- Hard copies of the following protocols completed by Research Assistants:
    - AOSI
    - ADOS-2
    - MSEL
    - Clinical best estimate diagnosis
- Hard copies of the following Research Therapist reports:
    - Fidelity rating scale
- Digital video footage of the following:
    - AOSI assessment
    - ADOS-2 assessment
    - PCI samples
    - In-home iBASIS-VIPP therapy sessions

5. SOURCE AND SELECTION OF PARTICIPANTS

5.1 Source and feasibility of participant recruitment

Infants will be recruited from two sources. At the WA site, staff at each of the 11 CDS 'intake' sites around the Perth metropolitan area will identify newly referred infants who may be eligible for the current study. The caregiver of any infant meeting the study criteria will be telephoned by a CDS staff member who will inform the families about the trial. The contact details of any consenting families will be sent immediately to the Research Assistant at the Telethon Kids Institute. The Research Assistant will then telephone the caregiver to conduct the eligibility screening, including verbal administration of the SACS-R[34] For those who express an interest in participating, an appointment will be made to attend the Telethon Kids Institute for a Baseline Assessment.

At the Melbourne site, potential infant participants will be identified through MCH nurses, who will perform developmental screens on infants at 12-months of age. Any infant who fails the standard screen for developmental delay used by the nurses will be informed about this study. Caregivers interested in having their infant participate in the trial will have their contact details sent immediately to the Research Assistant at La Trobe University. The Research Assistant will then telephone the caregiver by telephone to conduct the eligibility screening, including verbal administration of the SACS-R. For those who express an interest in participating, an appointment will be made to attend La Trobe University for a Baseline Assessment.

5.2 Participant inclusion criteria

Infants will be offered participation in this trial if they:

(a) Are between 9 and 16 months of age;
(b) Endorsement of three or more of the 'key items' on the 12-month old SACS-R[34] (absent/atypical pointing, waving, imitation, eye contact, response to name).
(c) The primary caregiver involved in the trial speaks sufficient English to: (i) understand the requirements of the study, and (ii) is deemed to be able to participate fully in the therapy sessions.

5.3 Participant exclusion criteria

Participants will be excluded from the study if they meet either of the following characteristics:

(a) The infant has a diagnosed comorbidity (or significant clinical indicators of a comorbidity) known to affect neurological and developmental abilities (e.g., preterm birth < 32 weeks, cerebral palsy, Down syndrome, Rett syndrome, other chromosomal abnormality, hearing/visual impairment), and/or

(b) The family does not intend to remain living in the Perth/Melbourne areas for the next two years.

5.4 Participant withdrawal criteria

A premature discontinuation will occur when a participant for whom informed consent was obtained ceases participation in the study, regardless of circumstances, before the completion of the study. If a participant must be prematurely discontinued from the study, the primary reason for discontinuation must be recorded as one of the following:

- Failure to meet inclusion/exclusion criteria;
- The participant's home is deemed by the Trial Coordinator to not provide a safe and healthy working environment for the Research Therapist.
- SAE linked to iBASIS-VIPP;
  - In the unlikely event that the participant's psychological state deteriorates as a result of treatment they would be withdrawn from the study and alternative therapy or referral offered.
- Protocol deviation/violation, including lack of compliance;
- An infant participant received a diagnosis with a condition that meets 'exclusion (a)'. In these cases, we will ensure that the participant has received an appropriate health and medical referral for their diagnosis (e.g., GP, paediatrician, audiologist etc.) at the time of their withdrawal from the study.
- Voluntary withdrawal of consent (reasons will be documented);
- Lost to follow-up (every effort will be made to contact the patient first; a certified letter will be sent).

Data collection for withdrawn subjects:

All enrolled participants who prematurely discontinue from the study regardless of cause will be asked to come in for a final evaluation. A final evaluation is defined as completion of the evaluations scheduled for the final post-treatment assessment (Follow-up assessment 3). Parents/caregivers refusing to have their infant assessed must be requested in writing to come in for a visit. A copy of the letter will be kept by the PI together with the source documentation. The reasons for premature discontinuation from the study will be reflected on the Study Termination Record of the CRF.

Patient replacement procedures

Patients in this study who prematurely discontinue from the study will not be replaced.

6. TREATMENT OF PARTICIPANTS

6.1 Description and justification for the treatments

Information concerning the theory and development of the behavioural intervention is detailed in the 'Rationale and Background' section, and is summarised here.

Name:
(1) iBASIS-VIPP
(2) Treatment as usual

Treatment schedule:
Participants in the 'iBASIS-VIPP' group will receive 10 therapy sessions over a period of 5 months. Parents will be asked to undertake 30 minutes home practice with their infant daily. The 'Treatment as Usual' group will receive current treatment protocol offered within community health services, which varies considerably and may include a referral to a developmental paediatrician, a wait-list period (2-3 months), invitation to parent information sessions, and speech pathology assessment and intervention.

6.2 . The medications/interventions permitted during the trial.

At the outset of the study, parents/carers will be asked to report all medications the child is taking, including the reason for taking the medication and duration for which medication has been taken to date. Participation in this trial does not prevent participants' seeking behavioural therapy outside of the trial framework. The nature and quality of any outside therapy and medications received will be recorded at the Follow-up Assessments.

6.3 Procedure for monitoring participant compliance

Compliance in the 'iBASIS-VIPP group' will be monitored via a compliance checklist completed by the Research Therapist at each face-to-face session, and by the parent/caregiver following each daily practice session.

7. ASSESSMENT OF EFFICACY

7.1 Specification of efficacy parameters and methods and timing for assessing and recording

Unless otherwise specified, all efficacy parameters will be recorded in source format. This will then be entered into an electronic database where it will be stored alongside participant's ID numbers. The database will be regularly checked for accuracy, and source documents will be retained to clarify any issues. Further information on the primary, secondary and tertiary outcome measures is provided in Supplementary Table 3.

Primary outcome measure:
Autism Observation Scale for Infants will be used to measure the severity of autistic symptoms.
Method of Assessment: Researcher-administered semi-structured play assessment with infant. Scored from video by researcher with demonstrated high level reliability, who is blind to treatment group.

Timing of assessment. Follow-up Assessment 1.

Secondary outcome measure:
The Toddler Module of the Autism Diagnostic Observation Schedule – Second Edition will be used to measure the severity of autistic symptoms.

Method of Assessment: Researcher-administered semi-structured play assessment with toddler. Scored from video by researcher with demonstrated high level reliability, who is blind to treatment group.
Timing of assessment. Follow-up Assessment 2 and 3.

Tertiary outcome measures:
The Manchester Assessment of Caregiver-Infant Interaction will be used to measure the quality pf parent-infant interactions.

Method of Assessment: Parent-Infant free play interaction sample.
Scored from video by researcher with demonstrated high level reliability, who is blind to treatment group.
Timing of assessment. Follow-up assessments 1, 2 and 3.

The Mullen Scales of Early Learning will be used to assessed cognitive and motor ability

Method of Assessment: Researcher-administered standardised developmental assessment.
Scored live by researcher administering the assessment.
Timing of assessment. Follow-up assessments 1, 2 and 3.

The Vineland Adaptive Behavior Scales – 2nd edition will be used to assess daily functional skills.

Method of Assessment: Caregiver-report questionnaire
Scored by researcher.
Timing of assessment. Follow-up assessments 1, 2 and 3.

The MacArthur-Bates Communicative Development Inventory will be used to assess early language development.

Method of Assessment: Caregiver-report questionnaire
Scored by researcher.
Timing of assessment. Follow-up assessments 1 (WG form), and 2 and 3 (WS form).

Clinical ASD diagnostic outcome will be assessed by experienced independent clinicians.

Method of Assessment: Independent clinician review of collected data.
Scored by independent clinicians
Timing of assessment. Follow-up assessment 3.

7.2 Methods for analysing efficacy parameters

This is detailed in section 'Statistical analysis plan'.

8 ASSESSMENT OF SAFETY

8.1 Summary of known and potential risks and benefits, if any, to research participants.

iBASIS-VIPP is a behavioural intervention that supports parents to interact with their infant to maximise social and communication development. The potential benefit of this behavioural intervention is a reduction in autistic symptomatology, and an increase in developmental abilities, such as language and social skills. There are no known risks of this intervention, and our previous studies have shown that this therapy is well tolerated by families. The safety issues for this trial are negligible.

8.2 The safety parameters and method for assessing and recording safety parameters.

Safety parameters
Key safety parameters in the trial are occurrence of side effects, type of side effects, occurrence of adverse events, type of adverse event, occurrence and type of intercurrent illnesses across the trial.

Method for assessing and recording
Parents/infants in the iBASIS-VIPP group will have face-to-face therapy sessions 10 times throughout the 5-month treatment period. Parents will also be asked to complete 30 minutes practice each day. At the end of each therapy session, the Research Therapist will record if there have been any adverse effects of the therapy noted during the session, as well as during the period since the last testing session. This will be recorded on the Case Report Form completed by the Research Therapist.

8.3 Details of the Data and Safety Monitoring Board, or equivalent.

An independent Data and Safety Monitoring Committee (DSMC) has been assembled to monitor the progress, safety and efficacy of this clinical trial and provide critical evaluation and recommendations to the study investigators and all sponsors of this trial. The members of the DSMC serve in an individual 'expert' capacity. The DSMC will meet every 6 months throughout this trial and review cumulative study data to evaluate study conduct, the scientific validity and data integrity of the study including safety of this trial. Scheduled meetings will be supplemented with additional meetings or conference calls scheduled as needed.

8.4 The procedures for eliciting reports of and for recording and reporting adverse events. Include definitions of adverse events.

We define an adverse event as any untoward medical occurrence in a patient or clinical investigation subject associated with the experimental treatment. The treatment protocol is focused solely on teaching parents how to modify their interactions with their infants, and therefore we believe the risk of any adverse events is extremely low. Caregivers will be provided with monitoring forms at the outset of the study to record any side effects they believe may be associated with the treatment. These forms will provide space to record side effects daily if needed.

Any adverse event will be reported to the PMH and LTU Human Research Ethics Committees in a detailed, formal written report, which will identify participants involved by study code rather than name or address. These reports will include an assessment of the severity and causality of

the adverse event in relation to the treatment protocol (see Table 4), as well as detailed information regarding the steps taken by the Study Team with regard to both the treatment protocol and the participant involved.

Supplementary Table 4. The assignment of causality for each AE should be made by the investigator responsible for the care of the participant based on the definitions in the table below.

**Relationship**	**Description**
**Unrelated**	Sufficient information exists to indicate that the aetiology is unrelated to the treatment protocol.
**Unlikely**	When there is no reasonable temporal association between the treatment protocol and the suspected adverse event. The event could have been related to the patient's clinical state or concomitant treatment(s).
**Possible**	There is some evidence to suggest a causal relationship (e.g. because the event occurs within a reasonable time after administration of the treatment session). However, the influence of other factors may have contributed to the event (e.g. the participant's clinical condition, other concomitant treatments).
**Definitely**	There is clear evidence to suggest a causal relationship, based on: temporal relationship to the treatment session
**Not assessable**	When causality is, for one reason or another not accessible, e.g. because of insufficient evidence, conflicting data or poor documentation

8.5 The type and duration of the follow-up of participants after adverse events.

Adverse events will be monitored by the Research Therapists at each iBASIS session, which take place at fortnightly intervals during the 5-month treatment period. If an adverse event is reported by the caregiver, infant wellbeing will be followed up by study medical staff through phone calls and visits as needed (depending on the nature of the adverse event). Participants will be made aware that they are able to contact study staff regarding the adverse event during this period, as well as receiving contact from study medical staff. Any adverse event reported at the Perth site will be immediately communicated through Professor Whitehouse to the CDS Director (Ms Emma Davidson) and the CDS Senior Clinical Advisor (Clinical Associate Professor John Wray).

Any parent who yields a score above the 'clinical cutoff' on any of the DASS scales[46] (Depression $\geq$ 14, Anxiety $\geq$ 10, Stress $\geq$ 17) will be contacted by a study researcher with clinical training (Speech Pathologist, Clinical/Developmental Psychologist or medical doctor) and informed about their high score. The study researcher will encourage the parent to make an appointment with their GP to discuss the implications of this score.

9. DATA MANAGEMENT, STATISTICAL ANALYSIS AND RECORD KEEPING

9.1 Description of the statistical methods to be employed, including timing of any planned interim analysis.

Data will be analyzed via ‘intention to treat’ by the study biostatistician. Baseline data will be examined to determine the success of randomization, comparing group mean scores on the AOSI, MACI, MSEL, VABS-II and MCDI through separate independent-samples t-tests. Lack of statistical significance for each comparison at a = .2 will imply successful randomization. Should this not be achieved, the analysis techniques described below will be modified to adjust for baseline differences.

The primary outcome will be AOSI Total Score at Follow-up Assessment 1 (infant ages: 15-20 months). Our power analyses are based on our pilot study.[15] A sample of 66 per group provides a two-sided independent-samples t-test 85% power to detect a 0.52 SD change in AOSI Total Score from baseline to follow-up (a = .05). This represents a group difference in change scores of between 2.46 (for SD = 4.67) and 3.70 (for SD = 6.98) points. We have argued previously that a change in AOSI scores of two points or more is clinically significant, and this trial is adequately powered to detect this difference.

The secondary outcomes will be ADOS-2 Total Algorithm Score at Follow-up assessments 2 and 3. Using a two-tailed independent samples t-test with 66 participants in each group, we will have 79% power to detect a mean difference between groups of 2.5 points on the ADOS-2, with the assumption of the variability being 46% of the mean (a = .05). This a difference between a mean (SD) of 12.5 (5.7) in the Treatment as Usual Group and 10 (4.6) in the iBASIS Group (Cohen’s d = 0.48).

Tertiary outcome variables – MACI, MSEL, VABS-II, MCDI scores – will be assessed as change scores (baseline to each follow-up assessment) within an analysis of covariance framework, adjusting for baseline scores. An additional independent variable of interest in the ‘Treatment as Usual’ group will be the number of ‘contact hours’ each family has had with health professionals. Each type of service (e.g., counseling, speech pathology etc.) will be weighted and summed into a continuous measure. We will examine the distribution of this variable and determine whether a mathematic adjustment or categorization is required prior to analysis.

9.2 The number of participants planned to be enrolled

We will recruit 132 infants into this trial (n = 66 in each group), which has been chosen for reason of statistical power, detailed in ‘AICES analysis plan’.

9.3 The level of significance to be used.

The alpha level will be set at $p < .05$

9.4 Procedures for reporting any deviation(s) from the original statistical plan,

Any deviations from the original statistical plan will be described and justified in the protocol.

9.5 The selection of participants to be included in the analyses

Data will be analyzed through an ‘intention to treat’ approach and analyses will include all randomized participants.

9.6 Information on how data will be managed, including coding for computer analysis and data handling (collection, storage, maintenance, security and archiving). Include details regarding these processes if the data is sent off-site (e.g. encryption).

Upon entry into the study, participants will be allocated a unique ID, and all data will be referenced to this code. Data collection will be conducted only by authorised members of the study team who are named on the HREC application for the trial.

For the duration of the trial, source data from Perth and Melbourne will be stored at the respective sites. At the Perth site, baseline and follow-up assessments will be kept in a locked filing cabinet in Prof Whitehouse's office at Telethon Kids Institute. Source data for the iBASIS-VIPP therapy protocol will be stored in a locked filing cabinet at the West Perth Child Development Service, which is where the Research Therapist will be based. At the Melbourne site, baseline and follow-up assessments will be kept in a locked filing cabinet at the Olga Tennison Autism Research Centre (OTARC), LTU. Source data for the iBASIS therapy protocol will be stored in a locked filing cabinet in Dr Hudry's office at LTU.

As the trial progresses, Research Assistants and Therapists will enter source data through a web portal into an electronic database on an online, secure, password-protected cloud computing database. All study data for analysis purposes will be entered into REDCap (which stands for Research Electronic Data Cpature), while at the Melbourne site, participant information will be stored in Salesforce.com Customer Relationship Management (CRM) system for scheduling purposes. Both REDCap and Salesforce databases are only accessible by authorised personnel, including researchers and administrative support staff. Salesforce.com CRM security and privacy credentials and policies can be found at https://trust.salesforce.com/trust/ and http://www.salesforce.com/au/company/privacy/, respectively. REDCap is a server-based software so can only be installed on a local web server by Institutional IT staff who then allows access to study personnel through a web browser. Only sufficiently trained and supervised research assistants will be hired to enter data. An audit of data files over time to ensure completeness of data collection will be conducted on a routine basis. Video footage of recorded as part of the study protocols will be labelled with the ID the infants/caregivers and be kept on a secure server at the Telethon Kids Institute. At the conclusion of the trial, all source data will be transferred to the Telethon Kids Institute for storage in a locked-filing cabinet in Prof Whitehouse's office. Source data will be kept for a minimum of 15 years from the completion of the trial. At the Perth site, hard copies of data recorded at the baseline assessment, therapy sessions (if received), and first follow-up assessment will also be provided to the CDS treating clinician at the conclusion of the latter assessment.

The biological samples will be labelled with a de-identified ID. However, as this study constitutes clinical and genetic information, it is critical to have the ability to re-identify participants. Three people only will have access to the "key" document that contains both participant IDs and contact details (including name, date of birth, phone numbers, email and postal address). Andrew Whitehouse and Kristelle Hudry will hold an electronic copy of the key document on a password protected document server at their respective institutions (Telethon Kids Institute and La Trobe University). The Tissue Bank manager at the Wesley Research Institute, Emma Raymond, will also hold a copy of consent forms and have access to a 'key' document matching names and ID codes. This will be stored on a secure server at the Wesley

Research Institute in order to comply with Tissue Bank policies for long-term data retention. Approval to access these 'key' documents will be governed by the Data Access Committee.

9.7 Procedure for accounting for missing, unused, and spurious (*false*) data.

Analyses of data involving missing measures will be undertaken wherever possible either by maximum-likelihood estimation or by the use of multiple imputation, carried out using the iterative chained equation approach as implemented in Stata. Both approaches allow for potentially selective but non-informative attrition.

10 MONITORING

10.1 Statement that the trial investigators/institutions will permit trial-related monitoring, audits, and regulatory inspections, providing direct access to source data/documents.

The investigators and institution will permit trial-related monitoring, audits, and regulatory inspections, providing direct access to source documents, as required. Trial monitoring may be undertaken by the HREC, sponsor, and TGA. This will be stipulated in the Participant Information Statement.

11 QUALITY CONTROL AND QUALITY ASSURANCE

11.1 Statement that the trial will be conducted in compliance with the protocol, Good Clinical Practice and the application regulatory requirements.

The trial will be conducted in compliance with the protocol, Good Clinical Practice and the relevant regulatory requirements.

11.2 Quality control & quality assurance measures to ensure quality of data.

The first three months of the project will be spent providing the Research Therapist with comprehensive training to fidelity in iBASIS-VIPP. Our training protocol will follow a 'cascade model', which has worked extremely well in existing collaborations between PI Green and collaborators in India. Ms Taylor (therapy supervisor for the pilot RCT)[15] will provide initial training to the Research Therapists and up to two 'Research Champions'. The Research Champions will be based in Perth and provide day-to-day guidance to the Research Therapists, including fortnightly supervision sessions. Monthly video/teleconferences will be held between the Research Therapists, the Research Champions and Ms Taylor to monitor progress. Ms Taylor will independently rate 5% of trial therapy tapes to test within-trial fidelity.

Any changes to the Clinical Trial Protocol will be documented and separate versions kept on file. Our trial will also be recorded in an online clinical trial database, as is standard practice for the conduct of clinical trials.

12 ETHICS

12.1 Description of ethical considerations with particular reference to participant consent

This application will be reviewed and fully approved in writing by the PMH Ethics Committee. A number of potential ethical issues that may arise in the course of the trial have been identified and discussed at length in the HREC application for this trial.

Informed consent:
The study will recruit infants aged between 9 and 16 months, who are showing behavioural ‘warning signs’ of ASD. These infants are unable to provide informed consent. We address this issue by their parent/guardian/carer provide informed consent on their behalf.

Uneven relationships.
At the Perth site, some infants who are offered participation in this study may be patients of a study researcher, A/Prof John Wray. On these occasions, Dr Wray will emphasise to caregivers that they are not obliged to take part in the study and that they will not compromise their relationship with him as a clinician if they choose not to participate. All participant information statements will clearly emphasise that participation in this study is voluntary, there will be no reimbursement for participation, and participants are free to withdraw at any time, as well as highlighting possible risks and benefits of participation.

Behavioural assessments:
Infants will undergo several behavioural assessments, each lasting no more than 2.5 hours, which will take place at either the Telethon Kids Institute (Perth site) or La Trobe University (Melbourne site). This poses no risk to the children. The clinical examination will be undertaken by experienced research assistants who will make a judgment on whether any child experiences undue levels of distress. If so, the clinical examination will cease

Storage of personal information:
Parents will complete a questionnaire asking about the medical and social history of the child and family. Personal information relating to research participants, themselves, and/ or their parents/ or child/ren will be stored on an online, secure, password-protected cloud computing database, known as Salesforce.com Customer Relationship Management (CRM) system and is only accessible by authorised personnel, including researchers and administrative support staff. Salesforce.com CRM security and privacy credentials and policies can be found at https://trust.salesforce.com/trust/ and http://www.salesforce.com/au/company/privacy/, respectively.

Video footage will also be recorded and stored as part of the intervention and assessment protocols. Some of this information could be considered personal. Parents will provide written consent for the collection of this information. Upon recruitment participants will be allocated a code number. Source data (including digital files of video footage), as well as electronic copies of source data will be labelled with their code number only. The sole electronic file linking personal details with these code numbers will be stored on a password protected server at the TKI and LTU. There will be no hard copy of this document.

Limited disclosure of ASD-related hypotheses

While the aim of this RCT is to determine whether a novel behavioural therapy can mitigate the onset of ASD behaviours in later childhood, the Participant Information and Consent Form does not prominently feature the term 'autism' or Autism Spectrum Disorder'.

This decision was based on the following reasoning:

1. The infants recruited into this trial will have been flagged as having early social and communication difficulties (not ASD). Our previous studies have found that these infants are at increased risk of ASD. However: (a) the majority of these infants (~60%) will not develop ASD, and (b) there is currently no reliable way to predict which infants will and won't develop ASD.
2. Our considerable clinical and research experience has found that even mentioning 'autism' or 'ASD', even in terms of 'increased risk for ASD' can cause family significant distress.
3. Weighing up points #1 and #2, we feel that it is most ethical to discuss their infants' developmental problems in terms of 'language and communication difficulties' and not 'increased ASD risk'.

It is possible that families may link their infant's difficulties with ASD through the course of this study. For example, many of the Investigators have conducted previous research into ASD, and families may google this information. To allay concerns, we have included a specific item in the Participant Information and Consent Form:

"Please do not be concerned that your child may have more serious developmental concerns, such as autism. It is not possible to know whether an infant younger than 18 months of age has autism. The vast majority of children who have early social and communication difficulties do not grow up to have autism."

13 BUDGET, FINANCING, INDEMNITY AND INSURANCE.

13.1 Budget, financing, indemnity and insurance, if not addressed in a separate agreement.

This project is funded by grants from the Telethon-Perth Children's Hospital grant, the Autism Cooperative Research Centre, the Understanding Disease RFA at LTU, and the Angela Wright Bennett Foundation.

This clinical trial will be undertaken with adequate insurance/indemnity to cover the liability of the investigators and sponsor. In the unlikely case of injury suffered by participants as a result of their participation in the study, participants will be referred to their General Practitioner, who will assist them in arranging appropriate medical treatment. Participants will also be informed that they may have a right to take legal action to obtain compensation for any injuries or complications resulting from the study. Compensation may be available if the participants' injury or complication is sufficiently serious and is caused by unsafe drugs or equipment, or by the negligence of one of the parties involved in the study. If they receive compensation that includes an amount for medical expenses, they will be required to pay for their child's medical treatment from those compensation monies. If the participant is not eligible for compensation for their

injury or complication under the law, but are eligible for Medicare, then they can receive any medical treatment required for their injury or complication free of charge as a public patient in any Australian public hospital. These guidelines will be outlined on the participant information statement.

14 PUBLICATION

14.1 Publication and dissemination of trial results (including any limitations).

In accordance with the guidelines stipulated by the funding bodies, the results of this study will be disseminated as widely as possible into the scientific and broader community. This will include publication in peer-reviewed journals, scholarly book chapters, presentation at conferences, publication in conference proceedings and student theses. Publications arising from this project will be deposited into an open access institutional repository, where possible. Results will also be disseminated into the wider community in a format appropriate for laypeople, through links including our website and Facebook pages, as well as newsletters and in presentations.

Final trial protocol

1. TRIAL DETAILS

1.2 Trial details

**Protocol/Clinical Trial Title:**	The Australian Infant Communication and Engagement Study		
**Protocol Number (Version and Date):**	Version 3, 6th August 2018		
**Amendment (Number and Date):**			
**Trial Start Date:**	1st April 2016	**Trial Finish Date:**	31st December 2019
**Coordinating Principal Investigator Name:**	Professor Andrew Whitehouse		
**Coordinating Principal Investigator Contact Details:**	Telethon Kids Institute<br>100 Roberts Rd<br>Subiaco 6008,<br>Western Australia<br>Phone: +61 8 9489 7770<br>Email: Andrew.Whitehouse@telethonkids.org.au		
**Sponsor Name (if applicable):**	Telethon Kids Institute		

1.2 Trial summary

Autism Spectrum Disorder (ASD) is a lifelong developmental disorder affecting more than 1% of people. Social and communication therapies during early childhood are critical for promoting favourable longer-term outcomes in ASD. However, until very recently we have not had interventions tailored towards infants (≤18 months of age) who are at increased risk for ASD. iBASIS-VIPP is a parent-mediated intervention in which therapists use video-feedback to help parents adapt to their infants' interactive styles and promote optimal social and communicative development. We have also shown that the iBASIS-VIPP protocol has preliminary efficacy for improving the developmental outcomes among infants who are at high risk of developing ASD because an older sibling had the condition. The next stage of this research programme is to test the iBASIS-VIPP intervention with infants presenting to a clinical setting with ASD risk-behaviours, such as social and communication delays.

The study design is a two-site (Perth, Melbourne), two-arm ('Treatment as Usual', 'iBASIS'), single-blind (rater) randomized controlled trial (RCT). We will recruit 132 infants (n = 66 at each site) who are between 9 and 16-months of age and showing ASD-risk behaviours (social and/or communication difficulties). Consenting families will be randomized into receiving either the 'iBASIS-VIPP Therapy' (n = 66) or 'Treatment as Usual'. Families in the 'iBASIS-VIPP Therapy' group will receive 10 home-based sessions with a Speech Pathologist or Psychologist

over five months, and undertake 30-minutes daily home practice. Families in the 'Treatment as Usual' group will receive current treatment protocol offered within the community which may comprise a parent information seminar, the provision of reading materials on infant development, or developmental monitoring.. Infants in both groups will be re-assessed at follow-up points, time-locked to baseline assessments: (1) 6 months post-baseline (i.e., immediately post 'treatment' period), (2) 12- months post-baseline, and (3) 24-months post-baseline. Analyses will be split into two phases: The first analysis phase will examine infant ASD symptom severity (primary outcome) and developmental outcomes (secondary outcomes) immediately post treatment. The second analysis phase will examine infant ASD symptom severity (primary outcome) and developmental outcomes (secondary outcomes) when examined across three follow-up timepoints up to 2 years post-baseline.

1.3 Abbreviations

Abbreviation	Definition
ADOS-2	Autism Diagnostic Observation Schedule – Second Editon
AOSI	Autism Observation Scale for Infants
ASD	Autism Spectrum Disorder
CDS	Child Development Service, WA Health
CRF	Case report form
DEECD	Department of Education and Early Child Development
GCP	Good Clinical Practice
HREC	Human Research Ethics Committee
iBASIS-VIPP	iBASIS – Video Interaction for promoting Positive Parenting
LTU	La Trobe University
MSEL	Mullen Scales of Early Learning
MACI	Manchester Assessment of Caregiver-Infant Interaction
MCH	Maternal and Child Health, Victoria
OTARC	Olga Tennison Autism Research Centre
PCI	Parent-Child Interaction
PSOC	Parenting Sense of Competence scale
PI	Principal Investigator
PICF	Patient Information and Consent Form
PMH	Princess Margaret Hospital, Perth.
RCT	Randomized-controlled trial
SAC	Safety Advisory Committee
SACS-R	Social Attention and Communication Scales-Revised
SAE	Serious adverse effect
TGA	Therapeutic Goods Administration
VABS-II	Vineland Adaptive Behavior Scales – Second edition
WG	MacArthur-Bates Communicative Development Inventory – Words and Gestures form
WS	MacArthur-Bates Communicative Development Inventory – Words and Sentences form

2. RATIONALE AND BACKGROUND

2.1 Summary of previous research

Autism Spectrum Disorder (ASD) is the collective term for developmental disabilities characterized by impairments in social interaction, verbal and nonverbal communication and by repetitive patterns of behaviour.[1] Symptom severity varies but, at a minimum, ASD compromises long-term cognitive and social functioning,[2] with wide-ranging effects on the quality of daily living for the affected individuals[3,4] and their families.[5] Developmental interventions during early childhood are critical for promoting favourable longer-term outcomes in ASD.[6] However, a diagnosis of ASD is currently rarely made before two years of age,[7] which means that many of the best opportunities for therapies to exploit brain plasticity very early in development are not realised.[8]

Until very recently, we have not had the means to identify infants (≤ 18 months old) at high risk of ASD with adequate levels of sensitivity and specificity, nor an intervention tailored to ASD risk behaviours that can be applied to infants so young. There is universal agreement about the urgent need to clinically appraise promising methods for early ASD identification and intervention,[9] in order to enhance the long-term outcomes for the 1 in 88 people (>1%) with ASD.[10]

ASD is generally considered a life-long condition. However, there is accumulating evidence that early and intensive intervention can reduce the severity of the social, communication and adaptive disabilities associated with ASD. Theoretical articles have focused particularly on the first two years of life as a potentially critical period for intervention, during which neural plasticity is heightened and therapies may have maximal long-term benefits.[8] Two of the major logistical challenges in achieving this important research goal have been highlighted by the 2013 update of the Interagency Autism Coordinating Council's Strategic Plan.[9] These are a lack of:

(1) Valid methods for identifying infants at very high-risk for a later diagnosis of ASD; and
(2) Interventions tailored to very-high risk infants that have efficacy for reducing disability.

Our collective research team has taken an internationally leading role in addressing this major public health challenge, and Figure 1 outlines our strategic approach.

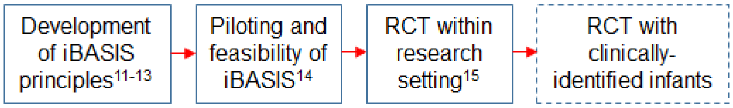

Supplementary Figure 1. Our over-arching research programme. This project (dotted line) in relation to completed research (solid, line) and future research (dashed).

Through a series of collaborative research projects, our CI team has: (1) developed a theoretical foundation for iBASIS-VIPP therapy,[11-13] (2) piloted the feasibility and acceptability of iBASIS-VIPP via a case series,[14] and (3) demonstrated preliminary efficacy of iBASIS-VIPP via an RCT with infant siblings of children with ASD ('familial high-risk' infants)[15]. The proposed study is the culmination of this long-term research programme: a full-scale RCT to determine the efficacy of iBASIS-VIPP with 'community high risk' infants.

Theoretical foundations and evidence for parent-mediated interventions

Genetic variation is known to play a major role in the aetiology of ASD.[16-17] However, there is emerging evidence that any risk susceptibility in brain and behavioural functioning caused by genetic factors may be exacerbated by poor-quality interactions within the social environment.[18] The ‘interactive specialisation’ theory of developmental neuroscience proposes that the quality of an infant’s early social interactions has a major influence on the developing brain structures that underpin social behaviour.[19] Parent-infant interactions are critical in creating the optimal social environment that facilitates the development of neural pathways within the social brain system.[8] Parental interaction styles that are less directive and more sensitive to child cues are known to assist in the development of early social skills, and are associated with more favourable long-term communicative and social outcomes for children with typical[19] or atypical[20] development.

Infants are typically born with biases to orient towards, attend to, and learn from social stimuli.[21] By contrast, there is good evidence that infants later diagnosed with ASD have reduced or impaired function in one or more of the underlying biasing mechanisms early in life.[22-23] Our team has found evidence that this disruption in social orienting among infants later diagnosed with ASD can lead to differences in parent-infant interaction styles. Parents of high-risk infants often exhibit less sensitivity to their infant’s behavioural cues and increased directiveness (e.g. behavioural prompting).[11-13] While these poor interactional cycles are not the primary cause of the child’s ASD, they may maintain or amplify a pre-existing biological vulnerability to ASD in the infant.

Three small studies have provided evidence that optimising parent-infant interactions within the first year of life can enhance developmental outcomes for infants at high risk of ASD.[24-26] However, each of these small-scale studies is at ‘proof of concept’ stage only, has included sample sizes of fewer than 10 infants, and was not a randomised test. Currently the most advanced line of research for infants at high-risk of ASD is an intervention developed by CIs Green and Slonims, iBASIS-VIPP, which has demonstrated proof-of concept[14] and preliminary efficacy in a small-scale RCT (pilot data).[15]

2.2 The ‘iBASIS-VIPP’ intervention

iBASIS-VIPP is a modification of the Video Interaction for promoting Positive Parenting program,[27] which uses video-feedback to help parents adapt to their infants’ communication styles and promote optimal social and communicative development. In a series of home-based sessions, the therapist films parent-infant interactions and uses footage excerpts to improve parental understanding of the infant’s communicative signals. Within an initial case-series of 8 infant siblings of children with ASD (aged 8-10 months) and their parents, CIs Green, Slonims and AI Wan demonstrated the feasibility of conducting iBASIS-VIPP and its acceptability by families.[14] In January 2015, CIs Green and Slonims and AIs Wan and Taylor published a pilot efficacy RCT of iBASIS-VIPP intervention with a sample of 54 ‘familial high risk’ infants.[15] These pilot data are described below.

Pilot data[15]*:* A two-arm, assessor-blinded RCT was conducted with 'familial high risk' infants (aged 7-10 months) randomised to receive iBASIS-VIPP (n = 28) or no intervention (n = 26). Families in the iBASIS-VIPP arm received 10 therapy sessions over 5-months. Outcome assessments were conducted immediately post-treatment (M age: 14-months). Figure 2 shows proximal intervention effects on the parent-infant interaction measure (MACI) and distal effects on language ability (MSEL), social behaviour (VABS-II) and ASD risk behaviours (AOSI; see Table 1 for descriptions of tests). Though 95% confidence intervals (CI) sometimes include the null, the iBASIS-VIPP treatment group demonstrated significant improvement in parental non-directiveness and trends toward reduced ASD risk behaviours and improved infant social behaviours. There were no significant effects on language.

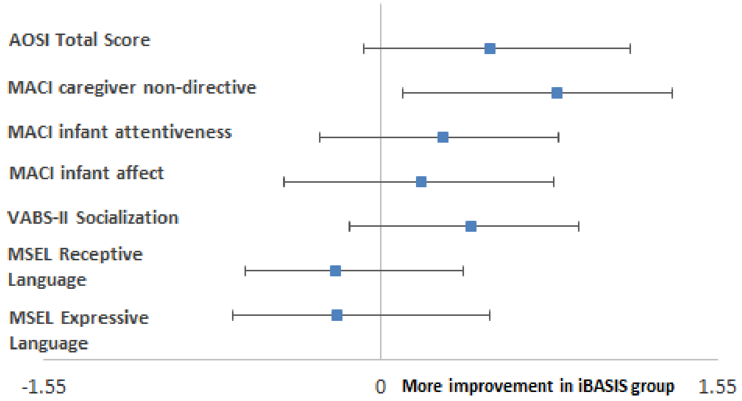

Supplementary Figure 2. Mean effect size (95%CI) of iBASIS-VIPP treatment vs 'no intervention'.[15]

The proposed study: Full-scale RCT of iBASIS-VIPP

Our pilot data provide the first RCT evidence that a parent-mediated intervention may be efficacious in optimising development for infants at high risk for ASD, positioning the iBASIS-VIPP model for evaluation within a full-scale clinical trial. The proposed RCT will be the gold-standard test of the highly promising iBASIS-VIPP therapy, and build on these pilot data by:

1. Recruiting infants through community clinics. The 'familial high-risk' design of the pilot RCT provided an efficient means of recruiting infants for whom there was increased risk for ASD. However, recurrence data indicate that only a minority of infant siblings (~20%) are expected to be on a developmental trajectory toward ASD outcome.[17] This makes it challenging to draw conclusions from the pilot RCT design about the efficacy of iBASIS-VIPP in mitigating clinical levels of ASD symptoms. To achieve this aim, it is essential to test iBASIS-VIPP with infants who are identified through community clinics as displaying early behavioural risk factors for ASD.

2. Recruiting a sufficiently large sample of infants that will provide statistical power to confidently estimate effect sizes of iBASIS-VIPP efficacy. Given the substantial phenotypic and aetiological heterogeneity of ASD,[28] our CI team has argued that failure to account for individual differences in response to therapy may obscure real treatment effects in subpopulations.[28-30] The 'interactive specialisation' theory of developmental neuroscience proposes that iBASIS-VIPP efficacy will be dependent upon the quality of parent interaction behaviour. We have previously found that indices of parental sensitivity and directiveness (MACI) are key predictors of treatment effects in parent-mediated ASD therapies.[12,31] An adequate test of this hypothesis with iBASIS-VIPP (Secondary Aim) requires a large sample size with sufficient statistical power to identify potentially small effects.

The RCT will take place with participant referral through community health services in WA (Child Development Service, CDS) and Victoria (Department of Education and Early Child Development, DEECD). We will recruit infants referred to either of these services who are: (1) 9-16-months of age, and (2) showing ASD risk-behaviours (social and/or communication difficulties). Consenting families will be randomized into receiving either the 'iBASIS-VIPP Therapy' (n = 66) or 'Treatment as Usual' (n = 66). Families in the 'iBASIS-VIPP Therapy' group will receive 10 home-based sessions with a Speech Pathologist or Psychologist over five months, and undertake 30-minutes daily home practice. Families in the 'Current best practice' group will receive current treatment protocol offered within the community which may comprise a parent information workshop (2 hours long) regarding early interactions with their infant, the provision of reading materials on infant development, or developmental monitoring.

Significance

The significance of this research is its potential to establish efficacy for a very early intervention that may mitigate long-term ASD-related disability. A reduction of the disability associated with ASD will not only lead to greater participation in society of these individuals, but also a decrease in the long-term health-care costs. ASD affects more than 1% of the population, with estimated annual support costs to Australia of up to $7 billion.[32] A recent report found that the improved long-term outcomes associated with early behavioural intervention for ASD (commencing at 36 months of age) reduces the support costs to society by approximately $1.6m over a lifetime.[33] Our hypothesis is that commencing a targeted intervention in the first 18 months of life would reduce disability even further, leading to greater benefits to the individual, their family and society. This project will be critical to Australian health policy more broadly, given the imminent establishment of the National Disability Insurance Scheme, and its role in funding evidence-based interventions that may reduce future support needs.

3. TRIAL AIMS AND HYPOTHESES

3.1 Aims and hypotheses

This study will test a parent-mediated, behavioural intervention (iBASIS-VIPP) with 9-16-month old infants identified as showing 'risk behaviours' for Autism Spectrum Disorder. In this RCT, we aim to determine whether iBASIS-VIPP applied for a 5-month period can reduce ASD symptom severity immediately post-treatment (6-months post-baseline) and at 12- and 24-months post baseline compared to 'treatment as usual'.

Analyses will be split into two phases. In the first phase, we hypothesize that, compared to 'treatment as usual', iBASIS-VIPP therapy will:

1. Reduce the primary outcome of ASD symptom severity; and
2. Improve the secondary outcome of developmental outcomes immediately post-treatment (6-months post-baseline).

In the second analysis phase, we hypothesise that, compared to 'treatment as usual', iBASIS-VIPP therapy will:

1. Reduce the primary outcome of ASD symptom severity when examined across three follow-up timepoints (immediate post treatment, 12-months post-baseline, 24-months post-baseline) up to 2 years post-baseline;
2. Improve the secondary outcome of developmental outcomes when examined across three follow-up timepoints (immediate post treatment, 12-months post-baseline, 24-months post-baseline) up to 2 years post-baseline.

4. TRIAL DESIGN

4.1 Primary and secondary outcomes

Infants will receive three follow-up assessments, time-locked to the baseline assessment:

4. *Follow-up assessment 1*: Immediately following treatment completion (6-months post-baseline), when infants are between 15 and 22 months of age (depending upon age at trial entry);
5. *Follow-up assessment 2*: Six-months following treatment completion (12-months post baseline), when infants are between 21 and 28 months of age (depending upon age at trial entry).
6. *Follow-up assessment 3:* Eighteen-months following treatment completion (24-months post-baseline), when infants are between 33 and 40 months of age (depending upon age at trial entry).

The Primary Outcome measure is ASD symptom severity at immediate post-treatment outcome (first analysis phase) and ASD symptom severity across the three follow-up time points (second analysis phase). The Secondary Outcomes are developmental outcomes at immediate treatment end point (first analysis phase), and developmental outcomes across the three follow-up time points (second analysis phase).

4.2 Type of trial

This Phase 2 trial is a two-site (Perth, Melbourne), single-blind (rater) RCT, in which participants will be randomly allocated to receive iBASIS-VIPP therapy or 'treatment as usual' over a period of 5 months. There will be three follow-up assessments: immediate post-treatment (6-months post-baseline), and 12- and 24-months post-baseline. These assessments will be conducted by the Research Assistant within two weeks of these timepoints. Figure 2 outlines the trial procedure.

4.7 Number of participants

We seek to recruit 132 infants between the ages of 9 and 16 months of age, who will be randomised to two groups (n = 66 in each group).

4.8 Protection against other sources of bias

Baseline assessment will be undertaken prior to parents being informed of treatment assignment. Following baseline assessment, participants' details will be sent to the randomisation site (Telethon Kids Institute) for assignment to either the Treatment as Usual or iBASIS-VIPP groups. Group allocation of participants will be by minimization method, stratified by child gender, site (Perth, Melbourne), score on the Social Attention and Communication Scale–

Revised (SACS-R) 12-month checklist (i.e., 3, 4, or 5)[34] and age band at eligibility screen (9-11 months/12-14 months). Researchers will be housed separately from staff involved in therapy and will attend separate meetings. Research interviews will be constructed so as to avoid inadvertent divulging of information that could infer treatment status. The behavioural measure (AOSI) is rated by videotape blind to case details and treatment status. A random 20% of AOSI assessments and 15% of MACI videos will be double-coded by a blinded expert. The assessment suite and materials used will be quite different in type and location to that used for the treatment intervention avoiding any familiarity effect for children in the treatment arm. Bias due to therapist effects will be minimised by frequent check on continuing therapist fidelity. All treatment sessions are videotaped. 5% of these sessions will be scrutinised by independent clinicians against fidelity criteria in the treatment.

4.9 Maintenance of blinding records.

Trial randomisation codes will be generated by the trial coordinator based at the Telethon Kids Institute, Dr Kandice Varcin, who will hold a copy of the codes and their association with group. Once a group has been allocated to a participant, its corresponding code will be recorded in the participants' electronic and hardcopy file. The code will be held by Dr Varcin, who is not involved in testing any participants. These codes will be held in password-protected electronic database on a password protected server at the Telethon Kids Institute.

4.10 Method of tracking implantable devices

Not applicable.

4.7 Description of interventions

Infants will be randomised into two groups:

Treatment as Usual

Families in the 'treatment as usual' group will receive current treatment protocol offered within the community, which may comprise a 'parent information workshop', reading material, or developmental monitoring. Parents will not be prevented from accessing other therapies. Within the 'Treatment as Usual' group, we will quantify all contact with health professionals by asking parents to complete a monthly diary. We will also qualify the nature of this contact by directly contacting the health professional(s) involved. We have used this protocol in our previous RCTs and successfully incorporated these data in our statistical analyses.[35]

iBASIS-VIPP intervention

The manualised iBASIS-VIPP intervention will be delivered in family homes by a Research Therapist (Speech Pathologist/Psychologist). One parent will be asked to participate in all of the therapy sessions. They will receive 10 sessions over five months, and will undertake 30-mins daily home practice. During iBASIS-VIPP therapy sessions, parent and infant are videotaped during daily interactions.[14-15] Video feedback provides the opportunity to focus the caregiver's attention on the infant's communicative signals and expressions, thereby stimulating skills for observing and empathising with the child. Core methods include:[27] (1) a focus on the communicative aspects of the particular parent-infant dyad; (2) viewing 'successful' excerpts from videotaped interactions, providing positive examples of sensitive parenting; and (3) involvement of a trained therapist to frame observations to assist self-reflection, and to focus

behavioural change. Intervention content focusses on enhancing parental observation, attributing communicative intent to infant behaviours that may be difficult to interpret, and facilitating responses that will build infant interaction. To this foundation, we have added components that have been tested in our previous ASD intervention studies,[35] focusing on promoting early social communication skills, such as joint attention and turn-taking. As with the Treatment as Usual group, we will also quantify and qualify any contact the families have with other health professionals.

4.8 Accountability procedures

The first three months of the project will be spent providing the Research Therapist with comprehensive training to fidelity in iBASIS-VIPP. Our training protocol will follow a 'cascade model', which has worked extremely well in existing collaborations between Prof Green and collaborators in India. Ms Taylor (therapy supervisor for the pilot RCT)[15] will provide initial training to the Research Therapists and a 'Research Champion. The Research Champion will provide day-to-day guidance to the Research Therapists, including fortnightly supervision sessions. Monthly video/teleconferences will be held between the Research Therapists, the Research Champions and Ms Taylor to monitor progress. CI Taylor will independently rate 5% of trial therapy tapes to test within-trial fidelity.

4.9 Expected duration of trial and participant participation.

The study will involve a recruitment telephone call (conducted by CDS staff), an eligibility screening telephone call (Research Therapist), a baseline assessment (Research Assistant), a treatment phase (Research Therapist) and three follow-up assessments (Research Assistant). At any time throughout the duration of the trial, participants will be able to contact the Trial Coordinator (Kandice Varcin) to discuss any aspect of the trial. The project timetable and milestones are presented in Table 1.

Supplementary Table 1. Project timetable and milestones

Milestone	Month
20. Study commences with ethics approval already obtained	1
21. MACI training in Manchester, UK.	1
22. iBASIS-VIPP Training meeting in Manchester, UK	2
23. Therapist training requirements met	3
24. Participant recruitment commences	4
25. Therapy commences for recruited participants	4
26. 1st follow-up assessments (6-months post-baseline) commence	9
27. 66 patients recruited and randomised	14
28. 2nd follow-up assessments (12-months post baseline) commence	15
29. All therapy sessions completed	25
30. All post-treatment assessment sessions completed	26
31. 3rd follow-up assessments (24-months post-baseline) commence	27
32. Participant recruitment concludes	27
33. 1st follow-up assessments (6-months post-baseline) conclude	30
34. Data analysis and write-up for 1st follow-up session (6-months post-baseline)	30
35. 2nd follow-up assessments (12-months post-baseline) conclude	36
36. Data analysis and write-up for 2nd follow-up session (12-months post-treatment)	36

37. 3rd follow-up assessments (24-months post baseline) conclude	48
38. Data analysis and write-up for 3rd follow-up session (24-months post-baseline)	48

Participant recruitment and eligibility screening

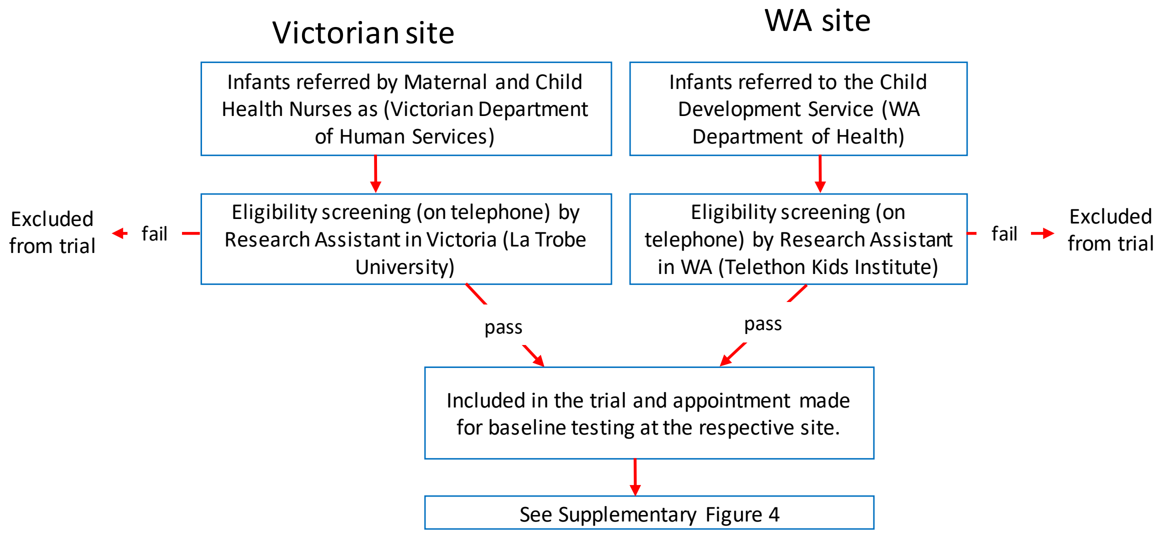

Supplementary Figure 3. Recruitment procedure at the Melbourne and Perth sites

At the WA site, staff at each of the 11 CDS 'intake' sites around the Perth metropolitan area will identify newly referred infants who may be eligible for the current study. The caregiver of any infant meeting the study criteria will be telephoned by a CDS staff member who will inform the families about the trial. The contact details of any consenting families will be sent immediately to the Research Assistant at the Telethon Kids Institute. The Research Assistant will then telephone the caregiver to conduct the eligibility screening, including verbal administration of the SACS-R.[34] For those who express an interest in participating, an appointment will be made to attend the Telethon Kids Institute for a Baseline Assessment.

At the Melbourne site, potential infant participants will be identified through Maternal and Child Health (MCH) nurse, who will perform SACS-R developmental screens on infants at 12-months of age.[34] Any infant who fails the standard screen for developmental delay used by the nurses will be informed about this study. Caregivers interested in having their infant participate in the trial will have their contact details sent immediately to the Research Assistant at La Trobe University. The Research Assistant will then telephone the caregiver by telephone to conduct the eligibility screening including repeat verbal administration of the SACS-R.[34] For those who express an interest in participating, an appointment will be made to attend La Trobe University for a Baseline Assessment.

Once an appointment has been made, the caregivers will be mailed an appointment confirmation letter, along with a Participant Information and Consent Form (PICF). The PICF contains a description of the project, information about random assignment to the trial conditions, procedures and time commitment, confidentiality and privacy, and contact information for the

researchers and the PMH and La Trobe University Ethics Committees. It also contains a statement that participants are free to withdraw from the study at any time and that participation is voluntary. These forms have been created in accordance with Good Clinical Practice guidelines. The parent(s)/caregiver(s) will asked to read the PICF, and encouraged to ask the research team any questions they may have at the Baseline appointment. The PICF includes the contact details of the study team, and Caregivers will be able to contact the study team prior to the Baseline appointment should they want to.

Baseline Assessment

Baseline assessments for will occur within four weeks of eligibility screening, and will be conducted by a Research Assistant based at each site. At the beginning of this session, the Research Assistant will ask the caregiver whether (a) they have read the PICF previously mailed to them, and (b) whether they had any questions. Research Assistants will also provide a document outlining the study stages in more detail, and talk caregivers through this document.

After all questions have been answered to the satisfaction of the caregiver, s/he will be asked to sign the consent forms and will be provided with an information sheet to keep. If not, they will be referred on to appropriate services. Upon consent, participants will be given the baseline assessment, which includes a suite of behavioural tests. See Supplementary Figure 4 for the trial procedure.

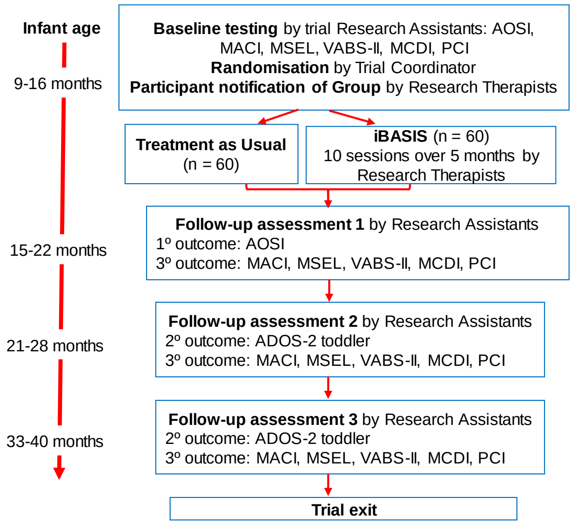

Supplementary Figure 4. Trial procedure

Randomisation

Following the baseline session, the Research Assistant will communicate to the Trial Coordinator (based in Perth) that the session has been completed and consent has been obtained. The Trial Coordinator will then conduct the randomisation and communicate the family's group to the Research Therapist at the relevant site.

Participant notification of group

The Research Therapist at each site will contact the participant to arrange for either the first iBASIS-VIPP therapy session ('iBASIS-VIPP' group), or current best practice ('Treatment as Usual' group).

'Treatment phase'

For participants allocated to the treatment group, iBASIS-VIPP therapy sessions will be conducted by a Research Therapist at each site and commence within two weeks of Baseline assessment. Families will receive 10 sessions at fortnightly intervals over five months, and will undertake 30-mins daily home practice (described in 4.6). Caregivers in the 'treatment as usual' group may be invited to attend a parent information workshop that provides information about

early interactions with infants, and/or be provided with reading materials on infant development, or offered any other services within the community that may be considered appropriate for the infant.

Follow-up assessment 1 (6-months post-baseline assessment)
Follow-up assessment 1 will be conducted by the trial Research Assistant at each site and will take place within two weeks of the final day of the 5-month treatment phase. Our preference is for these assessments to take place in a quiet clinic room at either the Telethon Kids Institute or La Trobe University. However, if this is not possible, the assessment will take place at the participants' homes. The assessment will comprise a suite of behavioural assessments as outlined in Supplementary Tables 2 and 3.

Follow-up assessment 2 (12-months post-baseline assessment)
Follow-up assessment 2 will be conducted by the trial Research Assistant at each site and will take place within two weeks of the 12-month anniversary of the infant completing the baseline assessment. Our preference is for these assessments to take place in a quiet clinic room at either the Telethon Kids Institute or La Trobe University. However, if this is not possible, the assessment will take place at the participants' homes. The assessment will comprise a suite of behavioural assessments as outlined in Supplementary Tables 2 and 3.

Follow-up assessment 3 (24-months post-baseline assessment)
Follow-up assessment 3 will be conducted by the trial Research Assistant at each site and will take place within two weeks of the 24 month anniversary of the infant completing the baseline assessment. Our preference is for these assessments to take place in a quiet clinic room at either the Telethon Kids Institute or La Trobe University. However, if this is not possible, the assessment will take place at the participants' homes. The assessment will comprise a suite of behavioural assessments as outlined in Supplementary Tables 2 and 3.

Supplementary Table 2. Schedule of assessments.

	Telephone screening	Baseline	Follow-up assessment 1	Follow-up assessment 2	Follow-up assessment 3
**Basic information**					
Study month commencing	1	2	8	14	26
Infants' age (months)	9-16	9-16	15-22	21-28	33-40
**Eligibility**					
SACS-R	X				
Inclusion/exclusion criteria	X				
Infant medical history	X				
Informed Consent		X			
**Questionnaires**					
Family questionnaire		X	X	X	X
MCDI		X	X	X	X
VABS-II		X	X	X	X
PSOC		X	X	X	X

**Face-to-face assessment**					
AOSI		X	X		
ADOS-2			X	X	X
PCI		X	X	X	X
MSEL		X	X	X	X
Clinical Best Estimate Diagnosis					X

Supplementary Table 3. Psychometric tests delivered at the baseline and follow-up assessments by a Research Assistant who is blind to the infant's group status.

**Name**	**Details**
Autism Observation Scale for Infants (AOSI)[36]	**Description:** Semi-structured assessment developed specifically to elicit early behavioural risk factors of ASD in infants aged 6-18 months.<br>**Procedure**: Administered by researchers trained to protocol fidelity, and scored from videotape by researchers with demonstrated coding reliability (>80% agreement) who are blind to group allocation.<br>Scoring: Individual item scores (range 0-2) for 18 behaviours summed to produce AOSI Total Score (range 0-50; higher scores = more ASD symptoms).<br>**Validity:** Higher mean number of behavioural 'markers' at 12 and 18 months in infants with later ASD vs. no ASD[12,37]; acceptable test-retest (≥ .61) and inter-rater reliability (≥ .92)[36] for AOSI Total Score.
Autism Diagnostic Observation Schedule – Second edition (ADOS-2): Toddler Module[37]	**Description:** Semi-structured assessment developed to elicit behaviours relevant to an ASD diagnosis in toddlers 12-30 months (Toddler Module) or aged 31 months and above (Module 1 and 2).<br>**Procedure:** Administered by researchers trained to protocol fidelity, and scored from videotape by researchers with demonstrated coding reliability (>80% agreement) who are blind to group allocation.<br>**Scores:** Continuous symptom measure: Total Algorithm Score (range 0-28; higher = more symptoms). Binary diagnostic classification: meets/does not meet established cut-off for ASD (i.e., non-verbal toddlers ≥ 12; verbal ≥ 10).<br>**Validity:** Continuous measure: Excellent test-retest and inter-rater agreement (Intra-class correlations ≥ .90). Binary classification: Excellent sensitivity & specificity of cut-off scores for identifying ASD vs. other disabilities (≥. 81).[38]
Parent Child Interaction (PCI)	**Description:** Parent-infant free-play interaction sample. Filmed for 10mins, with standard set of age-appropriate toys provided.<br>**Procedure:** Footage coded by blinded trained researchers using Manchester-Assessment of Caregiver-Infant Interaction (MACI) procedures. Subset (10%) of footage scored by Dr Ming-Wae Wan for inter-rater reliability.<br>**Scoring:** Footage coded on 7-point Likert-type scales (higher scores = better quality interaction) for parent behaviour (sensitive responsiveness, non-directiveness), infant behaviour (affect, liveliness, attentiveness to parent), and dyadic interaction (engagement intensity, mutuality).<br>**Validity:** Low scores on MACI infant scales predict ASD diagnosis at 3 years.[12,13]
Mullen Scales	**Description:** Standardised developmental assessment norm-referenced 0 - 68

of Early Learning (MSEL)[39]	months.<br>**Procedure:** Trained, blinded assessors administer tasks directly with infants.<br>**Scoring:** Standardised t-scores (M = 50; SD = 10) and age equivalents computed for each sub-scale (higher scores = better developed skills).<br>**Validity:** Excellent test-retest and inter-scorer reliability for ages ≤ 24 months (r ≥ .82).[36] Infants at high risk of ASD score consistently poorly.[40-41]
Vineland Adaptive Behavior Scales – 2nd edition (VABS-II)[42]	**Description:** Caregiver-report measure of functional skills, norm-referenced 0 - 90 years. Communication and Socialization domains relevant to current trial.<br>**Procedure:** Parent completes form (i.e., not blind to group allocation) which is scored up by researcher according to manualised procedures.<br>**Scoring:** Domain Standard Scores (M = 100; SD = 15), and subdomain v-scores (M = 15; SD = 3) and age-equivalents computed (higher scores = better skills).<br>**Validity:** Excellent test-retest and inter-rater reliability for ages ≤ 24 months (r ≥ .81)[41] and evidence of low scores in 'familial high risk' infants.[18,30]
MacArthur Communicative Development Inventory (MCDI)[43-44]	**Description and Procedure:** Caregiver-report checklists tallying Receptive and Expressive Vocabulary knowledge in infants aged 8-30 months. Words and Gestures (WG) form used at Baseline and F1 and Words and Sentences (WS) form used at Follow-up 2 and 3.<br>**Scoring:** Endorsed items summed to yield raw Receptive and Expressive vocabulary counts (possible range 0-680; higher score = more language skill).<br>**Validity:** Very strong internal consistency (r ≥ .95)[44]
Parenting Sense of Competence (PSOC)[45] and Depression Anxiety and Stress Scales (DASS)[46]	**Description and Procedure:** Caregiver-report questionnaires concerning sense of efficacy and stress in the parenting role: Completed at Baseline and the three Follow-up Assessments.<br>**Scoring:** Endorsed PSOC items summed to yield Satisfaction, Efficacy and Interest in being a Parent subscales, and for DASS items aggregate across domains of depression, anxiety and stress in the parent.<br>**Validity:** Shown to differentiate parents of infants at high-risk of ASD from low-risk controls
Clinical Best Estimate Diagnosis	**Description:** Clinicians experienced in ASD diagnosis will review data collected and provide an appraisal of ASD diagnostic outcome, similar to previous trials.[35]

4.10 Criteria for termination of the trial

Discontinuation criteria for the trial reflect guidelines set out by the TGA. The trial will be terminated in the case that support for the trial is withdrawn from the HREC or sponsor. This includes the following possibilities:

- Evidence of significant deviation from the trial protocol that may threaten wellbeing of participants;
- Evidence that allowing the trial to continue carries an unacceptable risk of death, serious injury or illness to participants;
- Evidence that one treatment proves to be so much better or worse that to continue would disadvantage one group of participants;
- Evidence that conduct of the trial is in breach of the law.

In regards to risk to participants, in the unlikely event of an SAE, a review would be conducted involving the SAC and the HREC board in relation to the future continuation of the trial. If the SAE is definitely due to trial participation, it may result in discontinuation of the whole trial (recruitment and treatment), depending on the decision made by the SAC and HREC based on available evidence concerning risk to participants. If the SAE is found to be possibly or probably due to trial participation, it will result in the affected participant/s immediately ceasing involvement in the trial. The SAC will then evaluate how the overall trial should proceed.

4.12 Identification of source data

In this trial, the following documents will be considered source data:

- Hard copies of the following caregiver-report questionnaires:
  - Demographics Information
  - MCDI
  - VABS-II
  - PSOC
- Hard copies of the following protocols completed by Research Assistants or independent clinicians:
  - AOSI
  - ADOS-2
  - MSEL
  - Clinical best estimate diagnosis
- Hard copies of the following Research Therapist reports:
  - Fidelity rating scale
- Digital video footage of the following:
  - AOSI assessment
  - ADOS-2 assessment
  - PCI samples
  - In-home iBASIS-VIPP therapy sessions

8. SOURCE AND SELECTION OF PARTICIPANTS

8.1 Source and feasibility of participant recruitment

Infants will be recruited from two sources. At the WA site, staff at each of the 11 CDS 'intake' sites around the Perth metropolitan area will identify newly referred infants who may be eligible for the current study. The caregiver of any infant meeting the study criteria will be telephoned by a CDS staff member who will inform the families about the trial. The contact details of any consenting families will be sent immediately to the Research Assistant at the Telethon Kids Institute. The Research Assistant will then telephone the caregiver to conduct the eligibility screening, including verbal administration of the SACS-R[34] For those who express an interest in participating, an appointment will be made to attend the Telethon Kids Institute for a Baseline Assessment.

At the Melbourne site, potential infant participants will be identified through MCH nurses, who will perform developmental screens on infants at 12-months of age. Any infant who fails the

standard screen for developmental delay used by the nurses will be informed about this study. Caregivers interested in having their infant participate in the trial will have their contact details sent immediately to the Research Assistant at La Trobe University. The Research Assistant will then telephone the caregiver by telephone to conduct the eligibility screening, including verbal administration of the SACS-R. For those who express an interest in participating, an appointment will be made to attend La Trobe University for a Baseline Assessment.

5.2 Participant inclusion criteria

Infants will be offered participation in this trial if they:

(d) Are between 9 and 16 months of age;
(e) Endorsement of three or more of the 'key items' on the 12-month old SACS-R[34] (absent/atypical pointing, waving, imitation, eye contact, response to name).
(f) The primary caregiver involved in the trial speaks sufficient English to: (i) understand the requirements of the study, and (ii) is deemed to be able to participate fully in the therapy sessions.

5.4 Participant exclusion criteria

Participants will be excluded from the study if they meet either of the following characteristics:

(a) The infant has a diagnosed comorbidity (or significant clinical indicators of a comorbidity) known to affect neurological and developmental abilities (e.g., preterm birth < 32 weeks, cerebral palsy, Down syndrome, Rett syndrome, other chromosomal abnormality, hearing/visual impairment), and/or
(b) The family does not intend to remain living in the Perth/Melbourne areas for the next two years.

5.4 Participant withdrawal criteria

A premature discontinuation will occur when a participant for whom informed consent was obtained ceases participation in the study, regardless of circumstances, before the completion of the study. If a participant must be prematurely discontinued from the study, the primary reason for discontinuation must be recorded as one of the following:

- Failure to meet inclusion/exclusion criteria;
- The participant's home is deemed by the Trial Coordinator to not provide a safe and healthy working environment for the Research Therapist.
- SAE linked to iBASIS-VIPP;
    - In the unlikely event that the participant's psychological state deteriorates as a result of treatment they would be withdrawn from the study and alternative therapy or referral offered.
- Protocol deviation/violation, including lack of compliance;
- An infant participant received a diagnosis with a condition that meets 'exclusion (a)'. In these cases, we will ensure that the participant has received an appropriate health and medical referral for their diagnosis (e.g., GP, paediatrician, audiologist etc.) at the time of their withdrawal from the study.
- Voluntary withdrawal of consent (reasons will be documented);
- Lost to follow-up (every effort will be made to contact the patient first; a certified letter will be sent).

Data collection for withdrawn subjects:
All enrolled participants who prematurely discontinue from the study regardless of cause will be asked to come in for a final evaluation. A final evaluation is defined as completion of the evaluations scheduled for the final post-treatment assessment (Follow-up assessment 3). Parents/caregivers refusing to have their infant assessed must be requested in writing to come in for a visit. A copy of the letter will be kept by the PI together with the source documentation. The reasons for premature discontinuation from the study will be reflected on the Study Termination Record of the CRF.

Patient replacement procedures
Patients in this study who prematurely discontinue from the study will not be replaced.

9. TREATMENT OF PARTICIPANTS

9.1 Description and justification for the treatments

Information concerning the theory and development of the behavioural intervention is detailed in the 'Rationale and Background' section, and is summarised here.

Name:
(1) iBASIS-VIPP
(2) Treatment as usual

Treatment schedule:
Participants in the 'iBASIS-VIPP' group will receive 10 therapy sessions over a period of 5 months. Parents will be asked to undertake 30 minutes home practice with their infant daily. The 'Treatment as Usual' group will receive current treatment protocol offered within community health services, which varies considerably and may include a referral to a developmental paediatrician, a wait-list period (2-3 months), invitation to parent information sessions, and speech pathology assessment and intervention.

9.2 . The medications/interventions permitted during the trial.

At the outset of the study, parents/carers will be asked to report all medications the child is taking, including the reason for taking the medication and duration for which medication has been taken to date. Participation in this trial does not prevent participants' seeking behavioural therapy outside of the trial framework. The nature and quality of any outside therapy and medications received will be recorded at the Follow-up Assessments.

6.3 Procedure for monitoring participant compliance

Compliance in the 'iBASIS-VIPP group' will be monitored via a compliance checklist completed by the Research Therapist at each face-to-face session, and by the parent/caregiver following each daily practice session.

10. ASSESSMENT OF EFFICACY

7.1 Specification of efficacy parameters and methods and timing for assessing and recording

Unless otherwise specified, all efficacy parameters will be recorded in source format. This will then be entered into an electronic database where it will be stored alongside participant's ID numbers. The database will be regularly checked for accuracy, and source documents will be retained to clarify any issues.

Autism Observation Scale for Infants will be used to measure the severity of autistic symptoms.
Method of Assessment: Researcher-administered semi-structured play assessment with infant. Scored from video by researcher with demonstrated high level reliability, who is blind to treatment group.
Timing of assessment. Baseline, Follow-up Assessment 1.

The Toddler Module of the Autism Diagnostic Observation Schedule – Second Edition will be used to measure the severity of autistic symptoms.
Method of Assessment: Researcher-administered semi-structured play assessment with toddler. Scored from video by researcher with demonstrated high level reliability, who is blind to treatment group.
Timing of assessment. Follow-up Assessment 2 and 3.

The Manchester Assessment of Caregiver-Infant Interaction will be used to measure the quality pf parent-infant interactions.
Method of Assessment: Parent-Infant free play interaction sample.
Scored from video by researcher with demonstrated high level reliability, who is blind to treatment group.
Timing of assessment. Baseline, Follow-up assessments 1, 2 and 3.

The Mullen Scales of Early Learning will be used to assessed cognitive and motor ability
Method of Assessment: Researcher-administered standardised developmental assessment.
Scored live by researcher administering the assessment.
Timing of assessment. Baseline, Follow-up assessments 1, 2 and 3.

The Vineland Adaptive Behavior Scales – 2nd edition will be used to assess daily functional skills.
Method of Assessment: Caregiver-report questionnaire
Scored by researcher.
Timing of assessment. Baseline, Follow-up assessments 1, 2 and 3.

The MacArthur-Bates Communicative Development Inventory will be used to assess early language development.
Method of Assessment: Caregiver-report questionnaire
Scored by researcher.
Timing of assessment. Baseline, Follow-up assessments 1 (WG form), and 2 and 3 (WS form).

The Parent Sense of Competence will be used to assess parenting self efficacy.
Method of Assessment: Caregiver-report questionnaire
Scored by researcher.
Timing of assessment. Baseline, Follow-up assessments 1, 2 and 3.

Clinical ASD diagnostic outcome will be assessed by experienced independent clinicians.
Method of Assessment: Independent clinician review of collected data.
Scored by independent clinicians
Timing of assessment. Follow-up assessment 3.

7.2 Methods for analysing efficacy parameters

This is detailed in section 'Statistical analysis plan'.

8 ASSESSMENT OF SAFETY

8.6 Summary of known and potential risks and benefits, if any, to research participants.

iBASIS-VIPP is a behavioural intervention that supports parents to interact with their infant to maximise social and communication development. The potential benefit of this behavioural intervention is a reduction in autistic symptomatology, and an increase in developmental abilities, such as language and social skills. There are no known risks of this intervention, and our previous studies have shown that this therapy is well tolerated by families. The safety issues for this trial are negligible.

8.7 The safety parameters and method for assessing and recording safety parameters.

Safety parameters
Key safety parameters in the trial are occurrence of side effects, type of side effects, occurrence of adverse events, type of adverse event, occurrence and type of intercurrent illnesses across the trial.

Method for assessing and recording
Parents/infants in the iBASIS-VIPP group will have face-to-face therapy sessions 10 times throughout the 5-month treatment period. Parents will also be asked to complete 30 minutes practice each day. At the end of each therapy session, the Research Therapist will record if there have been any adverse effects of the therapy noted during the session, as well as during the period since the last testing session. This will be recorded on the Case Report Form completed by the Research Therapist.

8.8 Details of the Data and Safety Monitoring Board, or equivalent.

An independent Data and Safety Monitoring Committee (DSMC) has been assembled to monitor the progress, safety and efficacy of this clinical trial and provide critical evaluation and recommendations to the study investigators and all sponsors of this trial. The members of the DSMC serve in an individual 'expert' capacity. The DSMC will meet every 6 months throughout

this trial and review cumulative study data to evaluate study conduct, the scientific validity and data integrity of the study including safety of this trial. Scheduled meetings will be supplemented with additional meetings or conference calls scheduled as needed.

8.9 The procedures for eliciting reports of and for recording and reporting adverse events. Include definitions of adverse events.

We define an adverse event as any untoward medical occurrence in a patient or clinical investigation subject associated with the experimental treatment. The treatment protocol is focused solely on teaching parents how to modify their interactions with their infants, and therefore we believe the risk of any adverse events is extremely low. Caregivers will be provided with monitoring forms at the outset of the study to record any side effects they believe may be associated with the treatment. These forms will provide space to record side effects daily if needed.

Any adverse event will be reported to the PMH and LTU Human Research Ethics Committees in a detailed, formal written report, which will identify participants involved by study code rather than name or address. These reports will include an assessment of the severity and causality of the adverse event in relation to the treatment protocol (see Table 4), as well as detailed information regarding the steps taken by the Study Team with regard to both the treatment protocol and the participant involved.

Supplementary Table 4. The assignment of causality for each AE should be made by the investigator responsible for the care of the participant based on the definitions in the table below.

Relationship	Description
**Unrelated**	Sufficient information exists to indicate that the aetiology is unrelated to the treatment protocol.
**Unlikely**	When there is no reasonable temporal association between the treatment protocol and the suspected adverse event. The event could have been related to the patient's clinical state or concomitant treatment(s).
**Possible**	There is some evidence to suggest a causal relationship (e.g. because the event occurs within a reasonable time after administration of the treatment session). However, the influence of other factors may have contributed to the event (e.g. the participant's clinical condition, other concomitant treatments).
**Definitely**	There is clear evidence to suggest a causal relationship, based on: temporal relationship to the treatment session
**Not assessable**	When causality is, for one reason or another not accessible, e.g. because of insufficient evidence, conflicting data or poor documentation

8.10 The type and duration of the follow-up of participants after adverse events.

Adverse events will be monitored by the Research Therapists at each iBASIS session, which take place at fortnightly intervals during the 5-month treatment period. If an adverse event is reported by the caregiver, infant wellbeing will be followed up by study medical staff through phone calls and visits as needed (depending on the nature of the adverse event). Participants will be made

aware that they are able to contact study staff regarding the adverse event during this period, as well as receiving contact from study medical staff. Any adverse event reported at the Perth site will be immediately communicated through Professor Whitehouse to the CDS Director (Ms Emma Davidson) and the CDS Senior Clinical Advisor (Clinical Associate Professor John Wray).

Any parent who yields a score above the 'clinical cutoff' on any of the DASS scales[46] (Depression ≥ 14, Anxiety ≥ 10, Stress ≥ 17) will be contacted by a study researcher with clinical training (Speech Pathologist, Clinical/Developmental Psychologist or medical doctor) and informed about their high score. The study researcher will encourage the parent to make an appointment with their GP to discuss the implications of this score.

10. DATA MANAGEMENT, STATISTICAL ANALYSIS AND RECORD KEEPING

9.1 Description of the statistical methods to be employed, including timing of any planned interim analysis.

There will be two analysis phases, which are described in a separate 'Statistical analysis plan'.

10.2 The number of participants planned to be enrolled

We will recruit 132 infants into this trial (n = 66 in each group), which has been chosen for reason of statistical power, detailed in 'AICES analysis plan'.

10.3 The level of significance to be used.

The alpha level will be set at $p < .05$

10.4 Procedures for reporting any deviation(s) from the original statistical plan,

Any deviations from the original statistical plan will be described and justified in the protocol.

10.5 The selection of participants to be included in the analyses

Data will be analyzed through an 'intention to treat' approach and analyses will include all randomized participants.

10.6 Information on how data will be managed, including coding for computer analysis and data handling (collection, storage, maintenance, security and archiving). Include details regarding these processes if the data is sent off-site (e.g. encryption).

Upon entry into the study, participants will be allocated a unique ID, and all data will be referenced to this code. Data collection will be conducted only by authorised members of the study team who are named on the HREC application for the trial.

For the duration of the trial, source data from Perth and Melbourne will be stored at the respective sites. At the Perth site, baseline and follow-up assessments will be kept in a locked filing cabinet in Prof Whitehouse's office at Telethon Kids Institute. Source data for the iBASIS-VIPP therapy protocol will be stored in a locked filing cabinet at the West Perth Child Development Service, which is where the Research Therapist will be based. At the Melbourne site, baseline and follow-up assessments will be kept in a locked filing cabinet at the Olga

Tennison Autism Research Centre (OTARC), LTU. Source data for the iBASIS therapy protocol will be stored in a locked filing cabinet in Dr Hudry's office at LTU.

As the trial progresses, Research Assistants and Therapists will enter source data through a web portal into an electronic database on an online, secure, password-protected cloud computing database. All study data for analysis purposes will be entered into REDCap (which stands for Research Electronic Data Cpature), while at the Melbourne site, participant information will be stored in Salesforce.com Customer Relationship Management (CRM) system for scheduling purposes. Both REDCap and Salesforce databases are only accessible by authorised personnel, including researchers and administrative support staff. Salesforce.com CRM security and privacy credentials and policies can be found at https://trust.salesforce.com/trust/ and http://www.salesforce.com/au/company/privacy/, respectively. REDCap is a server-based software so can only be installed on a local web server by Institutional IT staff who then allows access to study personnel through a web browser. Only sufficiently trained and supervised research assistants will be hired to enter data. An audit of data files over time to ensure completeness of data collection will be conducted on a routine basis. Video footage of recorded as part of the study protocols will be labelled with the ID the infants/caregivers and be kept on a secure server at the Telethon Kids Institute. At the conclusion of the trial, all source data will be transferred to the Telethon Kids Institute for storage in a locked-filing cabinet in Prof Whitehouse's office. Source data will be kept for a minimum of 15 years from the completion of the trial. At the Perth site, hard copies of data recorded at the baseline assessment, therapy sessions (if received), and first follow-up assessment will also be provided to the CDS treating clinician at the conclusion of the latter assessment.

The biological samples will be labelled with a de-identified ID. However, as this study constitutes clinical and genetic information, it is critical to have the ability to re-identify participants. Three people only will have access to the "key" document that contains both participant IDs and contact details (including name, date of birth, phone numbers, email and postal address). Andrew Whitehouse and Kristelle Hudry will hold an electronic copy of the key document on a password protected document server at their respective institutions (Telethon Kids Institute and La Trobe University). The Tissue Bank manager at the Wesley Research Institute, Emma Raymond, will also hold a copy of consent forms and have access to a 'key' document matching names and ID codes. This will be stored on a secure server at the Wesley Research Institute in order to comply with Tissue Bank policies for long-term data retention. Approval to access these 'key' documents will be governed by the Data Access Committee.

10.7 Procedure for accounting for missing, unused, and spurious (*false*) data.

Analyses of data involving missing measures will be undertaken wherever possible either by maximum-likelihood estimation or by the use of multiple imputation, carried out using the iterative chained equation approach as implemented in Stata. Both approaches allow for potentially selective but non-informative attrition.

11 MONITORING

14.1 Statement that the trial investigators/institutions will permit trial-related monitoring, audits, and regulatory inspections, providing direct access to source data/documents.

The investigators and institution will permit trial-related monitoring, audits, and regulatory inspections, providing direct access to source documents, as required. Trial monitoring may be undertaken by the HREC, sponsor, and TGA. This will be stipulated in the Participant Information Statement.

15 QUALITY CONTROL AND QUALITY ASSURANCE

11.3 Statement that the trial will be conducted in compliance with the protocol, Good Clinical Practice and the application regulatory requirements.

The trial will be conducted in compliance with the protocol, Good Clinical Practice and the relevant regulatory requirements.

11.4 Quality control & quality assurance measures to ensure quality of data.

The first three months of the project will be spent providing the Research Therapist with comprehensive training to fidelity in iBASIS-VIPP. Our training protocol will follow a ‘cascade model’, which has worked extremely well in existing collaborations between PI Green and collaborators in India. Ms Taylor (therapy supervisor for the pilot RCT)[15] will provide initial training to the Research Therapists and up to two ‘Research Champions’. The Research Champions will be based in Perth and provide day-to-day guidance to the Research Therapists, including fortnightly supervision sessions. Monthly video/teleconferences will be held between the Research Therapists, the Research Champions and Ms Taylor to monitor progress. Ms Taylor will independently rate 5% of trial therapy tapes to test within-trial fidelity.

Any changes to the Clinical Trial Protocol will be documented and separate versions kept on file. Our trial will also be recorded in an online clinical trial database, as is standard practice for the conduct of clinical trials.

16 ETHICS

16.1 Description of ethical considerations with particular reference to participant consent

This application will be reviewed and fully approved in writing by the PMH Ethics Committee. A number of potential ethical issues that may arise in the course of the trial have been identified and discussed at length in the HREC application for this trial.

Informed consent:
The study will recruit infants aged between 9 and 16 months, who are showing behavioural ‘warning signs’ of ASD. These infants are unable to provide informed consent. We address this issue by their parent/guardian/carer provide informed consent on their behalf.

Uneven relationships.

At the Perth site, some infants who are offered participation in this study may be patients of a study researcher, A/Prof John Wray. On these occasions, Dr Wray will emphasise to caregivers that they are not obliged to take part in the study and that they will not compromise their relationship with him as a clinician if they choose not to participate. All participant information statements will clearly emphasise that participation in this study is voluntary, there will be no reimbursement for participation, and participants are free to withdraw at any time, as well as highlighting possible risks and benefits of participation.

Behavioural assessments:
Infants will undergo several behavioural assessments, each lasting no more than 2.5 hours, which will take place at either the Telethon Kids Institute (Perth site) or La Trobe University (Melbourne site). This poses no risk to the children. The clinical examination will be undertaken by experienced research assistants who will make a judgment on whether any child experiences undue levels of distress. If so, the clinical examination will cease

Storage of personal information:
Parents will complete a questionnaire asking about the medical and social history of the child and family. Personal information relating to research participants, themselves, and/ or their parents/ or child/ren will be stored on an online, secure, password-protected cloud computing database, known as Salesforce.com Customer Relationship Management (CRM) system and is only accessible by authorised personnel, including researchers and administrative support staff. Salesforce.com CRM security and privacy credentials and policies can be found at https://trust.salesforce.com/trust/ and http://www.salesforce.com/au/company/privacy/, respectively.

Video footage will also be recorded and stored as part of the intervention and assessment protocols. Some of this information could be considered personal. Parents will provide written consent for the collection of this information. Upon recruitment participants will be allocated a code number. Source data (including digital files of video footage), as well as electronic copies of source data will be labelled with their code number only. The sole electronic file linking personal details with these code numbers will be stored on a password protected server at the TKI and LTU. There will be no hard copy of this document.

Limited disclosure of ASD-related hypotheses
While the aim of this RCT is to determine whether a novel behavioural therapy can mitigate the onset of ASD behaviours in later childhood, the Participant Information and Consent Form does not prominently feature the term 'autism' or Autism Spectrum Disorder'.

This decision was based on the following reasoning:
1. The infants recruited into this trial will have been flagged as having early social and communication difficulties (not ASD). Our previous studies have found that these infants are at increased risk of ASD. However: (a) the majority of these infants (~60%) will not develop ASD, and (b) there is currently no reliable way to predict which infants will and won't develop ASD.
2. Our considerable clinical and research experience has found that even mentioning 'autism' or 'ASD', even in terms of 'increased risk for ASD' can cause family significant distress.

3. Weighing up points #1 and #2, we feel that it is most ethical to discuss their infants' developmental problems in terms of 'language and communication difficulties' and not 'increased ASD risk'.

It is possible that families may link their infant's difficulties with ASD through the course of this study. For example, many of the Investigators have conducted previous research into ASD, and families may google this information. To allay concerns, we have included a specific item in the Participant Information and Consent Form:
"Please do not be concerned that your child may have more serious developmental concerns, such as autism. It is not possible to know whether an infant younger than 18 months of age has autism. The vast majority of children who have early social and communication difficulties do not grow up to have autism."

17 BUDGET, FINANCING, INDEMNITY AND INSURANCE.

13.2 Budget, financing, indemnity and insurance, if not addressed in a separate agreement.

This project is funded by grants from the Telethon-Perth Children's Hospital grant, the Autism Cooperative Research Centre, the Understanding Disease RFA at LTU, and the Angela Wright Bennett Foundation.

This clinical trial will be undertaken with adequate insurance/indemnity to cover the liability of the investigators and sponsor. In the unlikely case of injury suffered by participants as a result of their participation in the study, participants will be referred to their General Practitioner, who will assist them in arranging appropriate medical treatment. Participants will also be informed that they may have a right to take legal action to obtain compensation for any injuries or complications resulting from the study. Compensation may be available if the participants' injury or complication is sufficiently serious and is caused by unsafe drugs or equipment, or by the negligence of one of the parties involved in the study. If they receive compensation that includes an amount for medical expenses, they will be required to pay for their child's medical treatment from those compensation monies. If the participant is not eligible for compensation for their injury or complication under the law, but are eligible for Medicare, then they can receive any medical treatment required for their injury or complication free of charge as a public patient in any Australian public hospital. These guidelines will be outlined on the participant information statement.

18 PUBLICATION

14.1 Publication and dissemination of trial results (including any limitations).

In accordance with the guidelines stipulated by the funding bodies, the results of this study will be disseminated as widely as possible into the scientific and broader community. This will include publication in peer-reviewed journals, scholarly book chapters, presentation at

conferences, publication in conference proceedings and student theses. Publications arising from this project will be deposited into an open access institutional repository, where possible. Results will also be disseminated into the wider community in a format appropriate for laypeople, through links including our website and Facebook pages, as well as newsletters and in presentations.

Summary of Changes

Amendment	Description of Amendment	Date of change
Revision to more accurately describe community treatment as usual.	The ‘treatment as usual’ section in Section 4.7 to more accurately describe community ‘treatment as usual’.	16th December 2016
Revision to more accurately describe community treatment as usual.	The following sentence (in Trial Summary) was changed from:<br>“Families in the ‘Treatment as Usual’ group will receive a parent information seminar, which is the current ‘best practice’ protocol for these infants.”<br><br>to<br><br>“Families in the ‘Treatment as Usual’ group will receive current treatment protocol offered within the community which may comprise a parent information seminar, the provision of reading materials on infant development, or developmental monitoring.”<br><br>This amendment was made after the first participant was enrolled but prior to recruitment of the final participant.	16th December 2016
Correction of typo error in randomization description.	A typo in the following sentence (Section 4.8) was identified, and amended from:<br>“Group allocation of participants will be by minimization method, stratified by child gender, site (Perth, Melbourne), score on the Social Attention and Communication Scale–Revised (SACS-R) 12-month checklist (i.e., 3, 4, or 5)[34] and age band at **baseline appointment** (9-11 months/12-14 months).”<br><br>to<br><br>“Group allocation of participants will be by minimization method, stratified by child gender, site (Perth, Melbourne),	16th December 2016

	score on the Social Attention and Communication Scale–Revised (SACS-R) 12-month checklist (i.e., 3, 4, or 5)[34] and age band at **eligibility screen** (9-11 months/12-14 months)."	
Inclusion of Parenting Sense of Competence scale as a secondary variable	This secondary outcome was included to measure parenting satisfaction, which we can plausibly hypothesise may be improved by the experimental intervention. This change was made after enrolments closed, but prior to linkage of the treatment allocation variable to outcome data (i.e., completion of the final treatment-endpoint assessment), and prior to treatment unblinding.	6th August 2018
Amendment of statistical analysis plan.	This amendment describes a change in the analysis plan. The analysis plan introduced at this point was for two analysis phases. The first analysis phase assessed outcomes at immediate treatment endpoint only. These data were published in Whitehouse et al.[47]. The second analysis phase incorporated data from the three follow-up assessments (immediate treatment endpoint, 12-month post-baseline, and 24-month post-baseline). For both analysis phases, ASD symptoms were the primary outcome, and a range of other developmental outcomes were the secondary outcomes. This change is reflected throughout the trial protocol.<br><br>A description of the two analysis phases were taken out of the trial protocol, and instead described in a separate Statistical Analysis Plan. This Statistical Analysis Plan was registered on the ANZCTR on 6th August 2018. This change was made (and registered on ANZCTR; ANZCTR12616000819426) after enrolments closed, but prior to linkage of the treatment allocation variable to outcome data (i.e., completion of the final treatment-	6th August 2018

	endpoint assessment), and prior to treatment unblinding.	
