# Supplementary material for: Effect of Preemptive Intervention on Developmental Outcomes Among Infants Showing Early Signs of Autism: A Randomized Clinical Trial of Outcomes to Diagnosis
Source: JAMA Pediatr. 2021 Sep 20;175(11):e213298. doi: 10.1001/jamapediatrics.2021.3298 (PMC8453361; doi:10.1001/jamapediatrics.2021.3298)
Supplement: Supplement 2. — eMethods 1. iBASIS-VIPP Intervention Protocol eMethods 2. iBASIS-VIPP Intervention Fidelity Rating Scale eMethods 3. Additional Detail on Methods eMethods 4. Protocol for Clinical Best Estimate Judgment of Case Diagnosis eTable 1. Usual Community Care Received by Participants in the iBASIS-VIPP and Usual Care Groups That Was Not Associated With the Clinical Trial eTable 2. Consensus Diagnostic Groups by DSM-5 Diagnostic Criteria eTable 3. Outcome Data for Children Meeting DSM-5 Criteria for Autism Spectrum Disorder eTable 4. Primary and Secondary Outcome Data Across Assessment Points by Site eTable 5. Primary and Secondary Outcome Data Across Assessment Points by Sibling Status eFigure 1. Forest Plot of Comparison Between Treatment Groups by Autism Spectrum Disorder Diagnostic Criteria eFigure 2. Area Between Curves for Secondary Outcomes eReferences [file jamapediatr-e213298-s002.pdf]

Supplementary Online Content

Whitehouse AJO, Varcin KJ, Pillar S, et al. Effect of preemptive intervention on developmental outcomes among infants showing early signs of autism: a randomized clinical trial of outcomes to diagnosis. *JAMA Pediatr*. Published online September 20, 2021.
doi:10.1001/jamapediatrics.2021.3298

eMethods 1. iBASIS-VIPP Intervention Protocol

eMethods 2. iBASIS-VIPP Intervention Fidelity Rating Scale

eMethods 3. Additional Detail on Methods

eMethods 4. Protocol for Clinical Best Estimate Judgment of Case Diagnosis

eTable 1. Usual Community Care Received by Participants in the iBASIS-VIPP and Usual Care Groups That Was Not Associated With the Clinical Trial

eTable 2. Consensus Diagnostic Groups by *DSM-5* Diagnostic Criteria

eTable 3. Outcome Data for Children Meeting *DSM-5* Criteria for Autism Spectrum Disorder

eTable 4. Primary and Secondary Outcome Data Across Assessment Points by Site

eTable 5. Primary and Secondary Outcome Data Across Assessment Points by Sibling Status

eFigure 1. Forest Plot of Comparison Between Treatment Groups by Autism Spectrum Disorder Diagnostic Criteria

eFigure 2. Area Between Curves for Secondary Outcomes

eReferences

This supplementary material has been provided by the authors to give readers additional information about their work.

© 2021 Whitehouse AJO et al. *JAMA Pediatrics.*

eMethods 1. iBASIS-VIPP Intervention Protocol*

Samina Holsgrove[1], Ming Wai Wan[1], Janet McNally[1], Rhonda Booth[2], Carol Taylor[1],Vicky Slonims[2], Jonathan Green[1]

[1]Institute of Brain, Behaviour and Mental Health, University of Manchester, Manchester, UK

[2]Newcomen Centre, Guy's and St Thomas' NHS Foundation Trust and King's College, London, UK

Acknowledgement

Much of the procedural manual contained here is based on or adapted from the original work and manual of the Video Interaction to Promote Positive Parenting (VIPP)[27] www.leidenattachmentresearchprogram.eu/vipp/welcome/en/

*The full procedural manual is available separately from the authors

© 2021 Whitehouse AJO et al. *JAMA Pediatrics.*

AIM

Enrichment of the social interactive environment of the developing infant using a parent-mediated programme to enhance early social engagement and reciprocity.

The intervention strategy

The iBASIS intervention strategy is a parent-mediated approach to achieve two goals;

1) general enrichment of the core interactive social experience for infants 6-18 months inclusive
2) specific attention within this to addressing any emerging atypicalities that might be expected in prodromal autism at this age and their interactional consequences

The *i*-BASIS programme comprises up to twelve home based two hourly sessions over a period of 5 months. The programme is individualised to the needs of each dyad but core procedures are taken from the Video feedback Intervention to promote Positive Parenting (VIPP)[1], www.leidenattachmentresearchprogram.eu/vipp/welcome/en/). We chose this as the basis because its method (video-aided and parent-mediated using a direct work with parent and infant) is similar to that which we have used intensively with preschool children with diagnosed ASD[2-3] (www.manchester.ac.uk/medicine/pact) and because of its good evidence base across disorders and in neurotypical groups.

There is a preliminary session (baseline/relationship building with parent) followed by six intervention sessions (delivered weekly to fortnightly), each with a theme building on techniques and learning from the previous session. The first intervention sessions focus on infant behaviour (with maternal behaviour alluded to only indirectly), the third and fourth sessions address maternal behaviour, and the final two sessions examine more complex chains of social interaction. The set-up of each session is designed to facilitate exploration of these specific targeted themes. These are followed by up to five booster sessions to consolidate learning; the number and content of these booster sessions is agreed between therapist and parent based on progress and development in the six themed sessions. Feasibility and acceptability of the method was shown in an independent case series study[4], and efficacy has been demonstrated with infant siblings of children with ASD[5-6].

© 2021 Whitehouse AJO et al. *JAMA Pediatrics.*

SESSION PLANS

INTRODUCTORY SESSION

**Aim:**<br>**Introduction, rapport building, goal-setting, and baseline measurement.**	**Record video for session 1**<br>Six minute parent-infant interaction: free play with toys

SESSION 1 – "INFANT WATCHING"
Theoretical Focus: Sensitive responding

The parent has an opportunity to observe the focus and choice of activity of their infant without interruption. This encourages her to observe their child closely and to recognize the pace of the infant's exploratory behaviours and to match her own responses accordingly. The parent's experience of watching her infant may also encourage her to think of him or her as a "thinking" being and help her appreciate the potential positive impact of a timely and sensitive response to her child's behaviours.

**Aim:**<br>Observing and naming infant social interactive behaviour	**Record video for session 2**<br>Free play interaction (6 mins)<br>Non-interactive play (2 mins)

SESSION 2: ' SPEAKING FOR THE BABY'
Theoretical Focus: Inference of intentionality

The observations made of the first session are discussed in depth with a focus on the attribution of intentionality to the infant. The purpose is to reinforce parental empathy with the infant's affect state as this forms the basis of a sensitive contingent response. The parent is asked to describe the baby's activities and thoughts speak 'for the child' thus demonstrating awareness of their perspective.

**Aim:**<br>Observing infant interactive behaviour in conjunction with exploratory behaviour	**Record video for session 3**<br>Free play interaction (6 mins)

SESSION 3: 'SENSITIVITY CHAINS'
Theoretical Focus: Synchrony and contingent responsiveness

Building on the concepts introduced in session 2, the parent is encouraged to respond to a range of infant behaviours and match her responses to the behaviour of the infant, thereby increasing synchrony. The identification of sensitivity chains (infant behaviour-maternal response-positive infant response) reinforces the parent's awareness of contingent responsiveness as she demonstrates attunement to her infant's needs.

© 2021 Whitehouse AJO et al. *JAMA Pediatrics.*

**Aim:**<br>Encourage parental contingent responsiveness<br>Particular reinforcement of inter-personal face to face type interactions	**Record video for session 4**<br>Naturalistic setting of a meal time or snack time (20-30 mins)

SESSION 4: SENSITIVITY CHAINS AT MEALTIMES
Theoretical focus: Contingent responsiveness in everyday situations

This session focuses on generalizing the skills addressed in session 3 to an everyday context in a naturalistic setting to help the parent integrate skills such as attunement and synchrony with her infant to every interaction between them.

**Aim:**<br>Generalising contingent responsiveness to a naturalistic setting	**Record video for session 5**<br>Face-to-face 'songs and rhymes' interaction (6 mins)

SESSION 5: 'SHARING FEELINGS'
Theoretical Basis: Affect matching

Session 5 introduces a technique to enhance maternal empathy: inviting the parent to speak as if she herself were the infant (subtly different from describing the baby's ideas in session 2). This is carried out using a video clip of face-to-face interaction to encourage affect matching.

**Aim:**<br>Encourage affect matching and empathy<br>Reinforcement of inter-personal interactions, including eye contact	**Record video for session 6**<br>Free play with toys, to include reading a book together if possible (4 mins)<br>"Funny Sound Game" (2 mins)

SESSION 6: "SHARING TALK"
Theoretical Focus: Communication

In this session the mother is assisted to reflect on more subtle aspects of vocal and non-vocal communication in the context of a structured interaction involving book reading. The aim is to support reciprocal vocalisations in a social context with contingent, attuned responses from the parent.

**Aim:**<br>Encourage vocal communication and social babble<br>Reinforcement of interpersonal interactions, including eye contact	**Record video for booster session**<br>Free play with toys (6 mins)

© 2021 Whitehouse AJO et al. *JAMA Pediatrics.*

SESSIONS 7 – 9: REINFORCEMENT AND BOOSTER SESSIONS; FURTHER MANAGEMENT OF ATYPICALITY

Reinforcement and booster sessions

The aim of these sessions is to reinforce the parent's learning and ensure progress in parent-infant synchrony, attunement and communication as the infant rapidly learns new skills. This will sometimes involve a return to earlier themes e.g. 'infant watching', observation and sensitivity to the infant's particular traits and reinforcing synchronous responses.

Identification of atypicality

The therapist in the *i*-BASIS study will not have been involved in the baseline assessment. However, during the intervention sessions there will have been adequate time for the therapist to identify any evidence of atypicality in the infant within the therapy context. Therapists use a checklist of potential atypicalitiesas an aide memoire at the end of sessions and rate behaviours on a 0-2 rating scale after the introductory session, and then after the 3rd, 6th, 9th and final sessions. *Identified atypicalities are discussed with the parent in terms of the infant's behavioural repertoire without labelling them as prodromal signs*. They are identified as potential barriers to the processes of reciprocity and shared communication and appropriate advice is given to facilitate interaction. The degree of interactional perturbation is likely to vary considerably with each infant and parent. Detailed intervention approaches to address potential atypicalities are described in the full procedural manual; the selection of approaches is tailored to the individual dyad. The therapist adopts a collaborative and exploratory approach with the parent to reduce the impact of these potentially atypical behaviours.

We have considered it important to have an intervention that does not assume atypicality in a group of infant siblings of children with autism spectrum disorder. In cases where a parent and infant have successfully established reciprocal and mutually satisfactory interaction within the 6 intervention sessions or before the end of the booster sessions the final visits can be spaced more or the total number limited by mutual agreement with parent. In this way *i*-BASIS has built-in flexibility to the heterogeneity of development in the intervention group. The generic parental enhancement techniques in VIPP have demonstrated applicability across a range of normative parenting styles; the additional components more specific to prodromal autism can adapt the intervention where children are presenting with differences in development.

© 2021 Whitehouse AJO et al. *JAMA Pediatrics.*

eMethods 2. iBASIS-VIPP Intervention Fidelity Rating Scale

Fidelity Coder: ______________________________

Therapist: ___________________________ Participant ID: ____________________

Feedback Session Date: _________________ Play Session Date: ________________

Intervention session number (circle one): 1 2 3 4 5 6 Booster 7 8 9 10 11 12

Material to accompany Rating Scale:

- Video of Play Session
- Video/audio recording of Feedback Session
- Copy of Script
- Any relevant info from therapist's Session record for (i) Play or (ii) Feedback Session

A. General Therapeutic Procedures

Review of Previous Session including discussion of Practice Tasks (Session 2 onwards)

0 The therapist did not review previous session with parent, including a review of practice tasks.

1 The therapist provides a summary of previous session content and asks how the parent got on with the practice tasks, including whether they had the opportunity to watch the video during the week.

Introduction of Current Session Theme

0 The therapist did not introduce the session theme before reviewing the video.

1 The therapist effectively introduces the appropriate session theme before reviewing the video.

Session Theme Illustrated during Video Review

0 The therapist did not illustrate the relevant theme or made comments that were vague or not related to the session theme.

1 The therapist effectively illustrates the session theme, including commenting on the appropriate aspects of the infant's behaviour.

Eliciting Parent Feedback

0 Little or no attempt by the therapist to elicit feedback to determine if the parent had understood the strategies and techniques being utilised in the session. Did not ask enough questions to be sure the parent understood the session theme or to ascertain the parent's reactions to the session.

1 The therapist elicited feedback from the parent to determine the parent's observation and understanding of session theme.

Response to Parents Focus

0 No attempt to recognise or respond to parents focus.

1 Recognised and responded appropriately to parents focus throughout the session.

© 2021 Whitehouse AJO et al. *JAMA Pediatrics.*

Managing the Feedback

0 Little or no structure to the feedback time *or* pacing too slow or too fast, or was inflexible or not adapted to the task in hand.

1 The therapist structured the feedback well, so there was a clear beginning, middle and closing of the feedback. Peripheral and unproductive digressions were either very uncommon, or handled well by the therapist.

Pacing

0 The therapist pacing and timing was not appropriate to the parents.

1 The therapist pacing and timing was appropriate to the parents.

Summary and Setting of Practice Tasks

0 The therapist did not provide a summary for the session, and failed to set practice tasks, *or* set tasks that were vague, incomplete, or unilaterally determined.

1 A summary of the main observations from the session was provided verbally by the therapist and on paper. There was setting of practice tasks that arose directly from the session and was jointly agreed.

B. Interpersonal Effectiveness

Sensitivity Skills

0 The therapist failed to reflect or rephrase what the parent explicitly said or showed problems responding to implicit or subtle communication.

1 The therapist generally seemed to grasp the parent's meaning as reflected by both what the parent explicitly said and what the parent communicated more subtly.

Validation and Positive Feedback

0 The therapist missed opportunities to praise parental achievements.

1 The therapist recognised and appropriately praised parental achievements

C. Specific Criteria

1 The therapist made a positive comment about the interaction or the play situation within the first 10-15 seconds of the video.

Yes / No

2 The therapist made at least one compliment during the feedback; for example, an appropriate and timely parental response (i.e., when the parent responds promptly to cues from her infant).

Yes / No

3 The therapist makes a comment using "Speaking for the baby" at least every 30 seconds.

Yes / No

4 *From session 3 onwards only:* Therapist uses 2 sensitivity chains appropriately

Yes / No / NA

© 2021 Whitehouse AJO et al. *JAMA Pediatrics.*

5 *From session 3 onwards only:* Therapist uses 1 corrective message appropriately
Yes / No / NA

D. Use of Booster Sessions

0 No clear aim established for the booster session.
1. A clear aim to the booster session was established. This may be from previous discussion with parent about which Session theme(s) they would like to repeat, or address any current issues.

Yes / No / NA

E. Deviation from the Manual
Were there any significant unusual factors in this session that you feel justified the therapist's departure from the manual?

Yes / No / NA

Please explain below:
____
____
____
____
____
____
____
____
____

F. Materials
For standardisation of the first and final session: the core play materials were available to the parent and infant's toys removed/minimised.
Yes / No / NA

G. Room Environment
Were distractions minimised; for example, presence of other children, family members, etc or external noises such as TV, radios?
Yes / No

H. Video and Sound
Was the quality of the video and sound adequate? For example, could the facial expressions and interactions between the parent and infant be viewed? Were the sounds made by infant and parent audible?
Yes / No

© 2021 Whitehouse AJO et al. *JAMA Pediatrics.*

I. Quality Time

Was there adequate opportunity for parent/therapist discussion?

Yes / No

© 2021 Whitehouse AJO et al. *JAMA Pediatrics.*

eMethods 3. Additional Detail on Methods

Study design
The study was a two-site (Perth and Melbourne, Australia), single-blind RCT, with participants randomly allocated to receive either iBASIS-VIPP therapy (iBASIS-VIPP group) or usual community care (usual care group) over a 5-month period. Developmental assessments were conducted at: (1) baseline, (2) 6-months following baseline (treatment endpoint), (3) 12-months post-baseline, and (4) 24 months post-baseline. The study was approved by the Child and Adolescent Health Service Ethics Committee (2016008EP, June 8th, 2016), and each family provided written informed consent. Trial registration is available at: ANZCTR12616000819426.

Participants
Trial recruitment and participant eligibility has been reported previously[47]. Infants were identified via referral from community clinicians. Families were invited into the trial if: (a) the infant was in the age range of 9-months to 14-months, 31 days (corrected for prematurity) at eligibility screening; (b) the infant displayed at least three of the five key 'ASD risk' behaviours (spontaneous eye contact, proto-declarative pointing, social gestures, imitation, and response to name) on the Social Attention and Communication Surveillance-Revised (SACS-R) 12-month checklist,[7] and (c) the primary caregiver spoke sufficient English to participate fully in therapy sessions. Exclusion criteria were: (a) diagnosed comorbidity known to affect infant neurological and developmental abilities (including birth <32 weeks' gestation); and/or (b) the family did not intend to remain living in the local area for the trial duration.

Social Attention and Communication Surveillance-Revised
The Social Attention and Communication Surveillance – Revised (SACS-R) tool is a series of checklists designed to monitor key behavioural markers of social and communication differences at 12, 18, and 24 months.[7] The checklists were developed to be direct observational tools administered by health professionals. 'Key' social-communicative behaviours assessed by the SACS-R include the presence and/or quality of a child's eye contact, pointing, response to their name, imitation, social gestures, showing objects, and pretend play. Additionally, difference in other behaviours that are frequently associated with developmental conditions can be identified using SACS-R, including social smiling, joint attention, use and understanding of language, parallel play, interest in other children, and any loss of skills. Children considered to be at 'high likelihood' for ASD demonstrate a pattern of 'atypical' behaviour on at least three 'key' items at 12, 18 and/or 24 months. The original SACS has excellent Positive Predictive Value (PPV; 81 %) for identifying ASD between 12 and 24 months of age, and excellent sensitivity (84 %), and specificity (99%),[8] with similar psychometric properties found for the SACS-R (PPV: 82–83 %; Negative Predictive Value: 98–99 %; sensitivity: 77–82 %; specificity: 99–99.5 %).[9-10]

At the Melbourne site, the SACS-R assessments were carried out using the 12-month-old SACS-R checklist by MCH nurses within the community as part of their routine practice. Training of the MCH nurses was led by one of the study authors (Dr Josephine Barbaro), and met competency requirements prior to using the SACS-R in their community practice. The Perth site used a modified approach to SACS-R administration. Once referrals were received by the Child Development Service (via health or education professionals or via parent/caregiver referral), parent(s)/caregiver(s) of infants in the relevant age range for this study were telephoned. A Speech and Language Therapist (Michelle Renton) trained on

© 2021 Whitehouse AJO et al. *JAMA Pediatrics.*

SACS-R administration interviewed the parent(s)/caregiver(s), asking about the behaviours contained in the 12 month SACS-R checklist; the five key items that determined a child's 'likelihood' for ASD are presented below:

- *Eye contact:* Has the child spontaneously made eye contact with you during the session? If not, interact with the child to elicit eye contact. Does he/she make eye contact with you?
- *Pointing:* Get a teddy bear, show it to the child and say 'This is teddy'. Then put the bear across the room (where the child can see it) and say, 'Where's teddy?' Does the child point to the bear and look at your face?
- *Gestures:* Elicit the social routine of waving bye-bye (e.g. pretend to leave room and wave bye-bye to the child). Does he/she wave back?
- *Imitation:* Get the child's attention. Use a brush/comb on your hair. Give it to the child and say 'your turn'. Does he/she imitate you?
- *Response to name:* Call the child's name. Does he/she turn to look at you? (Make sure child is not already looking at you)

An answer of 'no' to 3, 4 or 5 of the above items met the SACS-R related inclusion criterion for trial entry.

Allocation and masking

After eligibility was determined, participants were randomly assigned (1:1 via computer algorithm) participants to either iBASIS-VIPP or usual community care groups. Randomisation was by minimization, stratified by site (Perth, Melbourne), infant sex (male, female), number of SACS-R risk behaviours (3, 4, 5 key items endorsed) and age band at recruitment (9-11 months, 12-14 months, corrected for prematurity), with assignment determined by a 'biased coin' of probability 0.7. Given the parent-mediated nature of the intervention, group allocation could not be masked from families. Research staff conducting the baseline and follow-up assessments were housed separately to staff conducting the therapy, and all assessments were administered and coded blind to other information including group allocation, with the exception of parent-report questionnaires.

Intervention

The intervention was iBASIS-VIPP and the comparator was community therapy as usual. The iBASIS-VIPP intervention involves up to 12 individual sessions (one introductory, six core, and up to five booster sessions) delivered in family homes by a therapist (here, speech and language therapist or clinical psychologist) at fortnightly intervals over a 5-month period. In the current study, three booster sessions were offered to all participants, making 10 sessions in total. The primary caregiver is asked to participate in all therapy sessions, during which interactions between the caregiver and infant are videotaped, providing the basis for video feedback discussion. Core aspects of iBASIS-VIPP include: (a) a focus on the communicative aspects of each parent-infant dyad; (b) viewing videotaped interaction excerpts providing positive examples of a sensitive interaction style; and (c) a trained therapist framing observations, assisting with self-reflection, and focusing on behavioural change. Parents were asked to undertake 15-minutes daily home practice in interacting with their infant using the newly learned skills. Two therapists at each site were trained to protocol fidelity by experts from the iBASIS and VIPP development teams. Any adverse effects of the intervention were recorded by the therapist at the end of each session based on clinical observation and parent report.

Therapist fidelity to the manual was assessed on 40 sessions (35 participants) randomly selected to balance timepoint and therapist. These were double-coded with a 21-item pass/fail

© 2021 Whitehouse AJO et al. *JAMA Pediatrics.*

measure of therapeutic skills and specific iBASIS-VIPP strategy by the originating UK iBASIS-VIPP team (CT, JG, VS). (See section titled: iBASIS-VIPP intervention fidelity rating scale). The mean fidelity score was 20·5 passed items/session (range=18–21); only one of 40 sessions was below the 80% fidelity threshold.[11]

Infants receiving community therapy were not excluded from the iBASIS-VIPP group. Parents in both treatment groups completed a weekly diary, recording all contact with health professionals between baseline and endpoint assessments.

Measures

Baseline and follow-up assessments took place in a research setting at the Telethon Kids Institute (Perth) and La Trobe University (Melbourne). Baseline assessments typically took place at a mean of 2.53 weeks (SD = 1.50; range 0.29–8.71) after eligibility screening. While this time period could not be achieved for one participant (assessment took place at 8.71 weeks) for logistical reasons (infant illness), age eligibility requirements for this infant were still met. Treatment endpoint assessments took place at a mean of 6 22 months (SD = 0.60; range 4.4–9.2) post-baseline. The 12-month post-baseline assessment took place at a mean of 12.5 months (SD = 0.71, 11.3-17.0) after the baseline assessment, and the 24-month post-baseline assessment took place at a mean of 24.5 months (SD = 0.62; range: 23.7-28.3 months) after the baseline assessment.

Primary outcomes

The primary outcome of the trial was autism prodromal symptoms, which was assessed by two conceptually analogous measures. The measure used at the two infancy assessments (baseline, treatment endpoint) was the *Autism Observation Scale for Infants* (AOSI). The AOSI is a semi-structured observation assessment that measures early behavioural signs associated with ASD,[12] including response to name, social reciprocity and imitation. An assessor trained to fidelity codes observed behaviours as 0, 1, 2 or 3, with a higher score indicating a greater level of ASD-like atypicality. The 19-item version of the AOSI was used, which includes 16 scoring items (Total Score range: 0-38). A Total Score of ≥ 9 at 12 months has been used to indicate clinical levels of developmental difference.[12] The AOSI Total Score has strong inter-rater reliability (≥ .92).[12] Studies of infant siblings of children with ASD have reported that AOSI scores at 14 months of age have moderate accuracy in predicting later ASD diagnostic status at 36 months.[13] However, there still remains a moderate degree of false-positives in ASD screening and assessment measures during infancy, and there are no data on AOSI reliability and validity currently available for clinically-indicated infants. The AOSI was administered by one trained researcher at each site. While steps were taken to ensure the assessing researcher would be blind to participant group, pre-empting that parents might plausibly inadvertently disclose their group assignment to the administering researcher at the immediate outcome assessment, we guaranteed blindness on this primary trial outcome measure having each assessment filmed, and then scored by the researcher at the alternate site. A random subset (20%) of AOSI recordings (baseline, treatment endpoint) was also coded by the trained local site researcher for a formal within-trial reliability evaluation. There was good inter-rater agreement on the AOSI Total Score; single measures intraclass correlations (ICC), two-way mixed effects model for absolute agreement =.78.

The *Autism Diagnostic Observation Schedule - 2nd Edition* (ADOS-2),[14] was administered at the two toddler assessments (12-months post baseline, 24-months post-baseline) as a measure of autism prodromal symptoms. The ADOS-2 is a semi-structured assessment developed to elicit behaviours relevant to an ASD diagnosis in toddlers aged 12-30 months (Toddler

© 2021 Whitehouse AJO et al. *JAMA Pediatrics.*

Module) or 31 months and above (Modules 1 to 4), and is used internationally as part of ASD diagnostic assessment. The ADOS-2 Toddler module[15] was administered at the assessment 12-month post-baseline, with the Total Algorithm Score used as the outcome variable. One of two ADOS-2 modules was administered at the assessment 24 months post-baseline, depending on whether children had minimal language (Module 1) or phrase-level language (Module 2). To standardise scores across the modules, the ADOS Calibrated Severity Score (CSS)[16] was derived from the Total Algorithm Score of each module, and used as the outcome variable, enabling direct comparisons across the different developmentally staged ADOS-2 Modules 1 and 2. CSS range from 1 to 10, with higher scores representing greater severity of autistic symptoms.

All ADOS assessments were conducted and scored by researchers who had completed research-level training in administration and coding, including on the core set (Modules 1-4) and the specialist Toddler Module. Across the trial data collection period, ADOS-2 review meetings were held approximately monthly (up to 10 per year, mid 2017 to early 2020). These were coordinated by experienced ADOS-2 trainers and served two purposes; 1) to ensure maintenance of administration and scoring standards through regular review of trial assessment footage, and 2) to facilitate formal within-trial appraisal of scoring reliability. Following practices reported by ADOS-2 developers[14] and implemented in other autism trials,[35] the item-level codes of each researcher in attendance at a review meeting were recorded, and discrepancies were discussed to reach consensus. Review meetings covered the three ADOS-2 Modules relevant to the trial with a total of 29 tapes (16% of trial assessments across the 12-month and 24-month post-baseline assessments) reviewed in this way; 13 Toddler Module, 7 Module 1 and 9 Module 2 tapes.

All ADOS-2 assessments were scored from video footage, with tapes assigned such that researchers never scored an assessment that themselves had administered. This was for consistency and to ensure we would be able to guarantee blindness to treatment group on this primary outcome measure (i.e., pre-empting that parents might inadvertently disclose their infants' group to the administering researcher at an assessment visit). Hence, while eight researchers involved in administering trial ADOS-2 assessments participated in review meetings, the ADOS-2 Toddler Module assessments were ultimately coded by just three individuals (JS, LC, SP), the Module 1 assessments by four individuals (KH, LC, JS, KV), and the Module 2 assessments also by four (LC, JS, CG SP). Item-level data were recorded for a total of 69 coding protocols completed by these individuals, across the 29 reviewed tapes.

The three researchers who coded ADOS-2 Toddler Module assessments (22 coding protocols across 13 tapes) averaged 85% raw agreement with consensus, on item-level scoring (range 84-85% across individuals). Inter-rater reliability on ADOS-2 Toddler Module Total Algorithm Scores – computed on the basis of item-level scores for each researcher vs. from the consensus codes – was excellent (intraclass correlations r = .91).

The four researchers who coded ADOS-2 Module 1 assessments (13 coding protocols across 7 tapes) averaged 89% raw agreement with consensus, on item-level scoring (range 85-92% across individuals). The four individuals who coded ADOS-2 Module 2 assessments (26 ratings across 9 tapes) averaged 83% raw item-level agreement (range 80-88% across individuals). Inter-rater reliability on Total Algorithm Scores – computed from item-level scores for each researcher vs. from the consensus codes – was excellent for both Module 1 (intraclass correlations r = .95) and Module 2 (intraclass correlations r = .92). Inter-rater

© 2021 Whitehouse AJO et al. *JAMA Pediatrics.*

reliability on the CSS – derived from Total Algorithm Scores, to permit direct comparability across the various ADOS-2 Modules – was also computed (again for each researcher vs. from consensus coding) and found to be very high (intraclass correlations r = .88).

Secondary outcomes.

A secondary outcome for this study was a *clinical ASD diagnosis*. Following the completion of all data collection, clinicians experienced in ASD diagnosis (AC, LM) reviewed all clinical information collected at the 12- and 24 months post-baseline assessments, including observational measures of ASD symptomatology (ADOS-2; record forms, videotapes) and development (MSEL; record forms), and parent-report measures of adaptive functioning (VABS-III) and language development (MCDI). Following a pre-specified protocol (see section titled: Protocol for clinical best estimate of case diagnosis), the clinicians provided a consensus diagnosis according to the following categories: ASD, a diagnosis of ASD consistent with DSM-5 can be made with high confidence); Possible ASD, presence of some autistic traits but not sufficient to provide a diagnosis of ASD with high confidence; other developmental concerns, the presence of developmental concerns that are not indicative of ASD; No developmental concerns, development is within normal limits and the child does not require further developmental monitoring.

A secondary outcome was the *Manchester Assessment of Caregiver-Infant interaction (MACI)*,1[5] a global rating measure of characteristics of parent-infant interactions. The MACI is blind video-coded from a 6-minute play session between parent/caregiver and infant on a range of 7-point subscales (range: 1-7). MACI was specifically designed to measure key interactional aspects of early social development, and elements of parent-infant interactions that have previously been associated with a later diagnosis of ASD[18]. Four scales showing the greatest predictive validity[18] were pre-defined as the scales of interest for the current study: Caregiver Sensitive Responsiveness, Caregiver Nondirectiveness, Infant Attentiveness, and Infant Positive Affect. The infant version of the MACI was used for the two infancy assessments and the toddler version of the MACI was used for the two toddler assessments. Within-trial independent double coding (by MACI developer, MWW) which was conducted blind to treatment group, showed good to high inter-rater agreement based on single measures intraclass correlation (two-way mixed effects model) for both the infant (.67-.80; 15% of clips, randomly selected from baseline and treatment endpoint assessments) and toddler (.70-.93; 16% of clips, randomly selected from the 12-months post-baseline and 24-months post-baseline assessments) versions of the MACI (all $p < 0.001$).

Additional secondary outcomes measured different aspects of child development. *The Mullen Scales of Early Learning (MSEL)*[19] is a researcher-administered developmental assessment of early motor and cognitive development from 0-68 months. We pre-defined the subscales of interest for this study as Receptive Language, Expressive Language, Visual Reception, and Fine Motor. Due to the presence of floor effects, raw scores were used in the current study, as is common for children with ASD.[20]

The *Vineland Adaptive Behavior Scales–2nd edition (VABS-2)*[21] is a caregiver-report measure of functional skills relevant for everyday living. The Communication and Socialization subscales were pre-defined as the subscales of interest, using age-normed standard scores (M=100, SD=15).

The *MacArthur-Bates Communicative Development Inventory (MCDI)*[22] is a caregiver-report measure providing indices of early vocabulary and gesture use. At the infancy assessments,

© 2021 Whitehouse AJO et al. *JAMA Pediatrics.*

we used the MCDI Words and Gestures (WG) form was used, where caregivers endorse the number of words the child 'understands' or both 'understands and says' among an inventory (maximum = 396 words) spanning different semantic categories. At the toddler assessments, we used the MCDI Words and Sentences (WS) form (maximum = 680 words), with slight modification so that caregivers again endorsed words the child 'understands' or 'understands and says'. Further, we appended the section pertaining to gestures to the MCDI WS. Outcomes of interest at each assessment point were therefore an Expressive Vocabulary Count (total of all items endorsed 'understands and says'), a Receptive Vocabulary Count (combined total of all items endorsed 'understands' or 'understands and says') and a Total Gestures score.

The *Parenting Sense of Competence (PSOC) scale*[23] is a measure of the caregiver's own sense of parenting efficacy, which is rated on a 6-point scale (range: 1-6). Endorsed PSOC items are summed to yield three subscales: Satisfaction (range: 6-36), Efficacy (range: 5-30) and Interest (range: 3-18).

© 2021 Whitehouse AJO et al. *JAMA Pediatrics.*

eMethods 4. Protocol for Clinical Best Estimate Judgment of Case Diagnosis

Developed by: Andrew Whitehouse, Kandice Varcin, Sarah Pillar, Kristelle Hudry, Jonathan Green and John Wray

Date: 8th January 2020 (Version 2)

Brief description of trial aims and hypotheses:
This study will evaluate a parent-mediated, behavioural intervention (iBASIS-VIPP) in 9-14-month old infants identified as showing 'risk behaviours' for Autism Spectrum Disorder (ASD). In this randomised-controlled trial (RCT), we aim to determine whether iBASIS-VIPP in addition to usual community care, applied for a 5-month period, can reduce ASD symptom severity immediately post-treatment (6-months post-baseline) and at 12- and 24-months post baseline, compared with usual community care alone.

The primary outcome for the follow-up phase of the trial is scores on the Autism Diagnostic Observation Schedule – 2nd edition (ADOS-2) at 6, 12 and 24 months' post-baseline, and the secondary outcomes are a range of developmental outcomes at these time points. These analyses are pre-registered on the ANZCTR (ACTRN12616000819426).

A further outcome of interest is to determine the ASD diagnostic status of the infants at the final assessment, and understand whether this differs between treatment groups. This determination will be made following assessment at 24 months' post baseline, when we anticipate a mean age of approximately 3 years, and will draw on any/all available data for children collected at their 12- and 24-month post baseline assessments.

Below is a description of how the best estimate clinical judgment will be made.

Process

Clinical team

The clinical team will comprise the same two allied health professionals- a Clinical Psychologist and Speech Pathologist- both of whom have extensive experience in the diagnosis of young children with ASD. Neither member of the clinical team will have previously observed the child for whom a clinical judgment is being made.

Clinical data available

The clinical team will be provided with a case file of each child who attended the Follow-up 2 and/or 3 appointment. The only exclusion criterion will be if the child has been diagnosed with a genetic syndrome that excludes the diagnosis of ASD (e.g., Rett Syndrome). The order of case file review will be allocated randomly by the Melbourne site.

The Clinical Best Estimate will largely be made on the basis of the following data, which will be made available in the case file on the day it is allocated for review (where the child attended both Follow-up 2 and Follow-up 3 and it is available):

- Follow-up 2 & 3 Autism Diagnostic Observation Schedule – 2nd edition (ADOS) video
- Follow-up 2 & 3 ADOS scored protocol
- Follow-up 2 & 3 Visit Report*
- Follow-up 2 & 3 Parent Child Interaction video

© 2021 Whitehouse AJO et al. *JAMA Pediatrics.*

- Follow-up 3 Mullen Scales of Early Learning

*The Visit Report describes observations made by the Assessor across the assessment session, with a particular focus on observations made during the ADOS-2. Visit Reports also included a best estimate clinical judgement made by the Assessor about the diagnostic status of the child according to four options: No developmental concerns, possible ASD, definite ASD; other development concerns (with a description of these).

The clinicians will also be aware of the additional data collected about the child across the timepoints, and will have access to these documents upon request:

- Family History Questionnaire (FHQ)
- Social Communication Questionnaire (SCQ) - Follow-up 3 only
- The MacArthur-Bates Communicative Development Inventory (MCDI)
- Vineland Adaptive Behaviour Scales (VABS-II)
- Early Childhood Behaviour Questionnaire (ECBQ)
- Infant-Toddler Social and Emotional Assessment (ITSEA)
- Preschool Children Quality of Life (TAPQOL)

Consensus meeting

A consensus meeting will be attended by the two members of the clinical team to discuss each case file. The aim of the consensus will be to determine whether, based on the clinical data available in the case file, the child meets DSM-5 criteria for Autism Spectrum Disorder.

Prior to the meeting, each member of the clinical team will have the opportunity to examine each child's clinical file. During the meeting, the team will be asked to determine a consensus view about which of the three criteria in the 'social communication and interaction' domain are met / not met / or partially met, and which (if any) of the four criteria for the 'restricted and repetitive behaviours and interests' domain are met / not met / or partially met. The clinical team will not be asked to provide DSM-5 severity scores for criteria.

The clinical team will be asked to provide two pieces of information for each child:

1. An indication of which DSM-5 criteria for ASD are met / not met / or partially met, by a given child; and
2. A best estimate clinical judgment, according to four options:
    i. Definite ASD – a diagnosis of ASD can be made with high confidence.
    ii. Possible ASD – presence of some autistic traits, but not sufficient to provide a diagnosis of ASD with high confidence.
    iii. No developmental concerns – development is within normal limits and the child does not require further developmental monitoring.
    iv. Other developmental concern (specify) – the presence of developmental concerns, which are not indicative of ASD.

Where a consensus decision cannot be achieved, the best estimate clinical judgment will be made by the Clinical Psychologist. The rationale for this decision is that Clinical Psychologists received more intensive clinical training that Speech Pathologists with differential diagnosis.

© 2021 Whitehouse AJO et al. *JAMA Pediatrics.*

This information will be provided on all children who attended the Follow-up 2 and/or 3 sessions. In situations where the child attended Follow-up 2 but not Follow-up 3 assessments, the consensus team will still be asked to generate this information. However, interpretation of these consensus decisions will be with an acknowledgment that diagnostic decision making is more accurate with older children.

© 2021 Whitehouse AJO et al. *JAMA Pediatrics.*

eTable 1. Usual Community Care Received by Participants in the iBASIS-VIPP and Usual Care Groups That Was Not Associated With the Clinical Trial

Participants in both the iBASIS-VIPP and usual care groups were able to receive community intervention during the study treatment period. Parents in both groups reported all contact with health professionals between assessments. This information is summarised in eTable 1.

**12-month follow-up assessment (i.e, at 24 months of age)[a]**						
	Total sample		Perth site only		Melbourne site only	
	iBASIS-VIPP (n = 46)	Usual care (n = 42)	iBASIS-VIPP (n = 29)	Usual care (n = 30)	iBASIS-VIPP (n = 17)	Usual care (n = 12)
Any therapy received – n %	21 (45.7%)	29 (69%)	13 (44.8%)	23 (76.7%)	8 (47.1%)	6 (50%)
Speech and Language Therapy received– n (%)	15 (32%)	20 (47.6%	11 (37.9%)	16 (53.3%)	4 (23.5%)	4 (33.3%)
Occupational Therapy received– n (%)	7 (15.2%)	6 (14.3%)	6 (20.7%)	4 (13.3%)	1 (5.9%)	2 (16.7%)
Physical Therapy received – n (%)	4 (8.7%)	7 16.7%)	3 (10.3%)	5 (16.7%)	1 (5.9%)	2 (16.7%)
Psychology received – n (%)	3 (6.5%)	0 (-)	0 (-)	0 (-)	3 (17.6%)	0 (-)
Comprehensive autism intervention received – n (%)	2 (4.3%)	3 (7.1%)	0 (-)	2 (6.7%)	2 (11.8%)	1 (8.3%)
**24-month follow-up assessment (i.e, at 36 months of age)[b]**						
	Total sample		Perth site only		Melbourne site only	
	iBASIS-VIPP (n = 45)	Usual care (n = 43)	iBASIS-VIPP (n = 29)	Usual care (n = 30)	iBASIS-VIPP (n = 16)	Usual care (n = 13)
Any therapy received – n %	19 (42.2%)	26 (60.4%)	10 (34.4%)	19 (63.3%)	9 (56.3%)	7 (43.8%)
Speech and Language Therapy received– n (%)	10 (22.2%)	19 (44.2%)	6 (20.7%)	17 (56.7%)	4 (25%)	2 (12.5)
Occupational Therapy received– n (%)	8 (17.8%)	10 (23.6%)	4 (13.8%)	6 (20%)	4 (25%)	4 (25%)
Physical Therapy received – n (%)	4 (8.9%)	6 (14.0%)	3 (10.3%)	4 (13.3%)	1 (6.3%)	2 (12.5%)
Psychology received – n (%)	3 (6.7%)	1 (2.3%)	0 (-)	0 (-)	3 (18.8%)	1 (6.3%)
Comprehensive autism intervention received – n (%)[c]	4 (8.9%)	3 (7.0%)	0 (-)	2 (6.7%)	4 (25%)	1 (6.3%)

[a]Therapy information is missing for n = 5 participants who were assessed at the 12-month post-baseline assessment (iBASIS-VIPP group: n = 1; usual care group: n = 4)
[b]Therapy information is missing for n = 1 participant who was assessed at the 24-month post-baseline assessment (usual care group: n = 1).
[c]Comprehensive autism interventions included Early Start Denver Model, Applied Behavioural Analysis, and DIR/Floortime.

© 2021 Whitehouse AJO et al. *JAMA Pediatrics.*

eTable 2. Consensus Diagnostic Groups by *DSM-5* Diagnostic Criteria

ASD diagnostic criteria endorsed by consensus diagnostic groups

	Typical development (n = 13)	Atypical (n = 64)	ASD (n = 12)
	n (%)	n (%)	n (%)
**Criterion A1**	0 (-)	13 (20.3)	12 (100)
Deficits in social-emotional reciprocity			
**Criterion A2**	0 (-)	18 (28.1)	12 (100)
Deficits in nonverbal communicative behaviors used for social interaction			
**Criterion A3**	0 (-)	17 (26.6)	12 (100)
Deficits in developing, maintaining, and understanding relationships			
**Criterion B1**	0 (-)	11 (17.2)	10 (83.3)
Stereotyped or repetitive motor movements, use of objects, or speech			
**Criterion B2**	0 (-)	1 (1.6)	3 (25)
Insistence on sameness, inflexible adherence to routines, or ritualized behavior			
**Criterion B3**	0 (-)	2 (3.1)	3 (25)
Highly restricted, fixated interests that are abnormal in intensity or focus			
**Criterion B4**	0 (-)	0 (-)	10 (83.3)
Hyper- or hyporeactivity sensory input or unusual sensory interests			
	Median (range)	Median (range)	Median (range)
Number of social communication impairment items endorsed (i.e., A1-A3)	0 (0 - 0)	0 (0 - 3)	3 (3 - 3)
Number of restricted, repetitive patterns of behavior endorsed (i.e., B1-B4)	0 (0 - 0)	0 (0 - 2)	2 (2 – 3)
Number of items endorsed in total	0 (0 - 0)	0 (0 – 4)	5 (5 - 6)

© 2021 Whitehouse AJO et al. *JAMA Pediatrics.*

eTable 3. Outcome Data for Children Meeting *DSM-5* Criteria for Autism Spectrum Disorder

Data collected at the 24-month post-baseline assessment for children meeting criteria for ASD.

ID[a]	Group	Chronological age at assessment (months)	ADOS-2 (Module, Total Score, Calibrated Severity Score)	Mullen Scales of Early Learning. (Raw scores)				Vineland Adaptive Behaviour Scales- 2nd Edition	
001	Usual care	37.84	Module 2; 19; 10	33	31	32	29	Questionnaire not returned	Questionnaire not returned
002	Usual care	35.42	Module 2; 26; 10	Unable to complete	Unable to complete	Unable to complete	Unable to complete	112	94
003	Usual care	35.48	Module 1; 19; 8	29	30	24	21	84	91
004	iBASIS-VIPP	36.6	Module 1; 23; 8	14	17	21	26	63	65
005	Usual care	38.8	Module 1; 22; 10	10	22	28	28	72	78
006	Usual care	37.16	Module 1; 26; 10	14	17	28	23	79	67
007	iBASIS-VIPP	39.29	Module 2; 13; 7	30	34	33	32	65	65
008	Usual care	36.4	Module 2; 15; 8	41	32	40	34	105	108
009	Usual care	37.29	Module 1; 10; 5	26	21	27	25	81	81
010	Usual care	39.09	Module 1; 25; 10	11	9	19	21	65	57
011	Usual care	36.17	Module 1; 22; 7	9	7	13	16	59	49
012	iBASIS-VIPP	38.11	Module 1; 21; 7	14	12	20	22	70	76

[a]Dummy ID for publication

ADOS-2: Autism Diagnostic Observation Schedule – 2nd Edition

© 2021 Whitehouse AJO et al. *JAMA Pediatrics.*

eTable 4. Primary and Secondary Outcome Data Across Assessment Points by Site

	Usual care group				iBASIS-VIPP group			
	Baseline	Treatment endpoint	12-month post-baseline	24-month post-baseline	Baseline	Treatment endpoint	12-month post-baseline	24-month post-baseline
AOSI raw score								
Perth	9.47 (4.16)<br>n = 34	9.38 (5.27)<br>n = 32	-	-	9 (3.7)<br>n = 32	8.84 (4.13)<br>n = 31	-	-
Melbourne	8.89 (5.21)<br>n = 19	9.86 (4.7)<br>n = 14	-	-	11 (3.9)<br>n = 19	9.65 (4.76)<br>n = 17	-	-
ADOS-2								
Perth	-	-	10.65 (5.92) [a]<br>n = 31	5.68 (2.84) [b]<br>n = 31	-	-	8.33 (5.59)<br>n = 30 [a]	5.17 (2.54)<br>n = 29 [b]
Melbourne	-	-	11.86 (7.42) [a]<br>n = 14 [a]	5.69 (2.69) [b]<br>n = 13 [b]	-	-	11.29 (6.38)<br>n = 17[a]	5.38 (1.78)<br>n = 16 [b]
MACI Caregiver Nondirectiveness								
Perth	4.24 (1.46)<br>n = 34	4.58 (1.39)<br>n = 33	4.61 (1.58)<br>n = 31	4.83 (1.36)<br>n = 29	4.25 (1.55)<br>n = 32	4.88 (1.26)<br>n = 32	5.07 (1.15)<br>n = 28	5.07 (1.41)<br>n = 29
Melbourne	3.84 (1.61)<br>n = 19	4.93 (1.49)<br>n = 14	4.55 (1.29)<br>n = 11	3.82 (1.4)<br>n = 11	4.16 (1.83)<br>n = 19	4.76 (1.09)<br>n = 17	4.59 (1.87)<br>n = 17	4.57 (1.28)<br>n = 14
MACI Sensitive Responding								
Perth	4.62 (1.3)<br>n = 34	4.85 (1.03)<br>n = 33	4.65 (1.23)<br>n = 31	4.79 (1.18)<br>n = 29	4.38 (1.58)<br>n = 32	5.12 (1.01)<br>n = 32	5 (1.09)<br>n = 28	4.93 (1.41)<br>n = 29
Melbourne	3.68 (1.49)<br>n = 19	4.71 (1.14)<br>n = 14	3.91 (1.14)<br>n = 11	4.18 (1.08)<br>n = 11	4.05 (1.35)<br>n = 19	4.88 (0.7)<br>n = 17	4.35 (1.5)<br>n = 17	4.5 (1.56)<br>n = 14
MACI Infant Attentiveness								
Perth	4.09 (1.29)<br>n = 34	4.7 (1.1)<br>n = 33	4.32 (1.33)<br>n = 31	5.24 (1.15)<br>n = 29	3.94 (1.27)<br>n = 32	4.5 (1.08)<br>n = 32	4.89 (1.03)<br>n = 28	5.07 (1.22)<br>n = 29

© 2021 Whitehouse AJO et al. *JAMA Pediatrics.*

Melbourne	3.95 (1.51)<br>n = 19	4.71 (0.99)<br>n = 14	3.82 (0.98)<br>n = 11	4.91 (0.94)<br>n = 11	3.68 (1.11)<br>n = 19	4.29 (1.31)<br>n = 17	4.12 (1.17)<br>n = 17	4.93 (1.07)<br>n = 14
MACI Infant Positive Affect								
Perth	3.79 (1.59)<br>n = 34	4.21 (1.19)<br>n = 33	3.26 (1.9)<br>n = 31	4.31 (2.05)<br>n = 29	3.34 (1.47)<br>n = 32	3.75 (1.5)<br>n = 32	3.46 (2.08)<br>n = 28	4.41 (1.84)<br>n = 29
Melbourne	3 (1.86)<br>n = 19	4.86 (1.56)<br>n = 14	3.09 (2.02)<br>n = 11	4.18 (1.78)<br>n = 11	3.26 (1.59)<br>n = 19	3.59 (1.66)<br>n = 17	2.71 (1.86)<br>n = 17	3.21 (2.01)<br>n = 14
MSEL Expressive Language raw score								
Perth	9.12 (2.09)<br>n = 34	15.03 (3.25)<br>n = 33	20.23 (5.06)<br>n = 31	30.53 (5.46)<br>n = 30	9.31 (1.93)<br>n = 32	14.78 (3.18)<br>n = 32	20.17 (4.62)<br>n = 30	30.03 (6.71)<br>n = 29
Melbourne	10.32 (3.07)<br>n = 19	14.8 (4.3)<br>n = 15	18.21 (6.02)<br>n = 14	26.85 (10.15)<br>n = 13	10.84 (2.67)<br>n = 19	16.41 (3.64)<br>n = 17	22.76 (6.84)<br>n = 17	32.62 (8.64)<br>n = 16
MSEL Receptive Language raw score								
Perth	10.38 (2.66)<br>n = 34	15.03 (3.79)<br>n = 33	21.58 (5.53)<br>n = 31	30.2 (6.74)<br>n = 3	9.59 (2.35)<br>n = 32	15.38 (4.43)<br>n = 32	21.4 (6.42)<br>n = 30	30.9 (6.36)<br>n = 29
Melbourne	12.11 (3)<br>n = 19	16.13 (5.83)<br>n = 15	22.79 (6.18)<br>n = 14	28.31 (9.31)<br>n = 13	12.89 (2.42)<br>n = 19	19.29 (6.07)<br>n = 17	23.88 (5.61)<br>n = 17	32.06 (6.23)<br>n = 16
MSEL Visual Reception raw score								
Perth	14.15 (2.34)<br>n = 34	19.7 (3.39)<br>n = 33	24.84 (5.21)<br>n = 31	35.1 (7.25)<br>n = 30	14.12 (2.38)<br>n = 32	19.84 (2.68)<br>n = 32	24.87 (4.44)<br>n = 30	35.41 (7)<br>n = 29
Melbourne	17.39 (2.3)<br>n = 18	21.79 (2.89)<br>n = 14	24.29 (6.37)<br>n = 14	32.62 (9.25)<br>n = 13	17.89 (2.78)<br>n = 18	23.06 (2.61)<br>n = 17	28.41 (4.58)<br>n = 17	36.44 (6.54)<br>n = 16
MSEL Fine Motor raw score								
Perth	13.47 (2.69)<br>n = 34	18.61 (1.95)<br>n = 33	22.13 (3.53)<br>n = 31	30.07 (4.2)<br>n = 30	13.47 (3.01)<br>n = 32	18.78 (1.9)<br>n = 32	22.87 (2.92)<br>n = 30	30.07 (3.86)<br>n = 29
Melbourne	15.63 (2.61)<br>n = 19	19.67 (3.7)<br>n = 15	23.14 (5.56)<br>n = 14	28.31 (5.36)<br>n = 13	16.58 (2.01)<br>n = 19	21.53 (1.55)<br>n = 17	25.29 (2.8)<br>n = 17	31.5 (4.18)<br>n = 16

© 2021 Whitehouse AJO et al. *JAMA Pediatrics.*

VABS-II Communication standard score								
Perth	81.26 (13.5) n = 27	90.79 (14.61) n = 28	93.79 (13.11) n = 24	95.07 (16.02) n = 29	77.68 (15.43) n = 31	91.07 (15.41) n = 29	95.72 (14.14) n = 25	95.96 (14.72) n = 25
Melbourne	78.12 (15.25) n = 17	80.5 (17.33) n = 14	89 (21.81) n = 11	88.64 (27.36) n = 11	76.16 (16.84) n = 19	89.12 (14.84) n = 17	90.65 (16.02) n = 17	92.81 (12.66) n = 16
VABS-II Socialization standard score								
Perth	90.93 (12.32) n = 27	94.58 (12.29) n = 26	93.64 (11.85) n = 25	97.26 (15.07) n = 27	87.59 (10.98) n = 29	94.41 (13.62) n = 29	97.58 (12.48) n = 26	100.93 (16.07) n = 27
Melbourne	91.65 (11.74) n = 17	89.46 (11.98) n = 13	90.91 (22.28) n = 11	88.09 (24.87) n = 11	82.58 (12.1) n = 19	91 (9.43) n = 17	84.69 (12.11) n = 16	87.5 (13.32) n = 16
MCDI Total Expressive Vocabulary								
Perth	1.04 (1.94) n = 23	20.57 (33.06) n = 28	93.96 (96.16) n = 26	426.57 (193.96) n = 28	2.88 (3.31) n = 17	33.88 (49.69) n = 16	147.5 (165.75) n = 16	466.5 (191.64) n = 14
Melbourne	1.67 (2.47) n = 15	10.69 (9.17) n = 13	103.64 (116.45) n = 11	380.8 (232.04) n = 10	0.87 (1.25) n = 23	24.48 (40.24) n = 29	119.07 (116.33) n = 27	430.37 (190.12) n = 27
MCDI Total Receptive Vocabulary								
Perth	20.13 (16.35) n = 15	77.92 (43.56) n = 13	221.09 (133.55) n = 11	494.8 (166.9) n = 10	30.74 (33.71) n = 23	119.97 (84.94) n = 29	263.56 (160.71) n = 27	503.63 (159.24) n = 27
Melbourne	30.22 (37.11) n = 23	103.68 (56.7) n = 28	253.77 (144.41) n = 26	505.18 (165.26) n = 28	38.06 (35.63) n = 17	141.5 (83.3) n = 16	307.38 (172.37) n = 16	555.64 (169.04) n = 14
MCDI Total Gestures								
Perth	10.24 (6.14) n = 25	28.66 (8.12) n = 29	40.76 (12.21) n = 25	52.14 (10.54) n = 28	10.24 (5.2) n = 29	30.72 (10.49) n = 29	43.5 (12.05) n = 22	52.67 (8.31) n = 27

© 2021 Whitehouse AJO et al. *JAMA Pediatrics.*

Melbourne	11.94 (5.81)<br>n = 17	25.43 (10.69)<br>n = 14	33.75 (19.56)<br>n = 12	43 (19.05)<br>n = 11	12.39 (6.57)<br>n = 18	31.29 (11.83)<br>n = 17	39.24 (12.27)<br>n = 17	50.14 (10.65)<br>n = 14
PSOC Efficacy								
Perth	21.63 (4.25)<br>n = 27	22.39 (4.05)<br>n = 28	22.12 (4)<br>n = 24	22.14 (4.23)<br>n = 29	21.36 (4.16)<br>n = 31	21.32 (4.38)<br>n = 31	22.07 (4.92)<br>n = 27	21.73 (4.64)<br>n = 27
Melbourne	21.24 (4.05)<br>n = 17	22.5 (4.78)<br>n = 14	22.27 (4.73)<br>n = 11	22.45 (3.05)<br>n = 11	18.82 (3.25)<br>n = 19	21.62 (2.31)<br>n = 16	20.59 (2.35)<br>n = 17	21.27 (3.86)<br>n = 15
PSOC Interest								
Perth	15.15 (2.27)<br>n = 27	15.89 (2.48)<br>n = 28	15.33 (2.46)<br>n = 24	15.07 (2.53)<br>n = 29	15.39 (2.56)<br>n = 31	14.94 (2.54)<br>n = 31	15.85 (2.11)<br>n = 27	14.76 (3)<br>n = 27
Melbourne	14.47 (2.7)<br>n = 17	14.14 (2.41)<br>n = 14	14.82 (2.79)<br>n = 11	14 (2)<br>n = 11	15.11 (2.56)<br>n = 19	15.56 (2.31)<br>n = 16	15.35 (2.15)<br>n = 17	14.67 (3.48)<br>n = 15
PSOC Satisfaction								
Perth	23.41 (5.55)<br>n = 27	22.68 (6.5)<br>n = 28	22.62 (5.7)<br>n = 24	22.69 (5.71)<br>n = 29	24.23 (4.54)<br>n = 31	24.42 (4.63)<br>n = 31	22.96 (5.04)<br>n = 27	21.78 (6.15)<br>n = 27
Melbourne	22.81 (5.68)<br>n = 17	21.46 (7.34)<br>n = 14	22.55 (5.47)<br>n = 11	21.55 (5.77)<br>n = 11	22.37 (5.34)<br>n = 19	22.19 (3.31)<br>n = 16	22.76 (4.21)<br>n = 17	21.4 (3.74)<br>n = 15

[a]ADOS-2 Toddler module Total Score

[b]ADOS-2 Calibrated Severity Score

Data are mean (SD), with number of participants available. AOSI, Autism Observational Schedule for Infants; ADOS-2, Autism Diagnostic Observation Schedule – 2nd Edition; MACI, Manchester Assessment of Caregiver-Infant Interaction; MSEL, Mullen Scale of Early Learning; VABS-II, Vineland Adaptive Behavior Scale-2nd edition. MCDI: McArthur-Bates Communicative Development Inventory; PSOC: Parenting Sense of Competence.

© 2021 Whitehouse AJO et al. *JAMA Pediatrics.*

eTable 5. Primary and Secondary Outcome Data Across Assessment Points by Sibling Status

	Usual care group				iBASIS-VIPP group			
	Baseline	Treatment endpoint	12-month post-baseline	24-month post-baseline	Baseline	Treatment endpoint	12-month post-baseline	24-month post-baseline
AOSI raw score								
No sibling with ASD	9.32 (4.61)<br>n = 41	9.36 (4.79)<br>n = 36	-	-	9.9 (4.05)<br>n = 40	9.57 (4.59)<br>n = 37	-	-
Sibling with ASD	9.36 (4.48)<br>n = 11	10.1 (6.17)<br>n = 10	-	-	9.18 (3.19)<br>n = 11	7.64 (3.04)<br>n = 11		
ADOS-2								
No sibling with ASD	-	-	10.97 (6.39)<br>n = 36 [a]	5.83 (2.82)<br>n = 36 [b]	-	-	9.81 (6.49)<br>n = 37 [a]	5.31 (2.29)<br>n = 36 [b]
Sibling with ASD	-	-	11.22 (6.61)<br>n = 9 [a]	5 (2.56)<br>n = 8 [b]	-	-	7.9 (3.45)<br>n = 10 [a]	5 (2.35)<br>n = 9 [b]
MACI Caregiver Nondirectiveness								
No sibling with ASD	4.02 (1.49)<br>n = 41	4.76 (1.38)<br>n = 37	4.62 (1.56)<br>n = 34	4.67 (1.45)<br>n = 33	4.53 (1.55)<br>n = 40	4.87 (1.19)<br>n = 38	4.83 (1.52)<br>n = 35	4.94 (1.37)<br>n = 34
Sibling with ASD	4.36 (1.69)<br>n = 11	4.4 (1.58)<br>n = 10	4.5 (1.31)<br>n = 8	4 (1.29)<br>n = 7	3.09 (1.51)<br>n = 11	4.73 (1.27)<br>n = 11	5.1 (1.29)<br>n = 10	4.78 (1.48)<br>n = 9
MACI Sensitive Responding								
No sibling with ASD	4.12 (1.4)<br>n = 41	4.86 (1.06)<br>n = 37	4.44 (1.26)<br>n = 34	4.64 (1.14)<br>n = 33	4.42 (1.47)<br>n = 40	5.05 (0.9)<br>n = 38	4.66 (1.28)<br>n = 35	4.85 (1.4)<br>n = 34
Sibling with ASD	4.82 (1.54)<br>n = 11	4.6 (1.07)<br>n = 10	4.5 (1.2)<br>n = 8	4.57 (1.4)<br>n = 7	3.64 (1.5)<br>n = 11	5 (1)<br>n = 11	5.1 (1.29)<br>n = 10	4.56 (1.74)<br>n = 9
MACI Infant Attentiveness								
No sibling with ASD	3.88 (1.33)<br>n = 41	4.78 (1.06)<br>n = 37	4.18 (1.29)<br>n = 34	5.12 (1.17)<br>n = 33	3.95 (1.2)<br>n = 40	4.45 (1.18)<br>n = 38	4.66 (1.14)<br>n = 35	5.09 (1.19)<br>n = 34
Sibling with ASD	4.55 (1.44)<br>n = 11	4.4 (1.07)<br>n = 10	4.25 (1.16)<br>n = 8	5.29 (0.76)<br>n = 7	3.45 (1.21)<br>n = 11	4.36 (1.12)<br>n = 11	4.4 (1.17)<br>n = 10	4.78 (1.09)<br>n = 9

© 2021 Whitehouse AJO et al. *JAMA Pediatrics.*

MACI Infant Positive Affect								
No sibling with ASD	3.51 (1.65)<br>n = 41	4.32 (1.38)<br>n = 37	3.29 (1.98)<br>n = 34	4.06 (1.97)<br>n = 33	3.33 (1.42)<br>n = 40	3.47 (1.31)<br>n = 38	3.11 (2.05)<br>n = 35	3.88 (2.04)<br>n = 34
Sibling with ASD	3.45 (2.11)<br>n = 11	4.7 (1.16)<br>n = 10	2.88 (1.64)<br>n = 8	5.29 (1.7)<br>n = 7	3.27 (1.85)<br>n = 11	4.45 (2.07)<br>n = 11	3.4 (1.96)<br>n = 10	4.56 (1.59)<br>n = 9
MSEL Expressive Language raw score								
No sibling with ASD	9.68 (2.42)<br>n = 41	14.71 (3.36)<br>n = 38	19.56 (5.08)<br>n = 36	29.57 (6.79)<br>n = 35	10.03 (2.36)<br>n = 40	15.47 (3.38)<br>n = 38	20.92 (5.66)<br>n = 37	31.14 (8.1)<br>n = 36
Sibling with ASD	9 (3.03)<br>n = 11	15.9 (4.31)<br>n = 10	19.78 (6.83)<br>n = 9	28.75 (9.65)<br>n = 8	9.36 (2.25)<br>n = 11	14.91 (3.59)<br>n = 11	21.8 (5.57)<br>n = 10	30.22 (4.29)<br>n = 9
MSEL Receptive Language raw score								
No sibling with ASD	10.95 (2.84)<br>n = 41	15.61 (4.21)<br>n = 38	22.03 (5.91)<br>n = 36	29.71 (7.35)<br>n = 35	11.15 (2.65)<br>n = 40	17.29 (5.32)<br>n = 38	22.78 (6.42)<br>n = 37	31.5 (6.83)<br>n = 36
Sibling with ASD	11.36 (3.23)<br>n = 11	14.5 (5.58)<br>n = 10	21.67 (5.05)<br>n = 9	29.25 (8.88)<br>n = 8	9.64 (3.38)<br>n = 11	14.82 (5.21)<br>n = 11	20.5 (5.19)<br>n = 10	30.56 (3.36)<br>n = 9
Sibling with ASD								
No sibling with ASD	15.1 (2.85)<br>n = 40	20.03 (3.51)<br>n = 37	24.25 (5.24)<br>n = 36	34.23 (7.32)<br>n = 35	15.64 (3.09)<br>n = 39	21.21 (2.82)<br>n = 38	26.46 (4.8)<br>n = 37	35.97 (7.24)<br>n = 36
Sibling with ASD	16.09 (2.51)<br>n = 11	21.4 (2.59)<br>n = 10	26.33 (6.63)<br>n = 9	34.88 (10.58)<br>n = 8	14.91 (3.21)<br>n = 11	20.09 (3.75)<br>n = 11	25 (4.69)<br>n = 10	35 (4.77)<br>n = 9
MSEL Fine Motor raw score								
No sibling with ASD	14.12 (2.71)<br>n = 41	18.71 (2.46)<br>n = 38	22.14 (4.39)<br>n = 36	29.63 (4.37)<br>n = 35	14.93 (2.83)<br>n = 40	19.97 (1.95)<br>n = 38	23.86 (3.07)<br>n = 37	30.86 (4.13)<br>n = 36
Sibling with ASD	14.73 (3.47)<br>n = 11	19.8 (3.19)<br>n = 10	23.67 (3.43)<br>n = 9	29.12 (5.79)<br>n = 8	13.55 (3.72)<br>n = 11	18.91 (2.88)<br>n = 11	23.3 (3.23)<br>n = 10	29.44 (3.32)<br>n = 9
VABS-II Communication standard score								

© 2021 Whitehouse AJO et al. *JAMA Pediatrics.*

No sibling with ASD	81.18 (13.75) n = 34	89.12 (13.69) n = 33	93.86 (15.54) n = 29	95.56 (17.93) n = 32	78.9 (15.43) n = 39	91.89 (14.92) n = 37	94.85 (14.82) n = 34	95.39 (14.9) n = 33
Sibling with ASD	74.33 (15.06) n = 9	80.89 (22.84) n = 9	84.67 (18.45) n = 6	84.25 (24.54) n = 8	70.73 (16.29) n = 11	84 (14.77) n = 9	88.62 (15.42) n = 8	92 (8.68) n = 8
VABS-II Socialization standard score								
No sibling with ASD	92.26 (12.53) n = 34	94.13 (12.18) n = 31	95.03 (14.77) n = 30	98.43 (16.06) n = 30	86.45 (11.55) n = 38	93.43 (10.81) n = 37	94.43 (13.22) n = 35	96.8 (17.02) n = 35
Sibling with ASD	86.89 (9.71) n = 9	88 (12.18) n = 8	81.67 (15.29) n = 6	80.25 (21.32) n = 8	82.4 (11.71) n = 10	92 (17.75) n = 9	83.86 (13.79) n = 7	92.12 (13.04) n = 8
MCDI Total Expressive Vocabulary								
No sibling with ASD	1.29 (2.3) n = 31	15.36 (26.68) n = 33	102.58 (105.33) n = 31	418.77 (210.86) n = 31	1.94 (2.73) n = 33	27.08 (40.11) n = 36	138.74 (149.39) n = 34	448.71 (203.66) n = 34
Sibling with ASD	1.33 (1.63) n = 6	26 (33.55) n = 8	67.17 (75.25) n = 6	395.71 (172.63) n = 7	0.71 (0.76) n = 7	30.78 (57.97) n = 9	95.33 (52.71) n = 9	413.57 (94.29) n = 7
MCDI Total Receptive Vocabulary								
No sibling with ASD	28.68 (33.08) n = 31	97.42 (55.14) n = 33	253.39 (139.45) n = 31	502.52 (171.01) n = 31	38.42 (35.95) n = 33	135.61 (80.65) n = 36	310.03 (166.03) n = 34	522.82 (173.93) n = 34
Sibling with ASD	17.67 (13.66) n = 6	87.62 (50.07) n = 8	195.83 (146.94) n = 6	502.14 (136.34) n = 7	12.29 (9.46) n = 7	95.67 (94.71) n = 9	165.89 (99.35) n = 9	514.43 (97.45) n = 7
MCDI Total Gestures								
No sibling with ASD	11.38 (5.73) n = 32	28.12 (9.12) n = 34	40.87 (13.76) n = 30	51.55 (12.15) n = 31	11.79 (5.45) n = 38	31.43 (9.93) n = 37	43.06 (11.8) n = 32	52.03 (9.12) n = 34
Sibling with ASD	10.11 (7.04) n = 9	25.67 (8.96) n = 9	28.29 (17.31) n = 7	41.88 (17.98) n = 8	8 (6.52) n = 9	28.89 (14.7) n = 9	35.14 (12.64) n = 7	50.71 (9.76) n = 7
PSOC Efficacy								
No sibling with ASD	21.5 (4.38) n = 34	22.09 (3.82) n = 33	22 (4.3) n = 29	22.47 (4.1) n = 32	20.62 (3.97) n = 40	21.81 (3.63) n = 37	21.63 (4.39) n = 35	21.49 (4.32) n = 34

© 2021 Whitehouse AJO et al. *JAMA Pediatrics.*

Sibling with ASD	21.67 (3.46) n = 9	23.67 (5.66) n = 9	23 (3.74) n = 6	21.25 (3.06) n = 8	19.5 (4.25) n = 10	20 (4.16) n = 10	21 (3.24) n = 9	21.88 (4.7) n = 8
PSOC Interest								
No sibling with ASD	14.65 (2.32) n = 34	15.33 (2.51) n = 33	14.97 (2.54) n = 29	14.69 (2.4) n = 32	15.32 (2.67) n = 40	15.49 (2.23) n = 37	15.97 (1.95) n = 35	14.66 (3.2) n = 34
Sibling with ASD	15.67 (2.92) n = 9	15.22 (2.95) n = 9	16.17 (2.48) n = 6	15.12 (2.64) n = 8	15.1 (2.02) n = 10	13.9 (2.96) n = 10	14.44 (2.4) n = 9	15 (3.02) n = 8
PSOC Satisfaction								
No sibling with ASD	23.61 (5.75) n = 34	22.56 (6.51) n = 33	22.79 (5.89) n = 29	23.09 (5.31) n = 32	23.38 (5.09) n = 40	23.84 (4.52) n = 37	23.4 (4.48) n = 35	21.74 (5.47) n = 34
Sibling with ASD	22.11 (4.83) n = 9	21.22 (7.81) n = 9	21.67 (3.67) n = 6	19.5 (6.55) n = 8	24.1 (4.2) n = 10	23 (3.65) n = 10	20.89 (5.21) n = 9	21.25 (5.26) n = 8

[a]ADOS-2 Toddler module Total Score

[b]ADOS-2 Calibrated Severity Score

Data are mean (SD), with number of participants available. AOSI, Autism Observational Schedule for Infants; ADOS-2, Autism Diagnostic Observation Schedule – 2nd Edition; MACI, Manchester Assessment of Caregiver-Infant Interaction; MSEL, Mullen Scale of Early Learning; VABS-II, Vineland Adaptive Behavior Scale-2nd edition. MCDI: McArthur-Bates Communicative Development Inventory; PSOC: Parenting Sense of Competence.

© 2021 Whitehouse AJO et al. *JAMA Pediatrics.*

eFigure 1. Forest Plot of Comparison Between Treatment Groups by Autism Spectrum Disorder Diagnostic Criteria

Forest plot showing comparison between treatment groups (odds ratio, one-sided 95% confidence intervals) on clinical ASD diagnosis, and each DSM-5 criterion for ASD. Estimates are adjusted for infant age at the 24-month post-baseline assessment (3-years-of-age), baseline AOSI scores, and infant sex. An adjusted estimate less than 1 indicates a reduced likelihood of the iBASIS-VIPP group meeting criteria compared with the usual care group.

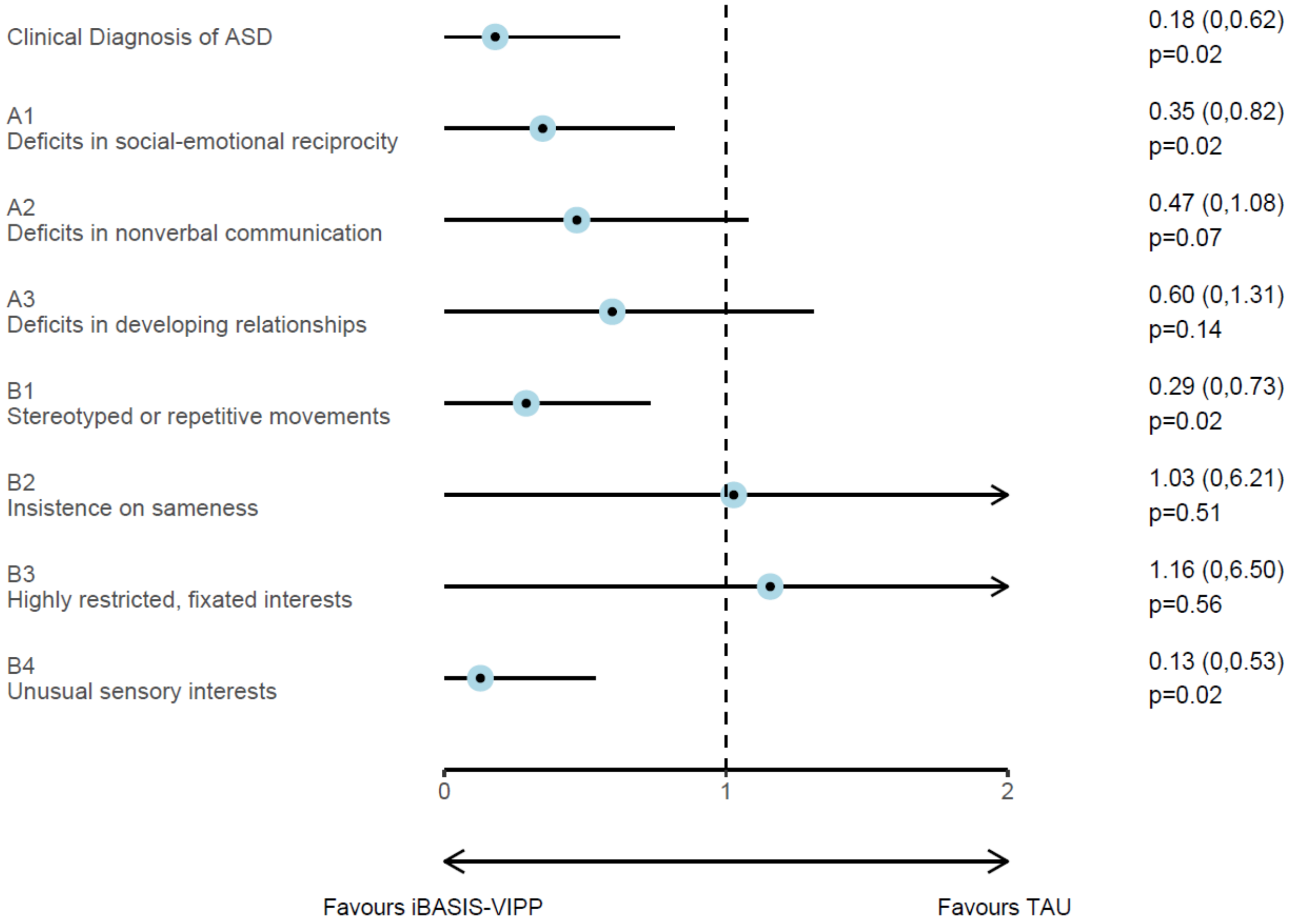

© 2021 Whitehouse AJO et al. *JAMA Pediatrics.*

eFigure 2. Area Between Curves for Secondary Outcomes

Area between the curve figures for secondary outcomes. An area-between-the-curves (shaded area) above the null indicates improved performance on a given measure in the iBASIS-VIPP group compared with the usual care group.

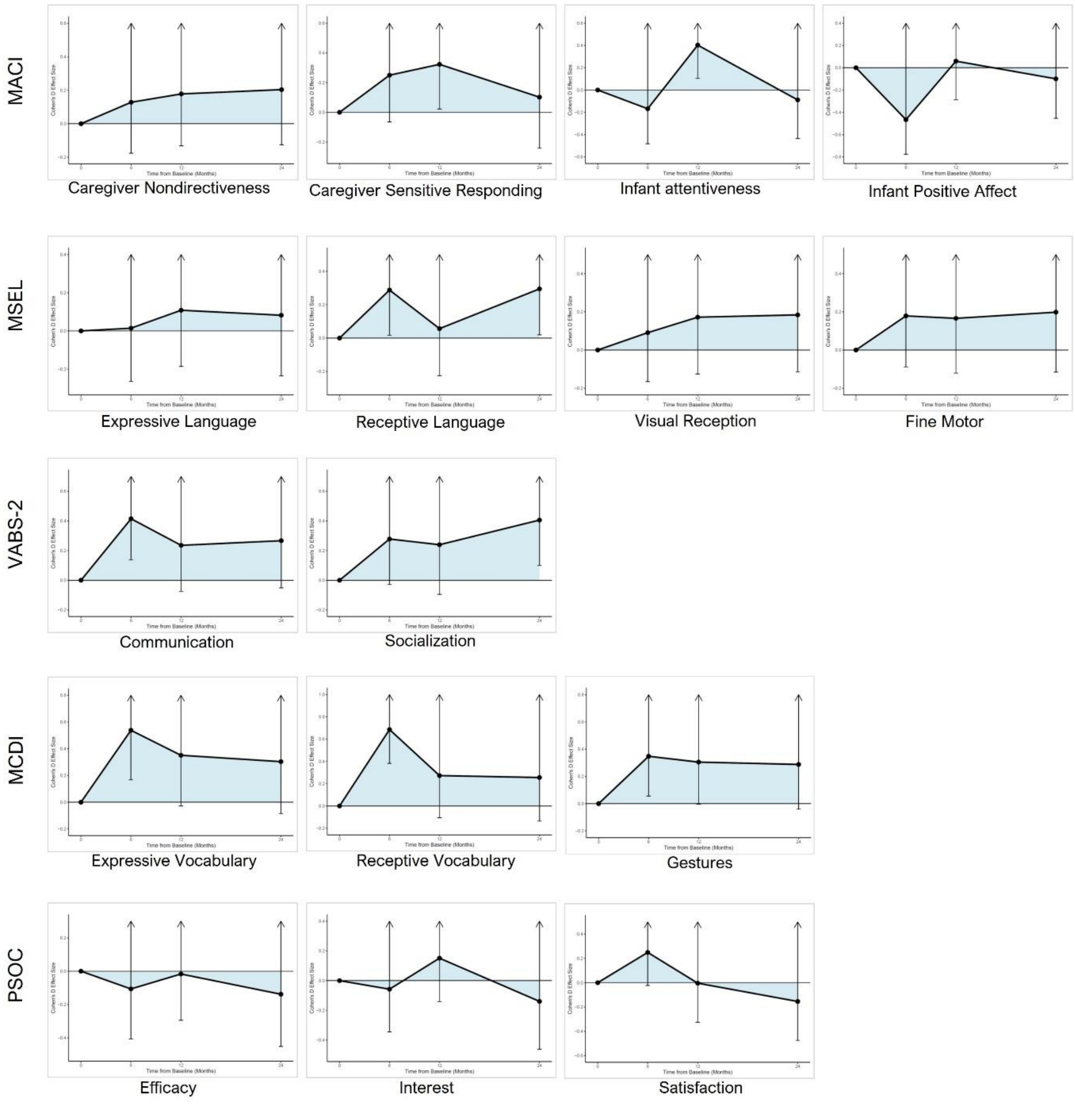

Abbreviations: MACI, Manchester Assessment of Caregiver-Infant Interaction; MSEL, Mullen Scale of Early Learning; VABS-II, Vineland Adaptive Behavior Scale-2nd edition. MCDI: McArthur-Bates Communicative Development Inventory; PSOC: Parenting Sense of Competence.

© 2021 Whitehouse AJO et al. *JAMA Pediatrics.*

eReferences

1. Juffer F, Bakerman-Kranenburg, MJ, Van Ijzendoorm Van MJ. Promoting positive parenting: An attachment-based intervention. New York: Taylor Francis; 2008.
2. Green J, Charman T, McConachie H et al. Parent-mediated communication-focused treatment in children with ASD. Lancet. 2010; 375: 2152.
3. Pickles A, Le Couteur A, Leadbitter K. et al. Parent-mediated social communication therapy for young children with autism (PACT): Long-term follow-up of a randomised controlled trial. The Lancet 2016; 388: 2501–2509.
4. Green J, Wan M, Guiraud J, et al. Intervention for infants at risk of developing autism. J Autism Dev Disord. 2013;43(11):2502-14.
5. Green J, Charman T, Pickles A, et al. Parent-mediated intervention versus no intervention for infants at high risk of autism. Lancet Psychiatr. 2(2):133-40.
6. Green J, Pickles A, Pasco G, et al. Randomised trial of a parent-mediated intervention for infants at high risk for autism: longitudinal outcomes to age 3 years. J Child Psychol Psychiatr. 2017;58(12):1330-40.
7. Barbaro J, Dissanayake C. Prospective identification of autism spectrum disorders in infancy and toddlerhood using developmental surveillance: The Social Attention and Communication Study. J Dev Behav Pediatr 2010; 31: 376-385.
8. Barbaro J, Dissanayake C. Prospective identification of autism spectrum disorders in infancy and toddlerhood using developmental surveillance: The Social Attention and Communication Study. J Dev Behav Pediatr 2010; 31: 376-385.
9. Barbaro J, Dissanayake C, Sadka N, et al. Universal developmental surveillance for autism in infants, toddlers and preschoolers: The Social Attention and Communication Study-Revised (SACS-R) and SACS-Preschool. In: INSAR - International Society for Autism Research Annual Meeting. Rotterdam, Netherlands; 2018.
10. Mozolic-Staunton B, Donelly M, Yoxall J, Barbaro J. Early detection for better outcomes: universal developmental surveillance for autism across health and early childhood education settings. Res Autism Spectr Disord; 71: 101496.
11. Whitehouse AJO, Varcin KJ, Alvares GA, et al. Pre-emptive intervention versus treatment as usual for infants showing early behavioral risk signs of autism spectrum disorder: a single-blind, randomised controlled trial. Lancet Child Adoles Health 2019; 3(9):605–615.
12. Bryson S, Zwaigenbaum L, McDermott C, Rombough V, Brian J. The Autism Observation Scale for Infants: Scale development and reliability data. J Autism Dev Disord. 2008;38(4):731-8.
13. Bussu G, Jones EJH, Charman T, et al. Prediction of autism at 3 years from behavioural and developmental measures in high-risk infants: A longitudinal cross-domain classifier analysis. J Autism Dev Disord. 2018;48(7):2418-33.
14. Lord C. et al. Autism Diagnostic Observation Schedule, 2nd Edn.,(ADOS-2) Manual (Part 1): Modules 1–4, Torrance, CA: Western Psychological Services; 2021.
15. Luyster R, Gotham K, Guthrie W, et al. The Autism Diagnostic Observation Schedule - Toddler Module. J Autism Dev Disord 2009;39(9):1305-20.
16. Gotham K, Pickles A, Lord C. Standardizing ADOS scores for a measure of severity in autism spectrum disorders. J Autism Dev Disord 2009;39(5):693–705.
17. Wan MW, Brooks A, Green J, Abel K, Elmadih A. Psychometrics and validation of a brief rating measure of parent-infant interaction: Manchester assessment of caregiver–infant interaction. Int J Behav Dev 2016; 41: 542–49.
18. Wan MW, Green J, Elsabbagh M, et al. Quality of interaction between at-risk infants and caregiver at 12–15... J Child Psychol Psyc 2013;54(7):763-71.

© 2021 Whitehouse AJO et al. *JAMA Pediatrics.*

19. Mullen EM. Mullen's scales of early learning. Circle Pines, MN: American Guidance Service;1995.
20. Akshoomoff N. Use of the Mullen Scales of Early Learning for the assessment of young children with autism spectrum disorders. Child Neuropsychol 2006;12(4-5):269–277
21. Sparrow SS, Cicchettii DV, Balla DA. The Vineland Adaptive Behavior Scales, 2nd Edition (VABS-II). NCS Pearson Inc. 2005.
22. Fenson L, Dale PS, Reznick JS. The MacArthur Communicative Development Inventories: User's Guide and Technical Manual. San Diego, CA: Singular Publishing Group; 1993.
23. Gilmore LA Cuskelly M. Factor structure of the Parenting Sense of Competence Scale using a normative sample. Child Care Health Dev2008; 38: 48-55.

© 2021 Whitehouse AJO et al. *JAMA Pediatrics.*
