# Supplementary material for: Effect of Preemptive Intervention on Developmental Outcomes Among Infants Showing Early Signs of Autism: A Randomized Clinical Trial of Outcomes to Diagnosis
Source: JAMA Pediatr. 2021 Sep 20;175(11):e213298. doi: 10.1001/jamapediatrics.2021.3298 (PMC8453361; doi:10.1001/jamapediatrics.2021.3298)
Supplement: Supplement 3. — Data Sharing Statement [file jamapediatr-e213298-s003.pdf]

Data Sharing Statement

Whitehouse. Effect of Preemptive Intervention on Developmental Outcomes Among Infants Showing Early Signs of Autism. *JAMA Pediatr*. Published September 20, 2021. doi:10.1001/jamapediatrics.2021.3298

Data
Data available: Yes
Data types: Deidentified participant data, Data dictionary
How to access data: Proposals should be directed to Andrew.Whitehouse@telethonkids.org.au.
When available: With publication

Supporting Documents
Document types: None

Additional Information
Who can access the data: Investigators whose proposed use of the data has been approved by an independent ethical review committee identified for this purpose.
Types of analyses: For replication studies and individual participant data meta-analysis.
Mechanisms of data availability: Data requestors will be requested to sign a data access agreement
